# Electrolytic copper as cheap and effective catalyst for one-pot triazole synthesis

**DOI:** 10.1038/s41598-018-22703-0

**Published:** 2018-03-14

**Authors:** Jacek Mularski, Barbara Czaplińska, Wioleta Cieślik, Jakub Bebłot, Piotr Bartczak, Rafał Sitko, Jarosław Polański, Robert Musiol

**Affiliations:** Institute of Chemistry, University of Silesia, Szkolna 9, Katowice, 40-006 Poland

## Abstract

Electrolytic copper is a well-known form of pure, oxygen free copper that is used for industrial applications. In this work, the catalytic potential of this relatively cheap material was studied. The addition of less than 0.015 mol equivalent of copper powder effectively catalysed the one-pot synthesis of triazoles from a diverse range of organic halides and alkynes. Quantitative conversions in aqueous solvents can be achieved within minutes. The heterogenous nature of the catalyst afforded a low level of copper contamination in the products, thus meeting the rigorous criteria of the pharmaceutical industry.

## Introduction

The 1,3-dipolar cycloaddition between an azide and alkyne is a prime example of a click chemistry reaction. This approach allows 1,4 or 1,5-substituted 1,2,3-triazoles to be created in an elegant way^1,2^. These important molecular scaffolds are not readily available through alternative methods^3^. Triazoles have become crucial in medicinal chemistry and drug synthesis where biologically oriented patterns do not comply with the products of typical carbonyl chemistry^2^.

The discovery of the catalytic properties of copper^4^ or ruthenium compounds in these reactions has extended their synthetic utility. Copper (II) sulfates and acetates combined with sodium ascorbate under aqueous condition are especially favorable for generating Cu(I) *in situ* in order to create intermediate acetylides required for the dipolar cycloaddition. Water appears to be an ideal solvent to stabilize copper(I) acetylides against aggregated, unreactive forms^5^. Such protocols have been widely adopted due to their effectiveness and versatility. At the same time, however, the use of toxic heavy metals in aqueous solutions cause environmental concerns. Dissolved metals affect the costs of the synthesis, particularly in medicinal chemistry and pharmaceutical applications where strict requirements regulate the level of heavy-metal impurities in pharmaceutical materials. Metal scavenging is another costly step, which also affects the R&D operational field. However, it is essential to eliminate biocidal copper from stocks that are designated for biological screening and therapeutic applications. Thus, alternatives to homogenous copper salt catalysts are Cu(I) species that are immobilized on solid supports such as silica, charcoal or zeolites^6^. Recently, Cu nanoparticles have also been described in this regard. Kumar *et al*. reported the synthesis of triazoles from azides and alkynes in the presence of Cu nanoparticles that were suspended in guar gum^7^. Copper (II) oxide (CuO) has also proven to be effective as a nano-catalyst in Huisgen cycloadditions^8^. To date, various nanoparticles have been prepared and tested for click chemistry synthesis^9–13^. Copper nano-catalysts overcome several drawbacks of homogenous catalysis using Cu(I) soluble compounds. Notably, single-molecule spectroscopy supports a heterogeneous-catalysed mechanism of reaction^14^. Nevertheless, this approach still increases the costs of the synthesis. Moreover, some Cu nanoparticles are not free from stability problems^13,15,16^.

From a practical point of view, the discovery that metallic copper may, under specific conditions, provide an effective catalyst for dipolar cycloadditions, was a great advance. Cintas *et al*. described an approach that exploited ultrasound irradiation alone or in combination with microwave irradiation^17^ with copper turnings^18^. These approaches allowed saving time in comparison to conventional heating. Mechanical activation such as grinding with Cu powder under solvent-free conditions has also been be used^19^. On the other hand however latest report of Tumanov *et al*. suggest the tiny amount of water is crucial in such conditions^20^. These facts prompted us to investigate electrolytic copper as the easily available and cheap heterogenous catalyst candidate that combines all of the above described benefits. Our results confirm its superior performance in terms of efficiency and economy.

## Results

In our experiments, we selected a series of structurally diverse substrates including aromatic and aliphatic halides to test the overall activity of this catalyst. The experimental procedure consisted of mixing a benzyl bromide with sodium azide and a terminal acetylene group in water in the presence of electrolytic copper powder. The resulting mixture was heated at 85 °C until the substrates disappeared (TLC monitoring), then reaction was terminated and product isolated to provide yields that are presented in Table 1.

**Table 1:** One-pot triazole synthesis^a^.

substrates		Phenylacetylene a	Hex-1-yne b	Propargyl alcohol c
		time[h]/yield[%]	time [h]/yield [%]	time [h]/yield [%]
BnBr	**1**	B; 2/97^b,c^	B; 3/90^b^	B; 2/96
*p*-Br·C_6_H_4_-CH_2_Br	**2**	B; 2/99	A; 1/99^d^	A; 1/94
*p*-Cl·C_6_H_4_-CH_2_Br	**3**	B; 3/99	A; 1/72	A; 1/96
*p*-I·C_6_H_4_-CH_2_Br	**4**	B; 2/99^b^	B; 2/99^b^	A; 2/99^b^
2,3-Cl·C_6_H_3_-CH_2_Br	**5**	B; 2/93	B; 2/99	B; 2/99
n-BuBr	**6**	A; 24/94^b,c^	—	—
Allyl iodide	**7**	A; 1/99	—	—
ethyl chloroacetate	**8**	A; 48/91^b,d^	—	—
4-bromobutyl acetate	**9**	A; 24/98	—	
2-bromo-1-(4-methylphenyl)propan-1-one	**10**	A; 48/94^e^	—	A; 48/96^e^
1-fluoro-4-nitrobenzene	**11**	A; 24/8^b^	—	A; 24/10^b^
2-(bromomethyl)quinoline	**12**	B; 0.5/85^c^	B; 0.5/85^c^	B; 0.5/88^c^

^a^General conditions: Copper powder, bromide, sodium azide and water. ^b^Optionally 10% (v/v) of t-BuOH was added. ^c^Conditions B, alkyne was added into the reaction mixture by injection through a syringe after substrates’ premixing. ^d^Conditions A: Alkyne was added into the reaction mixture before vial capping. ^d^24 h at RT then 24 h, 85 °C. ^e^RT; Isolated yields.

The most commonly used solvent in this dipolar cycloaddition is water. Mechanistically, the hydrogen bonding potential between water molecules and substrates’ atoms is the reason for the unique reactivity of the organic compounds in this medium^21^ and may be responsible in some part for the initial removal of proton during the formation of intermediate acetylides. The reaction rate is enhanced even if insoluble substrates are stirred in an aqueous suspension^22^. In this case however, 10% addition of t-butyl alcohol improved the reaction. These reaction procedures appeared effective in synthesis of quinoline and quinazoline conjugates as is shown in Table 2.

**Table 2:** Quinoline and quinazoline triazole analogs^a^.

Substrates		2-(bromomethyl)-8-chloroquinoline	2-(bromomethyl)-8-chloroquinoline	2-(bromomethyl) quinazolin-4(3*H*)-one
		**13**	**13** ^**b**^	**14**
		time [h]/yield [%]	time [h]/yield [%]	time [h]/yield [%]
Phenylacetylene	**a**	B; 0.08/50	C; 24/71	B; 5/73^c^
Hex-1-yne	**b**	A; 24/65^c^	C; 72/98	B; 1.5/73^c^
2-propyn-1-ol	**c**	B; 0.25/65	C; 24/69	B; 1/70^c^
3-butyn-2-ol	**d**	B; 0.35/50	C; 24/75	B; 0.5/72^c^
Benzyl propargyl ether	**e**	B; 0.5/60	C; 72/81	B; 0.25/95^c^
4-Ethynyl-*N*,*N*-dimethylaniline	**f**	B; 0.65/52	C; 72/72	B; 0.25/88^c^

^a^Reaction conditions: see footnotes of Table 1. ^b^Classical CuAAC method: (0.5 mmol of organic azide and 1 mmol of alkyne) 6 cm^3^ of t-BuOH, 10 mg L-ASC Na, Cu_2_(OAc)_4_(H_2_O)_2_ 10 mg, Chromatografic sep. ^c^10% of t-BuOH was added. Isolated yields.

### Organic Azide Precursors

Good reactivity of the allyl iodide (Table 1) encouraged us to test other iodide as substrate. However iodides are used only accidentally for their higher costs in some cases, can be beneficial. For example 2-(iodomethyl)-4(*3H*)quinazolinone can be obtained easier with higher yield than corresponding bromide. To investigate reactivity substrates were reacted with TLC monitoring until full conversion, which was then confirmed based on ^1^H NMR of a crude product obtained by filtration. The results are presented in Table 3.

**Table 3:** Comparison of iodides and bromides as substrates in one-pot triazole synthesis^a^.

Substrates^b^		2-(bromomethyl) quinazolin-4(3*H*)-one	2-(iodomethyl) quinazolin-4(3*H*)-one
		**14**	**15**
		Time [h]	
Phenylacetylene	**a**	5	24
Hex-1-yne	**b**	1.5	24
2-propyn-1-ol	**c**	1	24
3-butyn-2-ol	**d**	0.5	24

^a^All of the conversions >99%, ^b^10% (v/v) t-BuOH was added.

### Catalyst Efficiency

Efficiency and reusability of the catalyst was tested subsequently on the exemplary reaction. In short, 2 cm^3^ of water per equimolar 1 mmol of BnBr, and sodium azide was stirred in 85 °C with TLC monitoring to complete conversion, which was then confirmed by ^1^H NMR spectra of crude product. Work-up procedure was extraction with 3 × 50 cm^3^ of methylene chloride and concentration in vacuo. Additionally, we tested catalyst reusability. After the reaction product was transferred in hot ethyl acetate and the solvent was removed. Residuals was washed with DMF, then with water. We found thus clean copper catalyst which was then recycled. Results are presented in (Table 4).

**Table 4:** Catalyst efficiency.


Cu [eq. mol]	Conversion [%]	Time [h]
0.01	99	2
0.01^a^	99	2
0.01^a^	99	2
0.01^a^	99	2
4 × 10^–4^	99	2
2 × 10^–4^	56	24
1 × 10^–4^	22	24

^a^catalyst reused.

Homogenous Cu(I) catalysis may lead to a product that is considerably contaminated with residual copper^23^. Heterogenous catalysts are known to afford cleaner products. This prompted us to evaluate the residual copper after a typical isolation and purification procedure. We used energy-dispersive X-ray fluorescence (EDXRF) spectroscopy to measure the copper level in the crude product (filtered from the reaction media) and after one of purification steps, as presented in Table 5.

**Table 5:** Experiment for the removal of copper particulates from crude triazole^a^.

Purification method	Cu [ppm]
Filtrated crude product	967
Centrifuged (5000 RPM)	270
Filtrated (20 nm pores)	67
Recrystallized from EtOAc	11

^a^Triazole (**1a**) was synthesized following conditions B: BnBr, Phenylacetylene, 0.015 eq of copper catalyst, 2 h, 85 °C.

## Discussion

In general electrolytic copper appeared very effective in catalyzing triazole formation in one-pot cascade synthesis. As presented in Table 1 most of the isolated yields reached quantitative level. Interestingly compounds **11a** and **11c** were obtained in low yield. On the other hand these have been reported only as trace of product in former work of Kafle and Handy^24^.

The Cu(II) reduction step, which is essential during conventional procedure, has to be performed before the addition of alkyne^5^. This require specific regime of the procedure with subsequential mixing of the reagents. In heterogenic Cu catalyst this should be irrelevant. To verify this hypothesis, we performed several experiments by mixing all of the chemicals immediately and then engaging the heat (condition A). Others were performed by introducing the alkyne in the final step after the temperature of the reaction mixture had reached 85 °C (condition B). No significant differences were observed between the outcomes of these two procedures. No apparent change was noted during one hour of agitation at 85 °C when the terminal alkyne was introduced into the aqueous suspension of electrolytic copper. The copper particles form a sediment and the alkyne phase floated. After a solution of sodium azide and organic bromide was introduced to this mixture, the triazole product was formed quantitatively. This experiment indicates that the alkyne substrate may be introduced before or following the addition of bromide and sodium azide.

The same reaction procedure when needed was modified by addition of co-solvent was successfully applied to synthesis of quinolines and quinazolines where it shows advantage over conventional approach. As presented in Table 2 syntheses can be successfully completed in short time. The exceptions were compounds **13b** and **14a**, that required a considerably longer heating time. Quinazoline derivatives have a tendency to aggregate in aqueous solution, thus all reactions with this substrate require the presence of a *t*-BuOH.

The reports on one-pot triazole synthesis using organic halides as the starting material have mainly focused on bromides. We decided to test if iodides can be used in this reaction. Iodides as precursors have limited value for industrial application, due to high costs. From the point of view small scale laboratory synthesis, however it is interesting to test such possibility. Particularly in our experience with heteroaromatic moieties as quinoline and quinazoline where iodides can be obtained easier with higher yields. For example 2-(iodomethyl)quinolines are attractive alternative to the corresponding bromides^25,26^. On the other hand, concerns about the formation of 5-iodotriazoles have been outlined^27–29^. Moreover chemical system that contains copper, iodine and alkyne species is known susceptible to conglomeration^30^. One example of a one-pot triazole synthesis that engages an organic iodide (2 eq. of benzyl iodide, 1 eq. of terminal alkyne) describes the use of DMSO as the solvent, 0.16 eq of Et_3_N as the adjuvant, 0.16 eq of copper (I) iodide as the catalyst and 0.16 eq of proline as a copper ligand^31^. Two quinazolinone halides **14** and **15** were selected for this experiment. Reactions were conducted until complete conversions and time is presented in Table 3. Those results suggest that iodides can be as effective as bromides ensuring high conversions and yields even if required considerably longer reaction times.

Metal-catalyzed chemical processes used on a technical or industrial scale are commonly based on heterogeneous catalysts as these facilitate the work-up operations and provide virtually metal-free products. Such catalysts can be expensive and are usually adapted to custom layouts. Thus their reusability is of high economic priority. In the case of electrolytic copper the advantage lies in its price. To say, recycling of cheap and readily available catalyst may be economically unreasonable. Nevertheless it is important for describing its applicability and may cast a light on mechanistic features. For example digestion of the catalytic surface may lead to loss of activity. Moreover possibility to reuse the catalyst is highly recommended for green chemistry principles. During our experiments electrolytic copper appeared to retain its activity in subsequent conversions.

Another appealing property of heterogenic catalysis is a tendency to provide cleaner products, not contaminated by heavy metals. This is of special importance in drug synthesis where pharmacopeal regulations very strictly limits level of copper^32^. Although the crude product, filtered from our reaction mixture had a significant amount of Cu (0.1%), it can be considerably diminished using simple purification steps. A single crystallization from ethyl acetate afforded a pure product. Residual amount of copper in obtained product is lower than FDA guideline limits that suggest 250 ppm for oral administration and 25 ppm for parenteral use^32^.

Electrolytic copper powder is an effective catalyst for cascade triazole synthesis. There are several advantages in using this electrolytic copper catalyst. Its price and wide availability makes it interesting alternative to specific nanoparticulated versions. Powder of copper catalyst could be also isolated and reused maintaining its high activity. There is no need to use a reducing agent to maintain catalytic activity. In addition, the mixing order does not affect the reaction as it does in conventional homogenous click chemistry. Organic iodides may be used as well as bromides according to availability and needs. Simple isolation and purification resulted in pure products that had small amount of residual copper and thus are suitable for biological testing. What is noticeable use of electrolytic copper considerably reduce amounts of wastes, particularly water insoluble, organic phase. Another appealing aspects of these new method are equimolar amounts of substrates and overall simplicity of the procedure with less steps and chemicals in use.

## Methods

### Materials

The Fourier transform nuclear magnetic resonance spectra of the sample solutions were obtained using a Bruker Avance 400 spectrometer for ^1^H (400 MHz), ^13^C{1H} (101 MHz) and a Bruker Ascend 500 for ^1^H (500 MHz), ^13^C{1H} (126 MHz). Chemical shifts were reported in δ units (parts per million) relative to tetramethylsilane and the residual solvent peaks were used as a reference. High-resolution mass spectra were measured using a DionexUltiMate® 3000 high-performance liquid chromatograph (Thermo Fisher Scientific, West Palm Beach, FL, USA) coupled with an LTQ Orbitrap XL™ Hybrid Ion Trap-Orbitrap Fourier Transform Mass Spectrometer (Thermo Fisher Scientific) with an injection into HESI II in the positive or negative modes. Chromatographic separations were done on a Teledyne ISCO Combiflash Rf150+ . The melting point was determined on an MPA100 Melting Point Apparatus from Stanford Research Systems. The chemical analysis was performed using an Epsilon 3 energy-dispersive X-ray fluorescence (EDXRF) spectrometer (Panalytical, Almelo, The Netherlands) with a Rh target X-ray tube operating at the max. voltage of 30 keV and max. power of 9 W. The spectrometer was equipped with a thermoelectrically cooled silicon drift detector (SDD) with an 8 μm Be window and a resolution of 135 eV at 5.9 keV. The quantitative analysis was performed using Omnian software and based on the fundamental parameter method for matrix correction. The copper was determined using K-alphaline and the following measurement conditions: 30 kV, 120 s counting time, air atmosphere and a 100 μm Ag primary beam filter. The current of the X-ray tube was fixed to not exceed a dead-time loss of ca. 50%.

The materials, which were purchased from commercial sources, were used without further purification: 1-hexyne, propargyl alcohol, benzyl bromide, 2-Bromo-1-(4-methylphenyl)-1-propanone, 1-Fluoro-2-nitrobenzene, allyl iodide, ethyl chloroacetate, 4-bromobutyl acetate, 1-bromobutane, sodium azide, tert-butyl alcohol, 230 mesh copper (Sigma-Aldrich); 2-methylquinoline, the pre-coated silica gel 60 F254 TLC plates, syringe filters with 20 nm pores (Merck); silica-gel 40–60 µm, 60 Å, phenylacetylene, 4-bromobenzyl bromide, 4-chlorobenzyl bromide, 4-iodobenzyl bromide, 1-(bromomethyl)-2,3-dichlorobenzene, 8-chloro-2-methylquinoline, ethyl acetate Ph. Eur, dichloromethane Ph. Eur., hexane Ph. Eur. (Acros Organics); 2-methyl-4(3H)-quinazolinone (Infinite Fine Chemicals). Water was purified by simple distillation.

### Syntheses

Reactions were carried out in 12 cm^3^ septum-sealed vials. If not otherwise specified, the reactions were prepared by mixing the reagents in the following order: copper powder (1 mol%), organic azide precursor (1 mmol of benzyl bromides, 0.50 mmol of quinoline and quinazolinone analogues), sodium azide (1 eq. respectively to a precursor) and 2 cm^3^ of water. Optionally, 10% (v/v) of tert-butyl alcohol was added. Finally, terminal alkyne (1 eq. respectively to a precursor) was introduced either at RT and then the reaction mixture was heated to 85 °C, which resembled the batch approach (conditions A) or dropwise through a septum at the point at which the mixture reached 85 °C (conditions B). The progression of the reactions was monitored using TLC. Then 50 cm^3^ of CH_2_Cl_2,_ was added to a reaction mixture and the organic phase was washed with 50 cm^3^ of water, dried over anhydrous MgSO_4_ and evaporated under reduced pressure to yield a triazole. Alternatively, the crude solid was filtered and washed on a funnel with water and then with 5 cm^3^ of diethyl ether. NMR samples were prepared from the crude residue to measure the conversion. Recrystallization was employed to turn some viscous triazoles into a solid form if necessary. The yields of isolated triazoles are given.

#### 1-benzyl-4-phenyl-1H-1,2,3-triazole (1a)

Synthesized according to conditions B of the general procedure. Benzyl bromide (1 mmol, 171 mg), phenylacetylene (1 mmol, 102 mg), sodium azide (1 mmol, 65 mg). After 2 h the first work-up protocol was executed. 229 mg (97%) of green solid was obtained; mp: 123–125 °C (lit.^33^ 126–127 °C); ^1^H NMR (400 MHz; DMSO-d_6_; TMS): δ 8.65 (s,1H, CH), 7.85–7.92 (m, 2H, Ar), 7.28–7.48 (m, 8H, Ar), 5.67 (s, 2H, ArCH_2_N); ^13^C NMR (101 MHz; DMSO-d_6_; TMS): δ 147.2, 136.5, 131.17, 129.3, 129.2, 128.6, 128.4, 125.7, 122.0, 53.5.

#### 1-[(4-bromophenyl)methyl]-4-phenyl-1H-1,2,3-triazole (2a)

Synthesized according to conditions B of the general procedure. 4-Bromobenzyl bromide (1 mmol, 250 mg), phenylacetylene (1 mmol, 102 mg), sodium azide (1 mmol, 65 mg). After 2 h the first work-up protocol was executed. 312 mg (99%) of light green solid was obtained; mp: 154–156 °C, (lit.^34^ 156–158 °C); ^1^H NMR(400 MHz; DMSO-d_6_; TMS): δ 8.64 (1H, s, CH), 7.87 (d, *J* = 7.7 Hz, 2H, Ar), 7.59 (d, *J* = *8.1* Hz, 2H, Ar), 7.44 (d, *J* = 7.6 Hz, 2H, Ar), 7.33 (d, *J* = 8.0Hz, 1H, Ar), 5.66 (s, 2H, ArCH_2_N); ^13^C NMR (101 MHz; DMSO-d_6_; TMS): δ 147.2, 135.8, 132.2, 131.1, 130.6, 129.3, 128.4, 125.6, 122.1, 121.9, 52.8.

#### 1-[(4-chlorophenyl)methyl]-4-phenyl-1H-1,2,3-triazole (3a)

Synthesized according to conditions B of the general procedure. 4-Chlorobenzyl bromide (1 mmol, 206 mg), phenylacetylene (1 mmol, 102 mg), sodium azide (1 mmol, 65 mg); After 3 h the first work-up protocol was executed. 268 mg (99%) of light yellow-green solid was obtained; mp: 143–144 °C (lit.^35^ 142–145 °C); ^1^H NMR(400 MHz; DMSO-d_6_; TMS): δ 8.65 (s, 1H, CH), 7.88 (d, *J* = 7.7 Hz, 2H, Ar), 7.28–7.48 (m, 7H, Ar), 5.68 (s, 2H, ArCH_2_N); ^13^C NMR (101 MHz; DMSO-d_6_; TMS): δ 147.2, 135.4, 133.4, 131.1, 130.3, 129.4, 129.3, 128.4, 125.7, 122.1, 52.8.

#### 1-[(4-iodophenyl)methyl]-4-phenyl-1H-1,2,3-triazole (4a)

Synthesized according to conditions B of the general procedure. 4-Iodobenzyl bromide (1 mmol, 297 mg), phenylacetylene (1 mmol, 102 mg), sodium azide (1 mmol, 65 mg), t-BuOH (0.2 cm^3^, 10% v/v). After 2 h the first work-up protocol was executed. 366 mg (99%) of yellow solid was obtained; mp: 139–141 °C (lit.^36^ 154–156 °C); ^1^H NMR (400 MHz; DMSO-d_6_; TMS): δ 8.63 (s, 1H, CH), 7.87 (d, *J* = 7.6 Hz, 2H, Ar), 7.76 (d, *J* = 7.9 Hz, 2H, Ar), 7.44 (d, *J* = 7.7 Hz, 2H, Ar), 7.32 (t, *J* = 7.5 Hz, 1H, Ar), 7.17 (d, *J* = 7.9 Hz, 2H, Ar), 5.63 (s, 2H, ArCH_2_N); ^13^C NMR (101 MHz; DMSO-d_6_; TMS): δ 147.2, 138.0, 136.2, 131.1, 130.7, 129.3, 128.4, 125.7, 122.0, 94.9, 53.0.

#### 1-[(2,3-dichlorophenyl)methyl]-4-phenyl-1H-1,2,3-triazole (5a, C_15_H_11_Cl_2_N_3_)

Synthesized according to conditions B of the general procedure. 1-(Bromomethyl)-2,3-dichlorobenzene (1 mmol, 240 mg), phenylacetylene (1 mmol, 102 mg), sodium azide (1 mmol, 65 mg). After 2 h the first work-up protocol was executed. 282 mg (93%) of beige solid was obtained; mp: 119–120 °C; ^1^H NMR (400 MHz; DMSO-d_6_; TMS): δ 8.64 (s, 1H, CH), 7.88 (d, *J* = 7.9 Hz, 2H, Ar), 7.66 (dd, *J* = 8.1 Hz, 1.2 Hz, 1H, Ar), 7.37–7.48 (m, 3H, Ar), 7.33 (appt, *J* = 7.4 Hz, 1H, Ar), 7.24 (d, *J* = 7.7 Hz, 1H, Ar), 5.82 (s, 2H, ArCH_2_N); ^13^C NMR (101 MHz; DMSO-d_6_; TMS): δ 147.0, 136.2, 132.7, 131.3, 131.0, 129.5, 129.3, 129.1, 128.4, 125.7, 122.5, 51.8; HRMS (m/z): [M + H]^+^ calcd. for C_15_H_11_^35^Cl_2_N_3_, 304.0403; found, 304.0409.

#### 1-benzyl-4-butyl-1H-1,2,3-triazole (1b)

Synthesized according to conditions B of the general procedure. Benzyl bromide (1 mmol, 171 mg), 1-hexyne (1 mmol, 82 mg), sodium azide (1 mmol, 65 mg), t-BuOH (0.2 cm^3^, 10% v/v). After 3 h the first work-up protocol was executed. 193 mg (90%) of green resin was obtained (lit.^37^ 61–62 °C); ^1^H NMR (400 MHz; DMSO-d_6_; TMS): δ 7.89 (s, 1H, CH), 7.24–7.41 (m, 5H, Ar), 5.53 (s, 2H, ArCH_2_N), 2.60 (t, *J* = 7.6 Hz, 2H, CH_2_CH_2_CH_2_CH_3_), 1.56 (tt, *J* = 7.6 Hz, 2H, CH_2_CH_2_CH_2_CH_3_), 1.31 (tq, *J* = 7.3 Hz, 2H, CH_2_CH_2_CH_2_CH_3_), 0.89 (t, *J* = 7.3 Hz, 3H, CH_2_CH_2_CH_2_CH_3_). ^13^C NMR (101 MHz; DMSO-d_6_; TMS): δ 147.7, 136.7, 129.2, 128.4, 128.2, 122.3, 53.1, 31.6, 25.1, 22.1, 14.1.

#### 1-[(4-bromophenyl)methyl]-4-butyl-1H-1,2,3-triazole (2b)

Synthesized according to conditions A of the general procedure. 4-Bromobenzyl bromide (1 mmol, 250 mg), 1-hexyne (1 mmol, 82 mg), sodium azide (1 mmol, 65 mg). After 1 h the first work-up protocol was executed. 291 mg (99%) of green solid was obtained; mp: 64–66 °C (lit.^38^ 64–65 °C); ^1^H NMR (400 MHz; DMSO-d_6_; TMS): δ 7.90 (s, 1H, CH), 7.55 (d, *J* = 8.1 Hz, 2H, Ar), 7.25 (d, *J* = 8.1 Hz, 2H, Ar), 5.54 (s, 2H, ArCH_2_N), 2.60 (t, *J* = 7.6 Hz, 2H, CH_2_CH_2_CH_2_CH_3_), 1.55 (tt, *J* = 7.5 Hz, 2H, CH_2_CH_2_CH_2_CH_3_), 1.30 (tq, *J* = 7.4 Hz, 2H, CH_2_CH_2_CH_2_CH_3_), 0.87 (t, *J* = 7.4 Hz, 3H, CH_2_CH_2_CH_2_CH_3_); ^13^C NMR (101 MHz; DMSO-d_6_; TMS): δ 147.8, 136.1, 132.1, 130.5, 122.4, 121.8, 52.4, 31.5, 25.2, 22.2, 14.1.

#### 4-butyl-1-[(4-chlorophenyl)methyl]-1H-1,2,3-triazole (3b)

Synthesized according to conditions A of the general procedure. 4-Chlorobenzyl bromide (1 mmol, 206 mg), 1-hexyne (1 mmol, 82 mg), sodium azide (1 mmol, 65 mg). After 1 h the first work-up protocol was executed. 191 mg (72%) of light green solid was obtained; mp: 58–60 °C (lit.^39^ 52–54 °C); ^1^H NMR (400 MHz; DMSO-d_6_; TMS): δ 7.89 (s, 1H, CH), 7.41–7.46 (2H, m, Ar), 7.28–7.33 (m, 2H, Ar), 5.54 (s, 2H, ArCH_2_N), 2.60 (t, *J* = 7.6 Hz, 2H, CH_2_CH_2_CH_2_CH_3_), 1.55 (tt, *J* = 7.5 Hz, 2H, CH_2_CH_2_CH_2_CH_3_), 1.31 (tq, *J* = 7.4 Hz, 2H, CH_2_CH_2_CH_2_CH_3_), 0.88 (t, *J* = 7.4 Hz, 3H, CH_2_CH_2_CH_2_CH_3_); ^13^C NMR (101 MHz; DMSO-d_6_; TMS): δ 147.8, 135.7, 133.2, 130.2, 129.1, 122.4, 52.3, 31.5, 25.2, 22.2, 14.1.

#### 4-butyl-1-[(4-iodophenyl)methyl]-1H-1,2,3-triazole (4b, C_13_H_16_IN_3_)

Synthesized according to conditions B of the general procedure. 4-Iodobenzyl bromide (1 mmol, 297 mg), 1-hexyne (1 mmol, 82 mg), sodium azide (1 mmol, 65 mg), t-BuOH (0.2 cm^3^, 10% v/v);. After 2 h the first work-up protocol was executed. 341 mg (99%) of beige solid was obtained; mp: 94–95 °C; ^1^H NMR (400 MHz; DMSO-d_6_; TMS): δ 7.88 (1H, s, CH), 7.71–7.77 (m, 2H, Ar), 7.06–7.11 (m, 2H, Ar), 5.50 (s, 2H, ArCH_2_N), 2.60 (t, *J* = 7.6 Hz, 2H, CH_2_CH_2_CH_2_CH_3_), 1.55 (tt, *J* = 7.6 Hz, 2H, CH_2_CH_2_CH_2_CH_3_), 1.31 (tq, *J* = 7.4 Hz, 2H, CH_2_CH_2_CH_2_CH_3_), 0.88 (t, *J* = 7.4 Hz, 3H, CH_2_CH_2_CH_2_CH_3_); ^13^C NMR (101 MHz; DMSO-d_6_; TMS): δ 147.8, 138.0, 136.5, 130.6, 122.4, 94.7, 52.6, 31.6, 25.2, 22.2, 14.1; HRMS (m/z): [M + H]^+^ calcd. for C_13_H_16_IN_3_, 342.0462; found, 342.0464.

#### 4-butyl-1-[(2,3-dichlorophenyl)methyl]-1H-1,2,3-triazole (5b, C_13_H_15_Cl_2_N_3_)

Synthesized according to conditions B of the general procedure. 1-(Bromomethyl)-2,3-dichlorobenzene (1 mmol, 240 mg), 1-hexyne (1 mmol, 82 mg), sodium azide (1 mmol, 65 mg). After 2 h the first work-up protocol was executed. 281 mg (99%) of white crystals was obtained; mp: 79–80 °C; ^1^H NMR (400 MHz; DMSO-d_6_; TMS): δ 7.91 (s, 1H, CH), 7.64 (dd, *J* = 8.1 Hz, 1.2 Hz, 1H, Ar), 7.38 (appt, *J* = 7.9 Hz, 1H, Ar), 7.09 (dd, *J* = 7.7 Hz, 1.1 Hz, 1H, Ar), 5.70 (s, 2H, ArCH_2_N), 2.62 (t, *J* = 7.6 Hz, 2H, CH_2_CH_2_CH_2_CH_3_), 1.57 (appquint, *J* = 7.5 Hz, 2H, CH_2_CH_2_CH_2_CH_3_), 1.31 (appsextet, *J* = 7.4 Hz, 2H, CH_2_CH_2_CH_2_CH_3_), 0.88 (t, *J* = 7.4 Hz, 3H, CH_2_CH_2_CH_2_CH_3_); ^13^C NMR (101 MHz; DMSO-d_6_; TMS): δ 147.6, 136.7, 132.6, 131.0, 130.8, 129.1, 129.0, 122.9, 51.4, 31.5, 25.1, 22.1, 14.1; HRMS (m/z): [M + H]^+^ calcd. for C_13_H_15_^35^Cl_2_N_3_, 284.0716; found, 284.0718.

#### (1-benzyl-1H-1,2,3-triazol-4-yl)methanol (1c)

Synthesized according to conditions A of the general procedure. Benzyl bromide (1 mmol, 171 mg), propargyl alcohol (1 mmol, 56 mg), sodium azide (1 mmol, 65 mg); After 2 h the first work-up protocol was executed. 182 mg (96%) of beige solid was obtained; mp: 77–79 °C (lit.^40^ 77–78 °C); ^1^H NMR (400 MHz; DMSO-d_6_; TMS): δ 8.01 (s, 1H, CH), 7.28–7.40 (m, 5H, Ar), 5.57 (s, 2H, ArCH_2_N), 5.16 (t, *J* = 5.7 Hz, 1H, OH), 4.51 (d, *J* = 5.5 Hz, 2H, CH_2_OH); ^13^C NMR (101 MHz; DMSO-d_6_; TMS): δ 148.8, 136.6, 129.2, 128.6, 128.4, 123.3, 55.6, 53.2.

#### {1-[(4-bromophenyl)methyl]-1H-1,2,3-triazol-4-yl}methanol (2c)

Synthesized according to conditions A of the general procedure. 4-Bromobenzyl bromide (1 mmol, 250 mg), propargyl alcohol (1 mmol, 56 mg), sodium azide (1 mmol, 65 mg); After 1 h the first work-up protocol was executed. 252 mg (94%) of pale beige solid was obtained; mp: 110–111 °C; ^1^H NMR (400 MHz; DMSO-d_6_; TMS): δ 8.02 (s, 1H, CH), 7.58 (d, *J* = 8.0 Hz, 2H, Ar), 7.27 (d, *J* = 8.0 Hz, 2H, Ar), 5.56 (s, 2H, ArCH_2_N), 5.16 (t, *J* = 5.6 Hz, 1H, OH), 4.50 (d, *J* = 5.6 Hz, 2H, CH_2_OH); ^13^C NMR (101 MHz; DMSO-d_6_; TMS): δ 148.9, 136.0; 132.1; 130.6; 121.9, 123.4, 55.6, 52.5.

#### {1-[(4-chlorophenyl)methyl]-1H-1,2,3-triazol-4-yl}methanol (3c, C_10_H_10_ClN_3_O)

Synthesized according to conditions A of the general procedure. 4-Chlorobenzyl bromide (1 mmol, 206 mg), propargyl alcohol (1 mmol, 56 mg), sodium azide (1 mmol, 65 mg). After 1 h the first work-up protocol was executed. 215 mg (96%) of pale yellow solid was obtained; mp: 91–93 °C; ^1^H NMR (400 MHz; DMSO-d_6_; TMS): δ 8.05 (s, 1H, CH), 7.40–7.47 (m, 2H, Ar), 7.32–7.38 (m, 2H, Ar), 5.60 (s, 2H, ArCH_2_N), 5.22 (t, *J* = 5.7 Hz, 1H, OH), 4.55 (d, *J* = 5.7 Hz, 2H, CH_2_OH); ^13^C NMR (101 MHz; DMSO-d_6_; TMS): δ 148.9, 135.6, 133.3, 130.3, 129.2, 123.4, 55.5, 52.4; HRMS (m/z): [M + H]^+^ calcd. for C_10_H_10_^35^ClN_3_O, 224.0585; found, 224.0589.

#### {1-[(4-iodophenyl)methyl]-1H-1,2,3-triazol-4-yl}methanol (4c, C_10_H_10_IN_3_O)

Synthesized according to conditions A of the general procedure. 4-Iodobenzyl bromide (1 mmol, 297 mg), propargyl alcohol (1 mmol, 56 mg), sodium azide (1 mmol, 65 mg), t-BuOH (0.2 cm^3^, 10% v/v). After 2 h the first work-up protocol was executed. 314 mg (99%) of white solid was obtained; mp: 139–140 °C; ^1^H NMR (400 MHz; DMSO-d_6_; TMS): δ 8.02 (s, 1H, CH), 7.74 (d, *J* = 8.0 Hz, 2H, Ar), 7.12 (d, *J* = 8.0 Hz, 2H, Ar), 5.55 (s, 2H, ArCH_2_N), 5.19 (t, *J* = 5.3 Hz, 1H, OH), 4.53 (d, *J* = 5.7 Hz, 2H, CH_2_OH); ^13^C NMR (101 MHz; DMSO-d_6_; TMS): δ 148.9, 138.0, 136.4, 130.7, 123.4, 94.8, 55.5, 52.6; HRMS (m/z): [M + H]^+^ calcd. for C_10_H_10_IN_3_O, 315.9941; found, 315.9944.

#### {1-[(2,3-dichlorophenyl)methyl]-1H-1,2,3-triazol-4-yl}methanol (5c, C_10_H_9_Cl_2_N_3_)

Synthesized according to conditions B of the general procedure. 1-(Bromomethyl)-2,3-dichlorobenzene (1 mmol, 240 mg), propargyl alcohol (1 mmol, 56 mg), sodium azide (1 mmol, 65 mg). After 2 h the first work-up protocol was executed. 256 mg (99%) of off-white solid was obtained; mp: 111–112 °C; ^1^H NMR (400 MHz; DMSO-d_6_; TMS): δ 8.03 (s, 1H, CH), 7.65 (dd, *J* = 8.1 Hz, 1.2 Hz, 1H, Ar), 7.39 (appt, *J* = 7.9 Hz, 1H, Ar), 7.14 (dd, *J* = 7.7 Hz, 1.2 Hz, 1H, Ar), 5.75 (s, 2H, ArCH_2_N), 5.21 (t, *J* = 5.6 Hz, 1H, OH), 4.54 (d, *J* = 5.7 Hz, 2H, CH_2_OH); ^13^C NMR (101 MHz; DMSO-d_6_; TMS): δ 148.8, 136.6, 132.7, 131.1, 130.9, 129.3, 129.0, 123.8, 55.5, 51.5; HRMS (m/z): [M + H]^+^ calcd. for C_10_H_9_^35^Cl_2_N_3_, 258.0195; found, 258.0200.

#### 1-butyl-4-phenyl-1H-1,2,3-triazole (6a)

n-Bromobutane (1 mmol, 137 mg), phenylacetylene (1 mmol, 102 mg), sodium azide (1 mmol, 65 mg) was stirred for 24 h and the first work-up protocol was executed. 190 mg (94%) of off-white solid was obtained; mp: 46.0–47.5 °C (lit.^41^ 46–47 °C); ^1^H NMR (500 MHz, CDCl_3_, TMS): δ 7.83 (d, J = 7.2 Hz, 2H, Ar), 7.74 (s, 1H, CH), 7.41 (t, J = 7.6 Hz, 2H, Ar), 7.32 (t, J = 7.5 Hz, 1H, Ar), 4.39 (t, J = 7.2 Hz, 2H, NCH_2_CH_2_CH_2_CH_3_), 1.96–1.88(m, 2H, CH_2_CH_2_CH_2_CH_3_), 1.43–1.34 (m, 2H, CH_2_CH_2_CH_2_CH_3_), 0.97 (t, J = 7.4 Hz, 3H, CH_2_CH_2_CH_2_CH_3_); ^13^C NMR (126 MHz, CDCl_3_, TMS): δ 147.6, 130.8, 128.8, 128.0, 125.6, 119.7, 50.0, 32.2, 19.7, 13.4.

#### 4-phenyl-1-(prop-2-en-1-yl)-1H-1,2,3-triazole (7a)

3-Iodopropene (1 mmol, 168 mg), phenylacetylene (1 mmol, 102 mg), sodium azide (1 mmol, 65 mg) was stirred for 1 h and the first work-up protocol was executed. 185 mg (99%) of brownish-yellow solid was obtained; mp: 54.0–56.0 °C (lit.^41^ 56–57 °C); ^1^H NMR (500 MHz, CDCl_3_, TMS): δ 7.84–7.81 (m, 2H, Ar), 7.76 (s, 1H, CH), 7.44–7.40 (m, 2H, Ar), 7.35–7.31 (m, 1H, Ar), 6.10–6.02 (m, 2H, CH_2_CH = CH_2_), 5.40–5.32 (m, 1H, CH_2_CH = CH_2_), 5.02 (appdt, J = 6.2 Hz, 1.4 Hz, 2H, CH_2_CH = CH_2_)_;_^13^C NMR (126 MHz, CDCl_3_, TMS): δ 147.8, 131.3, 130.6, 128.8, 128.1, 125.6, 120.0, 119.8, 52.6.

#### ethyl (4-phenyl-1H-1,2,3-triazol-1-yl)acetate (8a)

Ethyl chloroacetate (1 mmol, 123 mg), phenylacetylene (1 mmol, 102 mg), sodium azide (1 mmol, 65 mg), t-BuOH (0,2 cm^3^, 10% v/v) was stirred for 24 h at room temperature then 24 h at 85 °C and the first work-up protocol was executed. 211 mg (91%) of white crystals was obtained; mp: 90.5–93.0 °C (lit.^41^ 94–95 °C); ^1^H NMR (500 MHz, CDCl_3_, TMS): δ 7.91 (s, 1H, CH), 7.87–7.83 (m, 2H, Ar) 7.45–7.41 (m, 2H, Ar), 7.37–7.32 (m, 1H, Ar), 5.21 (s, 2H, NCH_2_CO), 4.29 (q, J = 7.1 Hz, 2H, OCH_2_CH_3_), 1.32 (t, J = 7.1 Hz, 3H, OCH_2_CH_3_)_;_^13^C NMR (126 MHz, CDCl_3_, TMS): δ 166.4, 148.0, 130.4, 128.8, 128.2, 125.8, 121.4, 62.4, 50.9, 14.0.

#### 4-(4-phenyl-1H-1,2,3-triazol-1-yl)butyl acetate (9a)

4-Bromobutyl acetate (1 mmol, 195 mg), phenylacetylene (1 mmol, 102 mg), sodium azide (1 mmol, 65 mg) was stirred for 24 h at 85 °C and the first work-up protocol was executed. 255 mg (98%) of off-white solid was obtained; mp: 57.0–60.0 °C; ^1^H NMR (500 MHz, CDCl_3_, TMS): δ 7.84–7.81 (m, 2H, Ar), 7.76 (s, 1H, CH), 7.44–7.40 (m, 2H, Ar), 7.35–7.31 (m, 1H, Ar), 4.44 (t, J = 7.2 Hz, 2H, NCH_2_CH_2_CH_2_CH_2_), 4.11 (t, J = 6.4 Hz, 2H, CH_2_CH_2_CH_2_CH_2_O), 2.09–1.98 (m, 2H, CH_2_CH_2_CH_2_CH_2_O) 2.05 (s, 3H, OCCH_3_), 1.74–1.67 (m, 2H, CH_2_CH_2_CH_2_CH_2_O)_;_^13^C-NMR (126 MHz, CDCl_3_, TMS): δ 170.9, 147.6, 130.6, 128.8, 128.0, 125.6, 119.9, 63.3, 49.7, 26.9, 25.6, 20.8.

#### 1-(4-methylphenyl)-2-(4-phenyl-1H-1,2,3-triazol-1-yl)propan-1-one (10a)

2-bromo-1-(4-methylphenyl)propan-1-one (0.50 mmol, 114 mg), phenylacetylene (0.50 mmol, 51 mg), sodium azide (0.50 mmol, 65 mg), t-BuOH (0.2 cm^3^, 10% v/v) was stirred for 48 h at room temperature. Colored resin solidified in a fridge at −15 °C. Solids was stirred with 2 cm^3^ of Et_2_O. Ether was removed. 135 mg (94%) of white solid mp: 101–101.5 °C, ^1^H NMR (500 MHz; DMSO-d_6_; TMS): δ 8.79 (s, 1H), 8.01–7.99 (m, 2H), 7.88 (dd, *J* = 8.3, 1.2, 2H), 7.47–7.44 (m, 2H), 7.38 (d, *J* = 7.9, 2H), 7.36–7.32 (m, 1H), 6.73 (q, *J* = 7.2, 1H), 2.38 (s, 3H), 1.81 (d, *J* = 7.2, 3H).

^13^C NMR (126 MHz; DMSO-d_6_; TMS): δ 194.9, 146.7, 145.3, 131.7, 131.1, 130.1, 129.36, 129.30, 128.3, 125.6, 121.8, 60.1, 21.7, 18.2.

#### 2-[4-(hydroxymethyl)-1H-1,2,3-triazol-1-yl]-1-(4-methylphenyl)propan-1-one (10b)

2-bromo-1-(4-methylphenyl)propan-1-one (0.50 mmol, 114 mg), phenylacetylene (0.50 mmol, 51 mg), sodium azide (0.50 mmol, 65 mg), t-BuOH (0.2 cm^3^, 10% v/v) was stirred for 48 h at room temperature. Colored resin solidified in a fridge at −15 °C. Solids was stirred with 2 cm^3^ of Et_2_O. Ether was removed. 116 mg (96%) of white solid mp: 122–123 °C, ^1^H NMR (500 MHz; DMSO-d_6_; TMS): δ 8.13 (s, 1H), 7.96–7.94 (m, 2H), 7.38–7.36 (m, 2H), 6.63 (q, *J* = 7.2, 1H), 5.22 (s, 1H), 4.53 (s, 2H), 2.38 (s, 3H), 1.72 (d, *J* = 7.2, 3H), ^13^C NMR (126 MHz; DMSO): δ 194.8, 148.4, 145.2, 131.8, 130.0, 129.2, 123.0, 59.6, 55.5, 21.7, 18.2.

#### 1-(4-nitrophenyl)-4-phenyl-1H-1,2,3-triazole (11a)

Synthesized according to conditions A of the general procedure. 1-fluoro-2-nitrobenzene (1 mmol, 141 mg), phenylacetylene (1 mmol, 102 mg), sodium azide (1 mmol, 65 mg). After 24 h a NMR sample was prepared. Product was not isolated.

#### [1-(4-nitrophenyl)-1H-1,2,3-triazol-4-yl]methanol (11b)

Synthesized according to conditions A of the general procedure. 1-fluoro-2-nitrobenzene (1 mmol, 141 mg), propargyl alcohol (1 mmol, 56 mg), sodium azide (1 mmol, 65 mg). After 24 h a NMR sample was prepared. Product was not isolated.

#### 2-[(4-phenyl-1H-1,2,3-triazol-1-yl)methyl]quinoline (12a)

Synthesized according to conditions B of the general procedure. 2-(Bromomethyl)-quinoline (0.50 mmol, 111 mg), phenylacetylene (0.55 mmol, 56 mg), sodium azide (0.50 mmol, 33 mg). After 30 min the first work-up protocol was executed. 138 mg (99%) of beige solid was obtained; mp: 160 °C (lit.^23^ 160–162 °C); ^1^H NMR (400 MHz; DMSO-d_6_; TMS): δ 8.74 (s, 1H, Ar), 8.42 (d, *J* = 8.5 Hz, 1H, Ar), 8.00 (dd, *J* = 8.5 Hz, 4.1 Hz, 2H, Ar), 7.88 (d, *J* = 7.7 Hz, 2H, Ar), 7.79 (appt, *J* = 7.8 Hz, 1H, Ar), 7.63 (appt, *J* = 7.5 Hz, 1H, Ar), 7.45 (appt, *J* = 7.7 Hz, 3H, Ar), 7.34 (appt, *J* = 7.4 Hz, 1H, Ar), 5.97 (2H, s, ArCH_2_N); ^13^C NMR (101 MHz; DMSO-d_6_; TMS): δ 154.7, 148.4, 147.7, 137.8, 130.5, 130.3, 129.2, 128.8, 128.2, 127.8, 127.6, 127.2, 125.7, 120.2, 119.7, 56.5.

#### 2-[(4-butyl-1H-1,2,3-triazol-1-yl)methyl]quinoline (12b)

Synthesized according to conditions B of the general procedure. 2-(Bromomethyl)-quinoline (0.50 mmol, 111 mg), 1-hexyne (0.55 mmol, 45 mg), sodium azide (0.50 mmol, 33 mg). After 30 min the first work-up protocol was executed. 107 mg (85%) of beige solid was obtained; mp: 49–50 °C; ^1^H NMR (400 MHz; DMSO-d_6_; TMS): δ 8.39 (d, *J* = 8.6 Hz, 1H, Ar), 8.01–7.95 (m, 3H, Ar), 7.82–7.76 (m, 1H, Ar), 7.63 (appt, *J* = 8.2 Hz, 1H, Ar), 7.32 (d, *J* = 8.5 Hz, 1H, Ar), 5.86 (s, 2H, ArCH_2_N), 2.64 (t, *J* = 7.6 Hz, 2H, CH_2_CH_2_CH_2_CH_3_), 1.59 (appquint, *J* = 7.6 Hz, 2H, CH_2_CH_2_CH_2_CH_3_), 1.33 (appsextet, *J* = 7.4 Hz, 2H, CH_2_CH_2_CH_2_CH_3_), 0.89 (t, *J* = 7.3 Hz, 3H, CH_2_CH_2_CH_2_CH_3_). ^13^C NMR (101 MHz; DMSO-d_6_; TMS): δ 156.3, 147.6, 137.8, 130.5, 129.1, 128.4, 127.5, 127.2, 123.2, 120.2, 55.4, 31.6, 25.1, 22.1, 14.1;

#### {1-[(quinolin-2-yl)methyl]-1H-1,2,3-triazol-4-yl}methanol (12c)

Synthesized according to conditions B of the general procedure. 2-(Bromomethyl)-quinoline (0.50 mmol, 111 mg) propargyl alcohol (0.55 mmol, 31 mg), sodium azide (0.50 mmol, 33 mg). After 30 min the first work-up protocol was executed. 106 mg (88%) of white solid was obtained; mp: 110 °C (lit.^23^ 109–111 °C); ^1^H NMR (400 MHz; DMSO-d_6_; TMS): δ 8.41 (d, *J* = 8.5 Hz, 1H, Ar), 8.13 (s, 1H), 7.99 (d, *J* = 8.7 Hz, 2H, Ar), 7.79 (appt, *J* = 7.8 Hz, 1H, Ar), 7.63 (appt, *J* = 7.7 Hz, 1H, Ar), 7.36 (d, *J* = 8.5 Hz, 1H, Ar), 5.90 (s, 2H, ArCH_2_N), 5.19 (t, *J* = 5.7 Hz, 1H, OH), 4.55 (d, *J* = 5.6 Hz, 2H, CH_2_OH); ^13^C NMR (101 MHz; DMSO-d_6_; TMS): δ 156.3, 148.8, 147.4, 138.0, 130.6, 129.1, 128.4, 127.5, 127.3, 124.2, 120.3, 55.5, 55.4.

#### 8-chloro-2-[(4-phenyl-1H-1,2,3-triazol-1-yl)methyl]quinoline (13a, C_18_H_13_ClN_4_)

Synthesized according to conditions B of the general procedure. 2-(Bromomethyl)-8-chloroquinoline (0.50 mmol, 128 mg), phenylacetylene (0.50 mmol, 51 mg), sodium azide (0.50 mmol, 33 mg). After 6 min the first work-up protocol was executed and the residue was recrystallized from EtOH-MTBE, 80 mg (50%) of a white crystalline solid was obtained; mp: 131–132 °C; ^1^H NMR (400 MHz; DMSO-d_6_; TMS): δ 8.78 (s, 1H, CH), 8.51 (d, *J* = 8.5 Hz, 1H, Ar), 8.00 (d, *J* = 7.6 Hz, 1H, Ar), 7.99 (d, *J* = 7.6 Hz, 1H, Ar) 7.89 (d, *J* = 7.3 Hz, 1H, Ar), 7.89 (d, *J* = 7.3 Hz, 1H, Ar) 7.61 (s, 1H, Ar), 7.49 (d, *J* = 8.6 Hz, 1H, Ar), 7.46 (appt, *J* = 7.7 Hz, 2H, Ar), 7.35 (appt, *J* = 7.4 Hz, 1H, Ar), 6.04 (s, 2H, ArCH_2_N); ^13^C NMR (101 MHz; DMSO-d_6_; TMS): δ 156.9, 147.1, 143.3, 138.8, 132.5, 131.2, 130.6, 129.4, 129.1, 128.4, 127.9, 127.5, 125.7, 123.1, 121.1, 55.7; HRMS (m/z): [M-H]¯ calcd. for C_18_H_13_^35^ClN_4_, 319.0756; found, 319.0755.

#### 2-[(4-butyl-1H-1,2,3-triazol-1-yl)methyl]-8-chloroquinoline (13b, C_16_H_17_ClN_4_)

Synthesized according to conditions A of the general procedure. 2-(Bromomethyl)-8-chloroquinoline (0.50 mmol, 128 mg), 1-hexyne (0.50 mmol, 41 mg), sodium azide (0.50 mmol, 33 mg), t-BuOH (0.2 cm^3^, 10% v/v); After 24 h the first work-up protocol was executed and after recrystallization from MTBE-EtOH, 101 mg (65%) of a pale yellow solid was obtained; mp: 65–67 °C; ^1^H NMR (400 MHz; DMSO-d_6_; TMS): δ 8.48 (dd, *J* = 8.5 Hz, 0.8 Hz, 1H, Ar), 8.05 (d, *J* = 0.8 Hz, 1H, CH), 8.00–7.96 (m, 2H, Ar), 7.60 (apptd, *J* = 7.9 Hz, 0.9 Hz, 1H, Ar), 7.38 (dd, *J* = 8.5 Hz, 1.0 Hz, 1H, Ar), 5.93 (s, 2H, ArCH_2_N), 2.65 (t, *J* = 7.5 Hz, 2H, CH_2_CH_2_CH_2_CH_3_), 1.6 (appquint, *J* = 7.5 Hz, 2H, CH_2_CH_2_CH_2_CH_3_), 1.32 (appsextet, *J* = 7.5 Hz, 2H, CH_2_CH_2_CH_2_CH_3_), 0.88 (apptd, *J* = 7.4 Hz, 0.8 Hz, 3 H, CH_2_CH_2_CH_2_CH_3_). ^13^C NMR (101 MHz; DMSO-d_6_; TMS): δ 157.2, 147.7, 143.2, 138.6, 132.5, 130.6, 129.0, 127.9, 127.5, 123.5, 121.0, 55.3, 31.6, 25.1, 22.1, 14.1; HRMS (m/z): [M-H]¯ calcd. for C_16_H_17_^35^ClN_4_, 299.1069; found, 299.1067.

#### {1-[(8-chloroquinolin-2-yl)methyl]-1H-1,2,3-triazol-4-yl}methanol (13c, C_13_H_11_ClN_4_)

Synthesized according to conditions B of the general procedure 2-(Bromomethyl)-8-chloroquinoline (0.50 mmol, 128 mg), propargyl alcohol (0.50 mmol, 28 mg), sodium azide (0.50 mmol, 33 mg). Reaction was carried for 15 min. After recrystallization from EtOH-MTBE 90 mg (65%) of an off-white solid was obtained; mp: 116–119 °C; ^1^H NMR (400 MHz; DMSO-d_6_; TMS): δ 8.49 (d, *J* = 8.5 Hz, 1H, Ar), 8.17 (s, 1H, CH), 7.99 (d, *J* = 8.0 Hz, 1H, Ar), 7.98 (d, *J* = 7.6 Hz, 1H, Ar) 7.62 (d, *J* = 7.8 Hz, 1H, Ar), 7.39 (d, *J* = 8.5 Hz, 1H, Ar), 5.96 (s, 2H, ArCH_2_N), 5.21 (t, *J* = 5.7 Hz, 1H, OH), 4.56 (d, *J* = 5.6 Hz, 2H, CH_2_OH). ^13^C NMR (101 MHz; DMSO-d_6_; TMS): δ 157.2, 148.9, 143.3, 138.7, 132.5, 130.6, 129.0, 127.9, 127.5, 124.3, 121.1, 55.6, 55.4; HRMS (m/z): [M-H]¯ calcd. for C_13_H_11_^35^ClN_4_, 273.0549; found, 273.0547.

#### 1-{1-[(8-chloroquinolin-2-yl)methyl]-1H-1,2,3-triazol-4-yl}ethan-1-ol (13d, C_14_H_13_ClN_4_)

Synthesized according to conditions B of the general procedure. 2-(Bromomethyl)-8-chloroquinoline (0.50 mmol, 128 mg), but-3-yn-2-ol (0.50 mmol, 35 mg), sodium azide (0.50 mmol, 33 mg). After 20 min the first work-up protocol was executed and after recrystallization from EtOH-MTBE 72 mg (50%) of a beige solid was obtained; mp: 141–142 °C. ^1^H NMR (400 MHz; DMSO-d_6_; TMS): δ 8.49 (d, *J* = 8.5 Hz, 1H, Ar), 8.13 (s, 1H, CH), 7.99 (d, *J* = 8.0 Hz, 1H, Ar), 7.97 (d, *J* = 7.6 Hz, 1H, Ar), 7.60 (appt, *J* = 7.8 Hz, 1H, Ar), 7.40 (d, *J* = 8.5 Hz, 1H, Ar), 5.95 (s, 2H, ArCH_2_N), 5.27 (d, *J* = 4.9 Hz, 1H, OH), 4.87 (appquint, *J* = 6.3 Hz, 1H, CHOH), 1.43 (d, *J* = 6.5 Hz, 3 H, CH_3_). ^13^C NMR (101 MHz; DMSO-d_6_; TMS): δ 157.2, 153.5, 143.3, 138.7, 132.5, 130.6, 129.0, 127.9, 127.5, 122.9, 121.1, 62.1, 55.4, 24.3; HRMS (m/z): [M-H]¯ calcd. for C_14_H_13_^35^ClN_4_, 287.0705; found 287.0701.

#### 2-({4-[(benzyloxy)methyl]-1H-1,2,3-triazol-1-yl}methyl)-8-chloroquinoline (13e, C_20_H_17_ClN_4_)

Synthesized according to conditions B of the general procedure. 2-(Bromomethyl)-8-chloroquinoline (0.50 mmol, 128 mg), benzyl propargyl ether (0.50 mmol, 73 mg), sodium azide (0.50 mmol, 33 mg). After 30 min the first work-up protocol was executed and after recrystallization from EtOH-MTBE 109 mg (60%) of a pale yellow solid was obtained; mp: 65–68 °C. ^1^H NMR (400 MHz; DMSO-d_6_; TMS): δ 8.49 (d, *J* = 8.5 Hz, 1H, Ar), 8.36 (s, 1H, CH), 7.99 (d, *J* = 7.9 Hz, 1H, Ar), 7.98 (d, *J* = 7.4 Hz, 1H, Ar), 7.61 (appt, *J* = 7.8 Hz, 1H, Ar), 7.44 (d, *J* = 8.5 Hz, 1H, Ar), 7.27–7.37 (m, 5 H, Ar), 6.00 (s, 2H, ArCH_2_N), 4.63 (s, 2H, OCH_2_Ar), 4.55 (s, 2H, triazole-CH_2_OCH_2_Ar). ^13^C NMR (101 MHz; DMSO-d_6_; TMS): δ 144.7, 143.3, 138.7, 138.6, 132.5, 130.6, 129.0, 128.7, 128.1, 127.9, 127.5, 125.7, 121.1, 71.6, 63.4, 55.4; HRMS (m/z): [M-H]¯ calcd. for C_20_H_17_^35^ClN_4_, 363.1018; found 363.1021.

#### 4-{1-[(8-chloroquinolin-2-yl)methyl]-1H-1,2,3-triazol-4-yl}-N,N-dimethylaniline (13 f, C_20_H_18_ClN_5_)

Synthesized according to conditions B of the general procedure. 2-(Bromomethyl)-8-chloroquinoline (0.50 mmol, 128 mg), 4-ethynyl-*N*,*N*-dimethylaniline (0.50 mmol, 73 mg), sodium azide (0.50 mmol, 33 mg). After 40 min the first work-up protocol was executed and after recrystallization from EtOH-MTBE 95 mg (52%) of a beige solid was obtained; mp: 127–128 °C; ^1^H NMR (400 MHz; DMSO-d_6_; TMS): δ 8.55 (s, 1H, CH), 8.50 (d, *J* = 8.5 Hz, 1H, Ar), 8.00 (d, *J* = 8.0, 1H, Ar), 7.99 (d, *J* = 7.2 Hz, 1H, Ar), 7.68 (d, *J* = 8.6 Hz, 2H, Ar), 7.61 (appt, *J* = 7.8 Hz, 1H, Ar), 7.46 (d, *J* = 8.5 Hz, 1H, Ar), 6.79 (d, *J* = 8.7 Hz, 2H, Ar), 5.99 (s, 2H, ArCH_2_N), 2.93 (s, 6 H, N(CH_3_)_2_). ^13^C NMR (101 MHz; DMSO-d_6_; TMS): δ 157.1, 150.5, 147.8, 143.3, 138.8, 132.5, 130.6, 129.0, 127.9, 127.6, 126.6, 121.1, 119.1, 112.8, 55.6; HRMS (m/z): [M-H]¯ calcd. for C_20_H_18_^35^ClN_5_, 362.1178; found, 362.1174.

#### 2-[(4-phenyl-1H-1,2,3-triazol-1-yl)methyl]quinazolin-4(3 H)-one (14a, C_17_H_13_N_5_O)

Synthesized according to conditions B of the general procedure. 2-Bromomethyl-4-quinazolone (0.50 mmol, 119 mg), phenylacetylene (0.55 mol. 65 mg), sodium azide (0.50 mmol, 33 mg), t-BuOH (0.2 cm^3^, 10% v/v). After 5 h the second work-up protocol was executed. 108 mg (73%) of a white solid was obtained; mp: 283 °C; ^1^H NMR (400 MHz; DMSO-d_6_; TMS): δ 12.67 (s, 1H, OCNH), 8.70 (s, 1H, CH), 8.17–8.09 (dd, *J* = 7.9 Hz, 1.0 Hz, 1H, Ar), 7.89 (appd, *J* = 7.3 Hz, 2H, Ar), 7.82–7.74 (appt, 1H, Ar), 7.55 (m, 2H, Ar), 7.47 (t, *J* = 7.6 Hz, 2H, Ar), 7.35 (appt, *J* = 7.4 Hz, 1H, Ar), 5.67 (s, 2H, ArCH_2_N); ^13^C NMR (101 MHz; DMSO-d_6_; TMS): δ 161.9, 151.5, 148.6, 146.8, 135.0, 131.1, 129.4, 128.4, 127.7, 127.4, 126.3, 125.7, 123.4, 121.8, 51.9; HRMS (m/z): [M-H]¯ calcd. for C_17_H_13_N_5_O, 302.1047; found, 302.1042.

#### 2-[(4-butyl-1H-1,2,3-triazol-1-yl)methyl]quinazolin-4(3H)-one (14b, C_15_H_17_N_5_O)

Synthesized according to conditions B of the general procedure. 2-Bromomethyl-4-quinazolone (0.50 mmol, 119 mg), 1-hexyne (0.55 mmol, 45 mg), sodium azide (0.50 mmol, 33 mg), t-BuOH (0.2 cm^3^, 10% v/v); After 1.5 h the second work-up protocol was executed. 103 mg (73%) of a beige solid was obtained; mp: 262 °C; ^1^H NMR (400 MHz; DMSO-d_6_; TMS): δ 12.58 (s, 1H, OCNH), 8.12 (dd, *J* = 8.3 Hz, 1.5 Hz, 1H, Ar), 7.97 (s, 1H, CH), 7.82–7.76 (m, 1H, Ar), 7.55–7.50 (m, 2H, Ar), 5.53 (s, 2H, ArCH_2_N), 2.65 (t, *J* = 7.5 Hz, 2H, CH_2_CH_2_CH_2_CH_3_), 1.60 (tt, *J* = 7.5 Hz, 2H, CH_2_CH_2_CH_2_CH_3_), 1.35 (tq, *J* = 7.3 Hz, 2H, CH_2_CH_2_CH_2_CH_3_), 0.91 (t, *J* = 7.4 Hz, 3 H, CH_2_CH_2_CH_2_CH_3_); ^13^C NMR (101 MHz; DMSO-d_6_; TMS): δ 161.8, 151.7, 148.6, 147.3, 135.0, 127.6, 127.4, 126.3, 123.8, 121.7, 51.6, 31.5, 25.1, 22.1, 14.1; HRMS (m/z): [M-H]¯ calcd. for C_15_H_17_N_5_O, 282.1360; found, 282.1355.

#### 2-{[4-(hydroxymethyl)-1H-1,2,3-triazol-1-yl]methyl}quinazolin-4(3H)-one (14c, C_12_H_11_N_5_O_2_)

Synthesized according to conditions B of the general procedure. 2-Bromomethyl-4-quinazolone (0.50 mmol, 119 mg), propargyl alcohol (0.55 mmol, 31 mg), sodium azide (0.50 mmol, 33 mg), t-BuOH (0.2 cm^3^, 10% v/v); After 1 h the second work-up protocol was executed. 85 mg (70%) of an off-white solid was obtained; mp: 235 °C; ^1^H NMR (400 MHz; DMSO-d_6_; TMS): δ 12.57 (s, 1H, OCNH), 8.13 (d, *J* = 1.2 Hz, 1H, Ar), 8.10 (s, 1H, CH), 7.83–7.75 (m, 1H, Ar), 7.53 (dd, *J* = 13.8 Hz, 7.3 Hz, 2H, Ar), 5.58 (s, 2H, ArCH_2_N), 5.22 (s, 1H, OH), 4.56 (s, 2H, CH_2_OH); ^13^C NMR (101 MHz; DMSO-d_6_; TMS): δ 161.9, 151.7, 148.5, 148.5, 135.0, 127.6, 127.4, 126.3, 124.6, 121.7, 55.5, 51.7; HRMS (m/z): [M-H]¯ calcd. for C_12_H_11_N_5_O_2_, 256.0840; found, 256.0835.

#### 2-{[4-(1-hydroxyethyl)-1H-1,2,3-triazol-1-yl]methyl}quinazolin-4(3H)-one (14d, C_13_H_13_N_5_O_2_)

Synthesized according to conditions B of the general procedure. 2-Bromomethyl-4-quinazolone (0.50 mmol, 119 mg), but-3-yn-2-ol (0.55 mmol, 39 mg), sodium azide (0.50 mmol, 33 mg), t-BuOH (0.2 cm^3^, 10% v/v). After 30 min the second work-up protocol was executed. 97 mg (72%) of a beige solid was obtained; mp: 244 °C; ^1^H NMR (400 MHz; DMSO-d_6_; TMS): δ 12.63 (s, 1H, OCNH), 8.12 (d, *J* = 7.7 Hz, 1H, Ar), 8.05 (s, 1H, CH), 7.80 (t, *J* = 7.4 Hz, 1H, Ar), 7.54 (dd, *J* = 15.8 Hz, 7.9 Hz, 2H, Ar), 5.55 s, (2H, ArCH_2_N), 5.29 (d, *J* = 4.8 Hz, 1H, OH), 4.94–4.78 (m, 1H, CHOH), 1.43 (d, *J* = 6.5 Hz, 3 H, CH_3_). ^13^C NMR (101 MHz; DMSO-d_6_; TMS): δ 161.9, 153.1, 151.7, 148.6, 135.0, 127.6, 127.4, 126.3, 123.2, 121.7, 62.1, 51.8, 24.2; HRMS (m/z): [M-H]¯ calcd. for C_13_H_13_N_5_O_2_, 270.0996; found, 270.0992.

#### 2-({4-[(benzyloxy)methyl]-1H-1,2,3-triazol-1-yl}methyl)quinazolin-4(3H)-one (14e, C_19_H_17_N_5_O_2_)

Synthesized according to conditions B of the general procedure. 2-Bromomethyl-4-quinazolone (0.50 mmol, 119 mg), benzyl propargyl ether (0.55 mmol, 80 mg), sodium azide (0.50 mmol, 33 mg), t-BuOH (0.2 cm^3^, 10% v/v). After 15 min, the second work-up protocol was executed. 164 mg (95%) of a white solid was obtained; mp: 226 °C; ^1^H NMR (400 MHz; DMSO-d_6_; TMS): δ 12.64 (s, 1H, OCNH), 8.27 (s, 1H, CH), 8.12 (dd, *J* = 8.2 Hz, 1.3 Hz, 1H, Ar), 7.81–7.74 (m, 1H, Ar), 7.52 (appt, *J* = 7.1 Hz, 2H, Ar), 7.38–7.33 (m, 4 H, Ar), 7.30 (td, *J* = 8.7 Hz, 4.1 Hz, 1H, Ar), 5.61 (s, 2H, ArCH_2_N), 4.63 (s, 2H, ArCH_2_O), 4.56 (s, 2H, OCH_2_Ar); ^13^C NMR (101 MHz; DMSO-d_6_; TMS): δ 161.9, 151.7, 148.5, 144.4, 138.6, 135.0, 128.7, 128.1, 128.0, 127.5, 127.4, 126.3, 126.0, 121.8, 71.7, 63.3, 51.7; HRMS (m/z): [M-H]^−^ calcd. for C_19_H_17_N_5_O_2_, 346.1309; found, 346.1303.

#### 2-({4-[4-(dimethylamino)phenyl]-1H-1,2,3-triazol-1-yl}methyl)quinazolin-4(3H)-one (14 f, C_19_H_18_N_6_O)

Synthesized according to conditions B of the general procedure. 2-Bromomethyl-4-quinazolone (0.50 mmol, 119 mg), 4-ethynyl-*N*,*N*-dimethylaniline (0.55 mmol, 79 mg), sodium azide (0.50 mmol, 33 mg), t-BuOH (0.2 cm^3^, 10% v/v). After 30 min the second work-up protocol was executed. 152 mg (88%) of a light brown solid was obtained; mp: 276 °C; ^1^H NMR (400 MHz; DMSO-d_6_; TMS): δ 12.65 (s, 1H, OCNH), 8.47 (s, 1H, CH), 8.13 (d, *J* = 7.8 Hz, 1H, Ar), 7.78 (t, *J* = 7 .6 Hz, 1H, Ar), 7.69 (d, *J* = 8.7 Hz, 2H, Ar), 7.54 (dd, *J* = 17.8 Hz, 7.9 Hz, 2H, Ar), 6.79 (d, *J* = 8.7 Hz, 2H, Ar), 5.61 (s, 2H, ArCH_2_N), 2.94 (s, 6 H, N(CH_3_)_2_). ^13^C NMR (101 MHz; DMSO-d_6_; TMS): δ 161.9, 151.6, 150.5, 148.6, 147.4, 135.0, 127.6, 127.4, 126.6, 126.3, 121.8, 121.4, 119.0, 112.8, 51.8; HRMS (m/z): [M-H]¯ calcd. for C_19_H_18_N_6_O, 345.1469; found, 345.1463.

### Organic azides precursors

#### 2-(bromomethyl)-quinoline (12)

The compound was synthesized following the reported protocol^42^. A white solid yield: 42%; ^1^H NMR (400 MHz; DMSO-d_6_; TMS): δ 8.16 (d, *J* = 8.4 Hz, 1H, ArH), 8.07 (d, *J* = 8.4 Hz, 1H, ArH), 7.80 (dd, *J* = 8.0 Hz, 1.2Hz, 1H, ArH), 7.74–7.70 (m, 1H, ArH), 7.57–7.52 (m, 2H, ArH), 4.71 (s, 2H, BrCH_2_);

#### 2-(bromomethyl)-8-chloroquinoline (13)

8-Chloro-2-methylquinoline (1.78 g, 10 mmol) and NBS (1.78 g, 10 mmol) was loaded into a three-neck 250 cm^3^ flask. The neck was equipped with a reflux condenser, a thermometer and a septum stopper. Ethyl acetate (100 cm^3^) and water (50 cm^3^) were introduced and the reaction vessel was flushed with nitrogen and finally the condenser was attached to a nitrogen-filled balloon. A magnetic stirrer was engaged and the flask was immersed into an oil bath. At the point at which the temperature of the reaction mixture exceeded 70 °C, 1 cm^3^ of an AIBN hydrochloride (20 mg, 0.12 mmol) aqueous solution was introduced dropwise over two-hour period through the septum. After 4 h, the organic phase was separated and washed with water (2 × 100 cm^3^). Silica-gel (40 g) was loaded into the solution and the solvent was removed under reduced pressure. The material was packed into a chromatographic column and eluted with hexanes-CH_2_Cl_2_ (3:1 v/v). The product was collected as a solution in a second fraction. Ethyl acetate was removed under reduced pressure to yield 768 mg (30%) as white crystals, mp: 129 °C (lit.^43^ 127 °C); ^1^H NMR (400 MHz; DMSO-d_6_; TMS): δ 8.51 (d, *J* = 8.5 Hz, 1H, ArH-3), 8.00 (dd, *J* = 7.7 Hz, 1.4 Hz, 1H, ArH-7), 7.98 (dd, *J* = 6.9 Hz, 1.3 Hz, 1H, ArH-5), 7.80 (d, *J* = 8.5 Hz, 1H, ArH-4), 7.62 (dd, *J* = 8.1 Hz, 7.6 Hz, 1H, ArH-6), 4.91 (s, 2H, ArCH_2_Br).

#### 2-(bromomethyl)-3H-quinazolin-4-one (14)

The compound was prepared following the reported protocol^44^. M.p.: 229–231 °C; ^1^H NMR (400 MHz; DMSO-d_6_; TMS): δ 12.57 (s, 1H), 8.13 (d, *J* = 7.9 Hz, 1H), 7.84 (t, *J* = 7.6 Hz, 1H), 7.68 (d, *J* = 8.1 Hz, 1H), 7.55 (t, *J* = 7.5 Hz, 1H), 4.41 (s, 2H).

#### 2-(iodomethyl)-3H-quinazolin-4-one (15, C_9_H_7_IN_2_O)

A suspension of 2-methyl-4(3H)-quinazolinone (1.6 g, 10 mmol) and iodine (2.54 g, 10 mmol) in 50 cm^3^ of MeOH was refluxed in a balloon assembly for 16 h). The precipitate was filtered and rinsed on a funnel with a portion of MeOH in order to obtain the product – 1.74 g (61%) as a white fluffy solid that was used without further purification, mp: 219–221 °C (decomp.);^1^H NMR (500 MHz; DMSO-d_6_; TMS): δ 12.44 (s, 1H, NH), 8.10 (ddd, *J* = 7.9 Hz, 1.6 Hz, 0.5 Hz, 1H, ArH-5), 7.81 (ddd, *J* = 8.2 Hz, 7.1 Hz, 1.6 Hz, 1H, ArH-7), 7.63 (ddd, *J* = 8.2 Hz, 1.1 Hz, 0.6 Hz, 1H, ArH-8), 7.52 (ddd, *J* = 8.0 Hz, 7.1 Hz, 1.1 Hz, 1H, ArH-6), 4.25 (s, 2H, ArCH_2_I); ^13^C NMR (101 MHz; DMSO-d_6_; TMS): δ 162.0, 155.3, 149.0, 135.1, 127.43, 127.36, 126.3, 121.3, 55.4; HRMS (m/z): [M + H]^+^ calcd. for C_9_H_7_IN_2_O, 286.9676; found, 286.9676.

### Data availability

The data generated or analysed during this study are included in this published article or in its Supplementary Information files.

## Electronic supplementary material

Supplementary Information
