# Supplementary material for: Electrolytic copper as cheap and effective catalyst for one-pot triazole synthesis
Source: Sci Rep. 2018 Mar 14;8:4496. doi: 10.1038/s41598-018-22703-0 (PMC5852211; doi:10.1038/s41598-018-22703-0)

Electronic Supplementary Information

Electrolytic copper as cheap and effective catalyst for one-pot triazole synthesis

Jacek Mularski [1] ● Barbara Czaplińska[1] ● Wioleta Cieślik[1] ● Jakub Bebłot [1] ● Piotr Bartczak[1]
Rafał Sitko[1] ● Jarosław Polański [1] ● Robert Musioł[1]

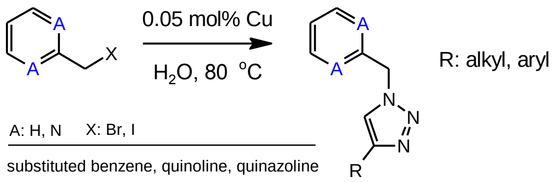

✉ Robert Musioł
robert.musiol@us.edu.pl

1 Institute of Chemistry, University of Silesia, Katowice, Poland

1-benzyl-4-phenyl-1H-1,2,3-triazole (**1a**)

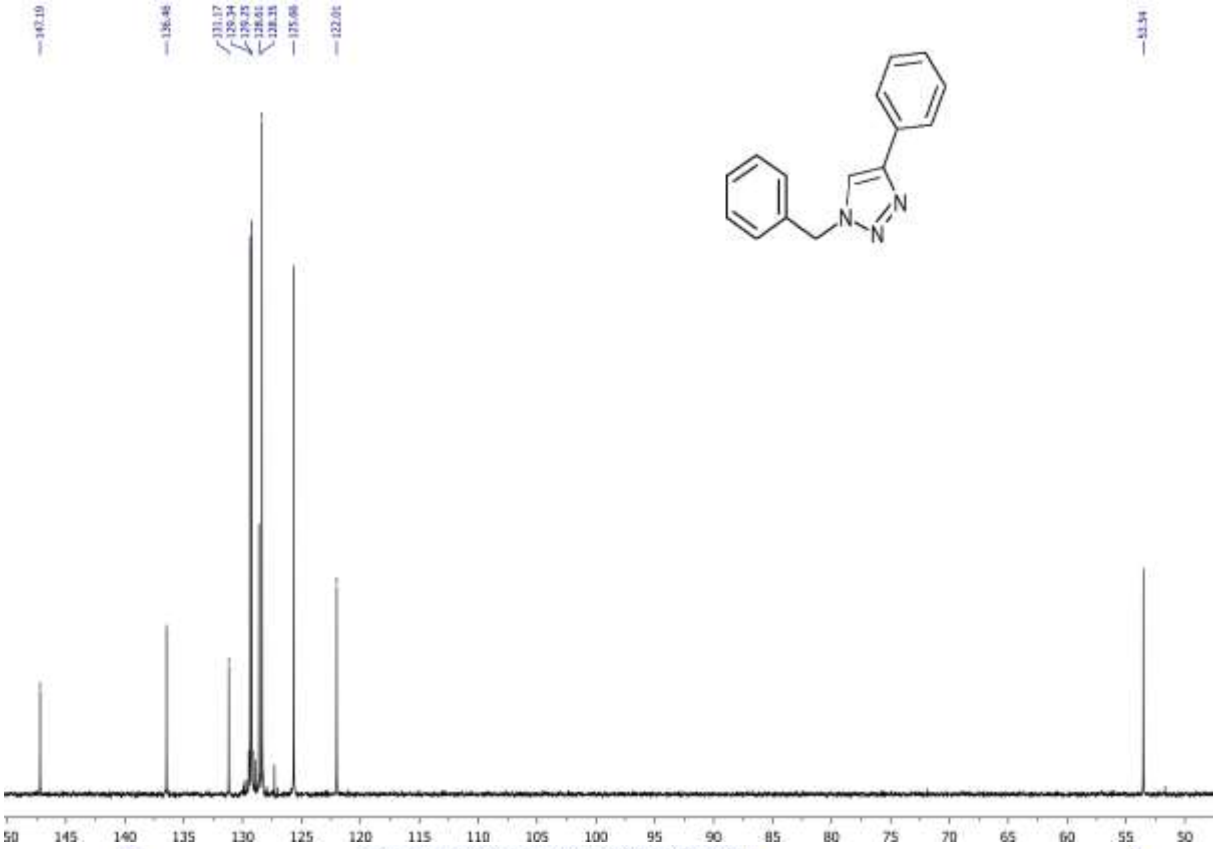

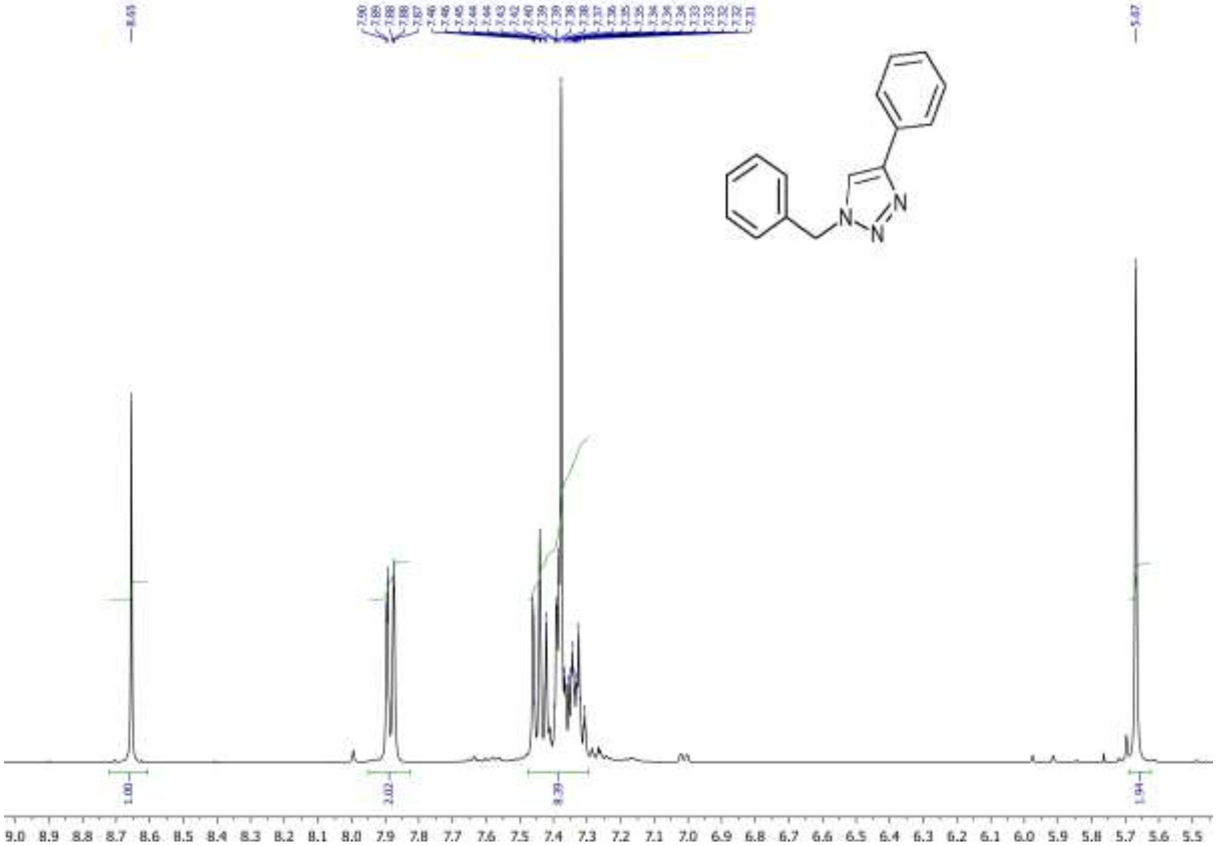

1-[(4-bromophenyl)methyl]-4-phenyl-1H-1,2,3-triazole (**2a**)

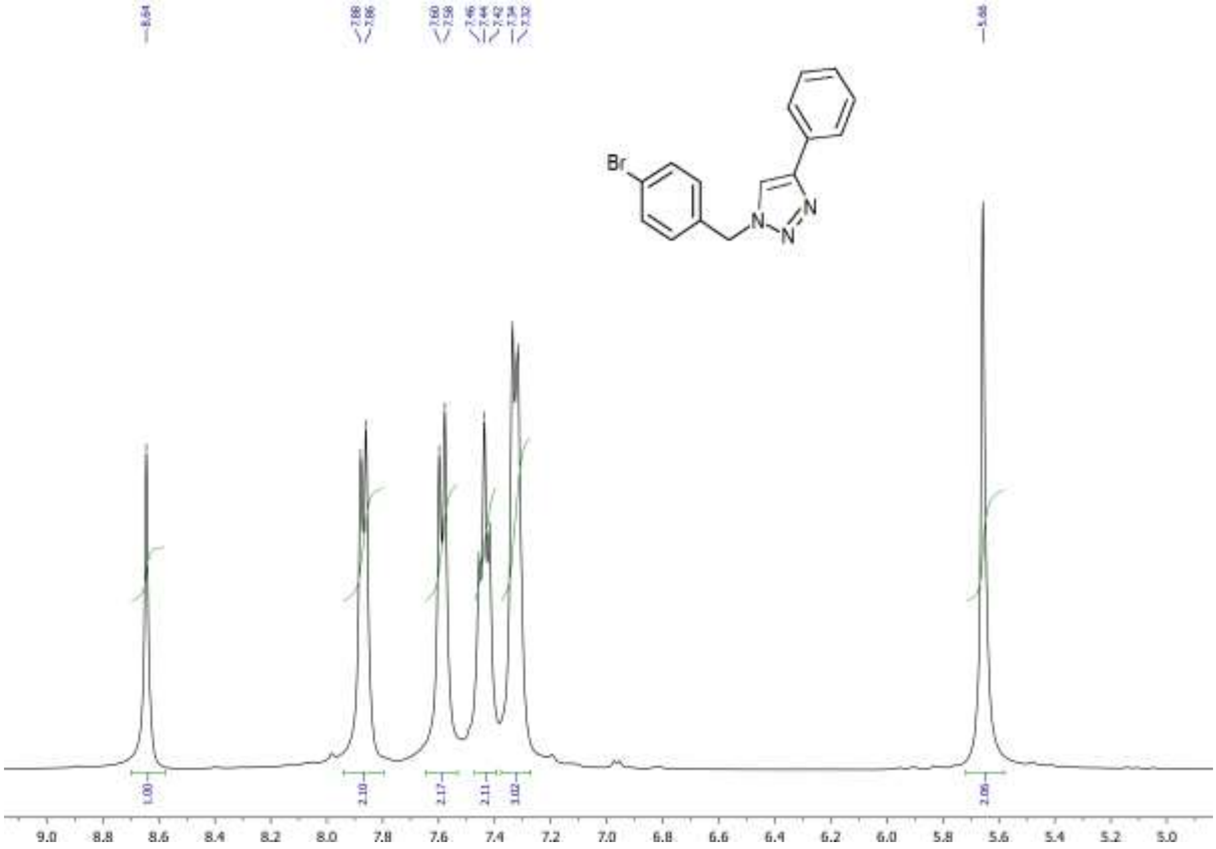

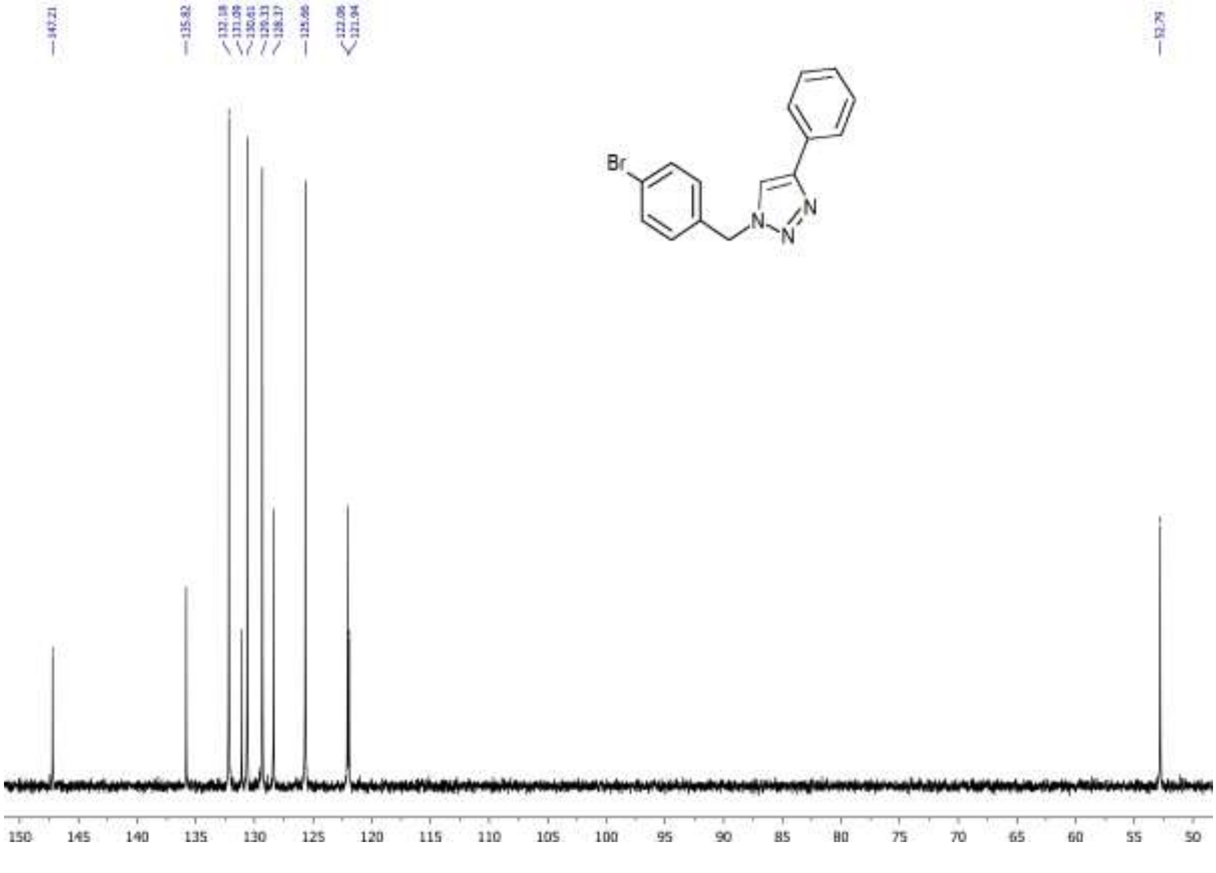

1-[(4-chlorophenyl)methyl]-4-phenyl-1H-1,2,3-triazole (**3a**)

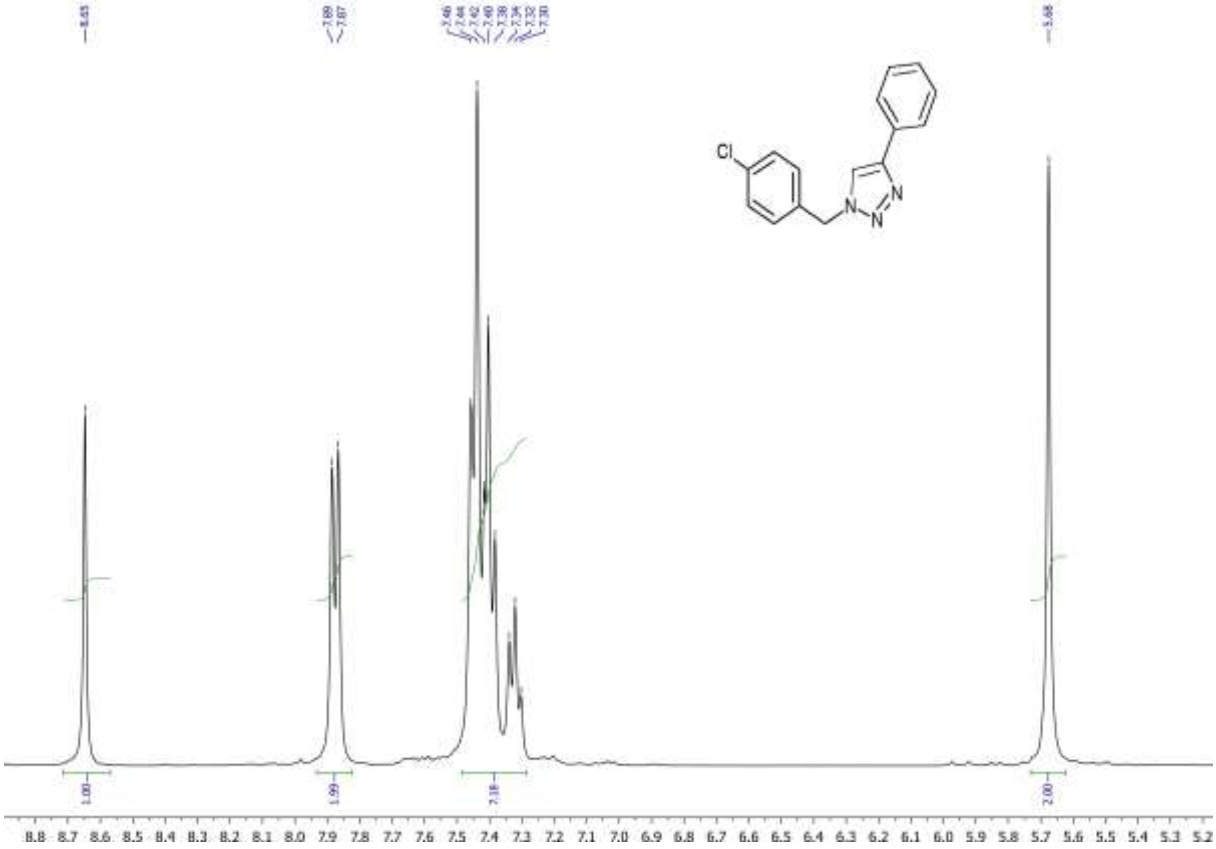

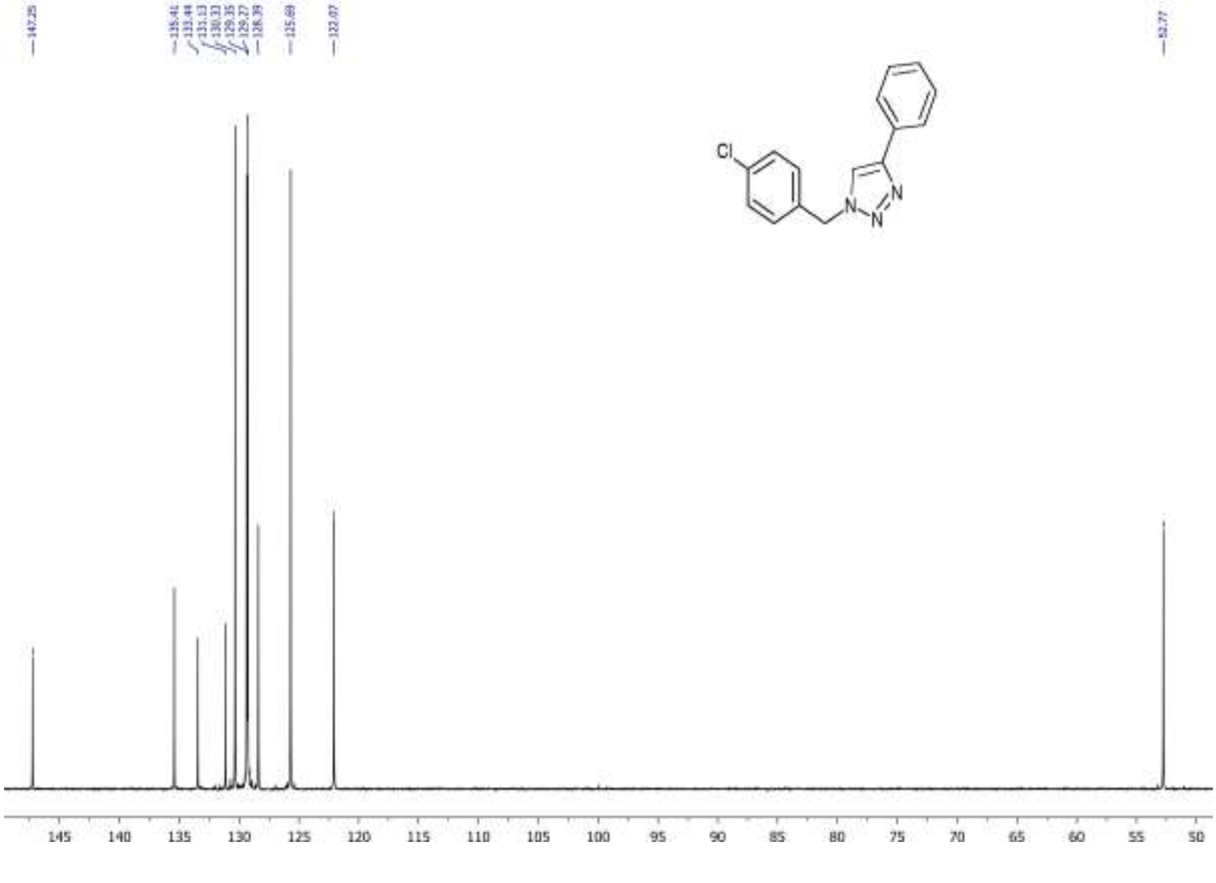

1-[(4-iodophenyl)methyl]-4-phenyl-1H-1,2,3-triazole (**4a**)

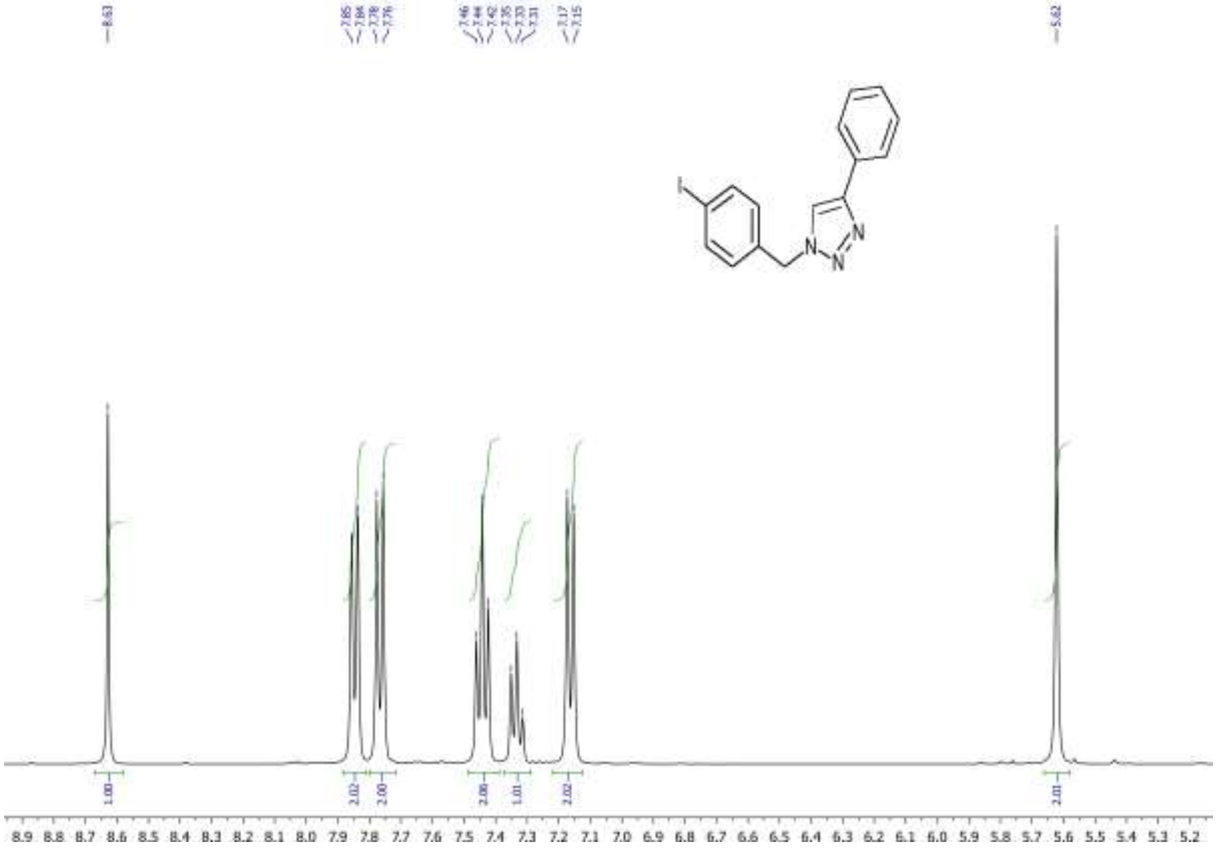

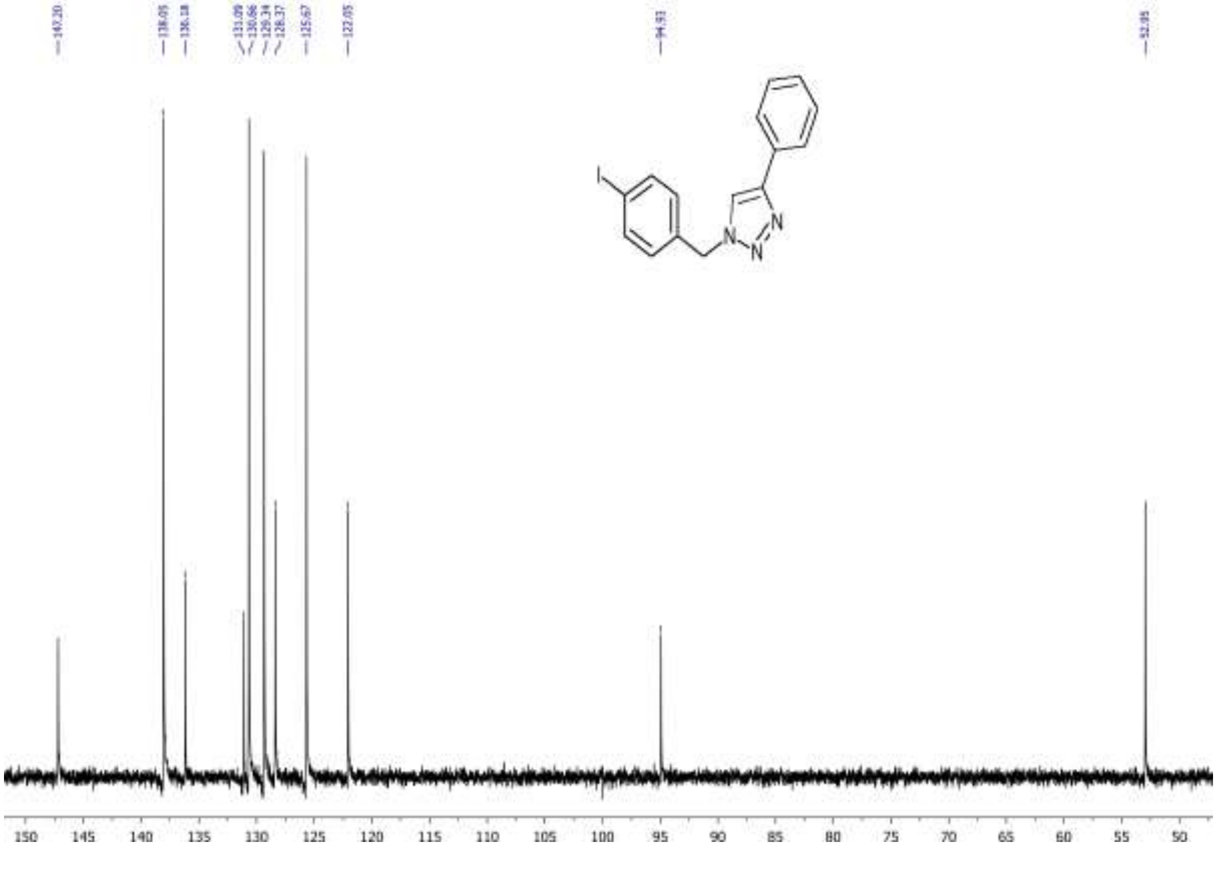

1-[(2,3-dichlorophenyl)methyl]-4-phenyl-1H-1,2,3-triazole (**5a**)

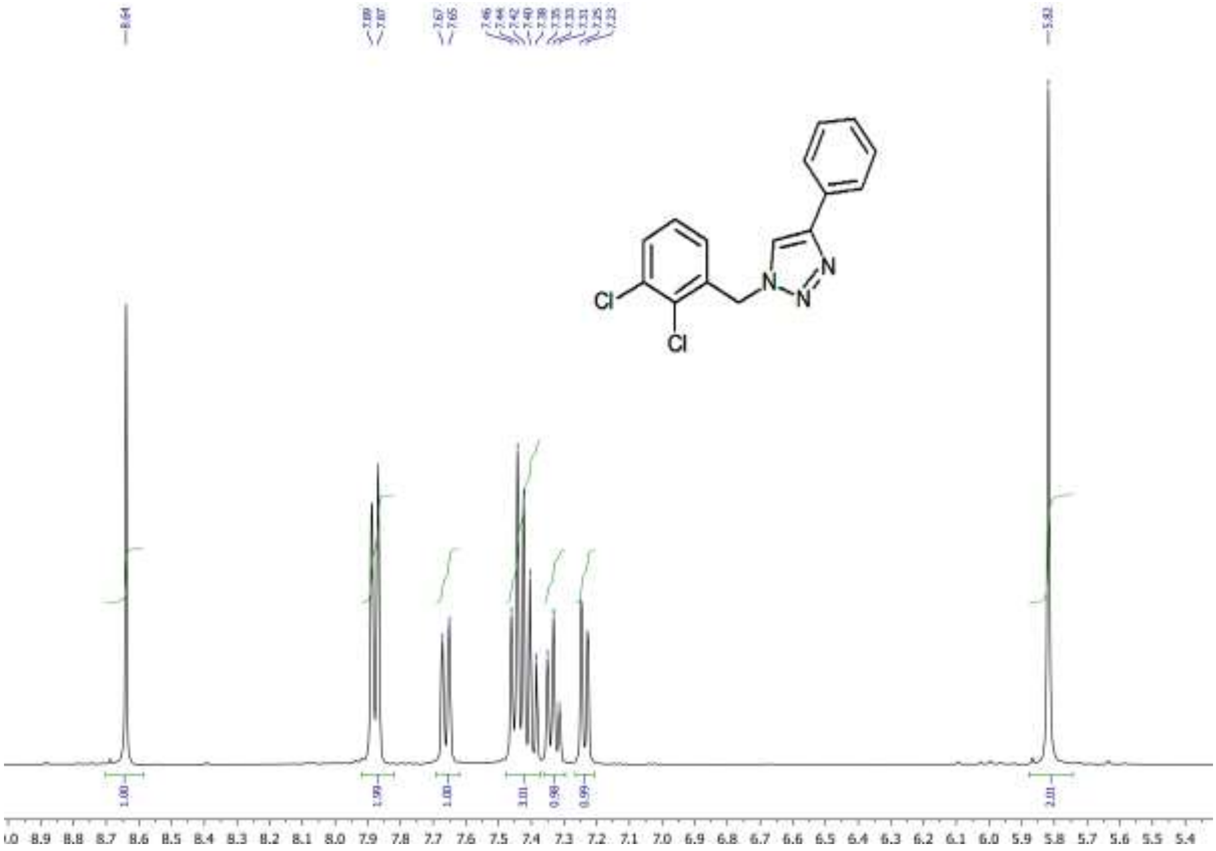

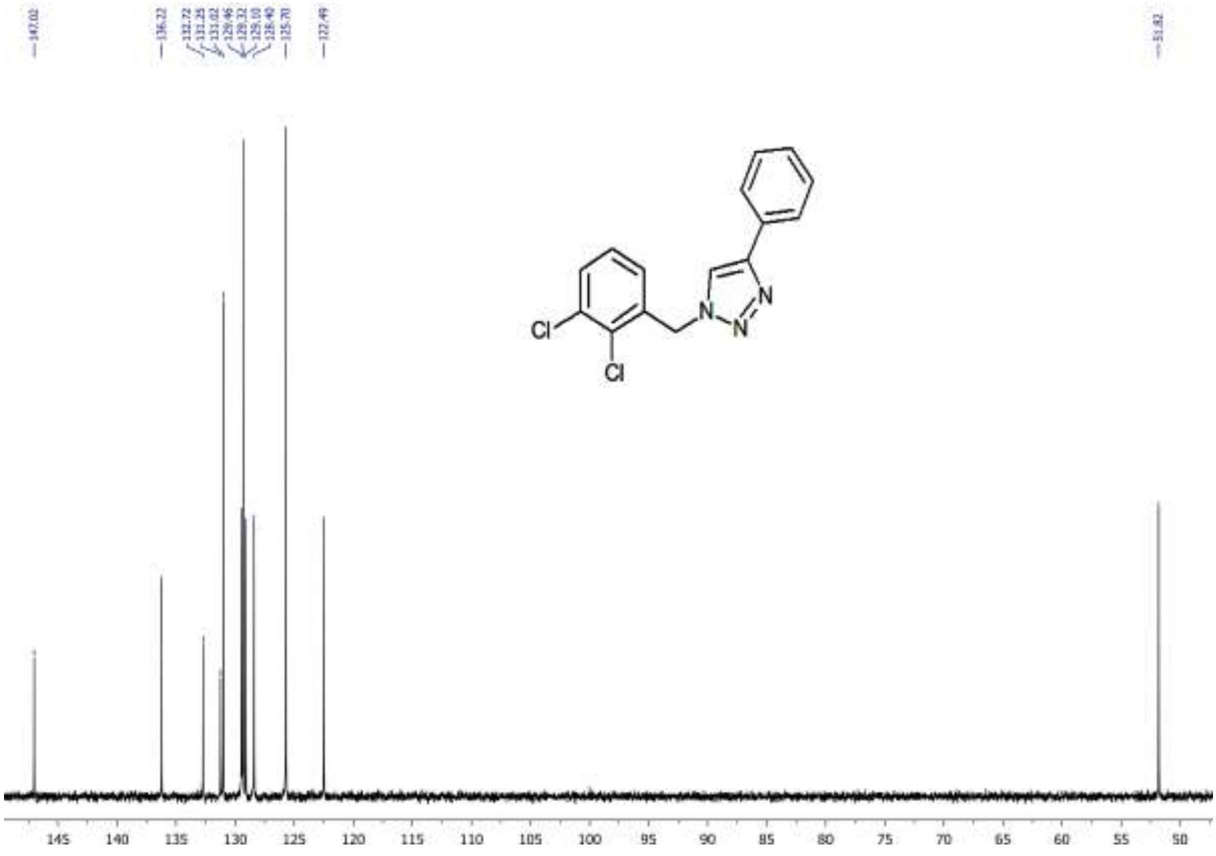

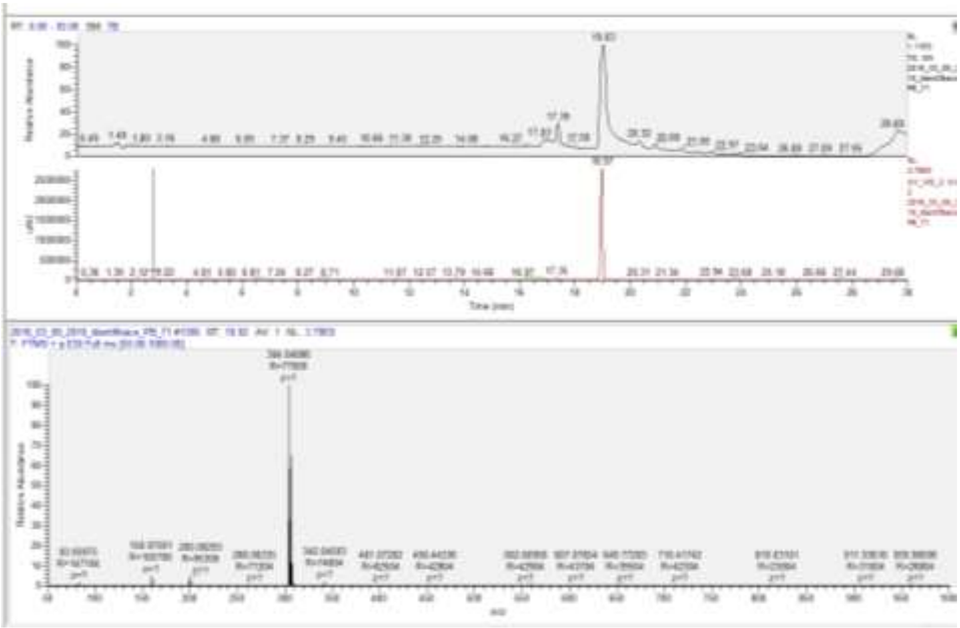

1-benzyl-4-butyl-1H-1,2,3-triazole (**1b**)

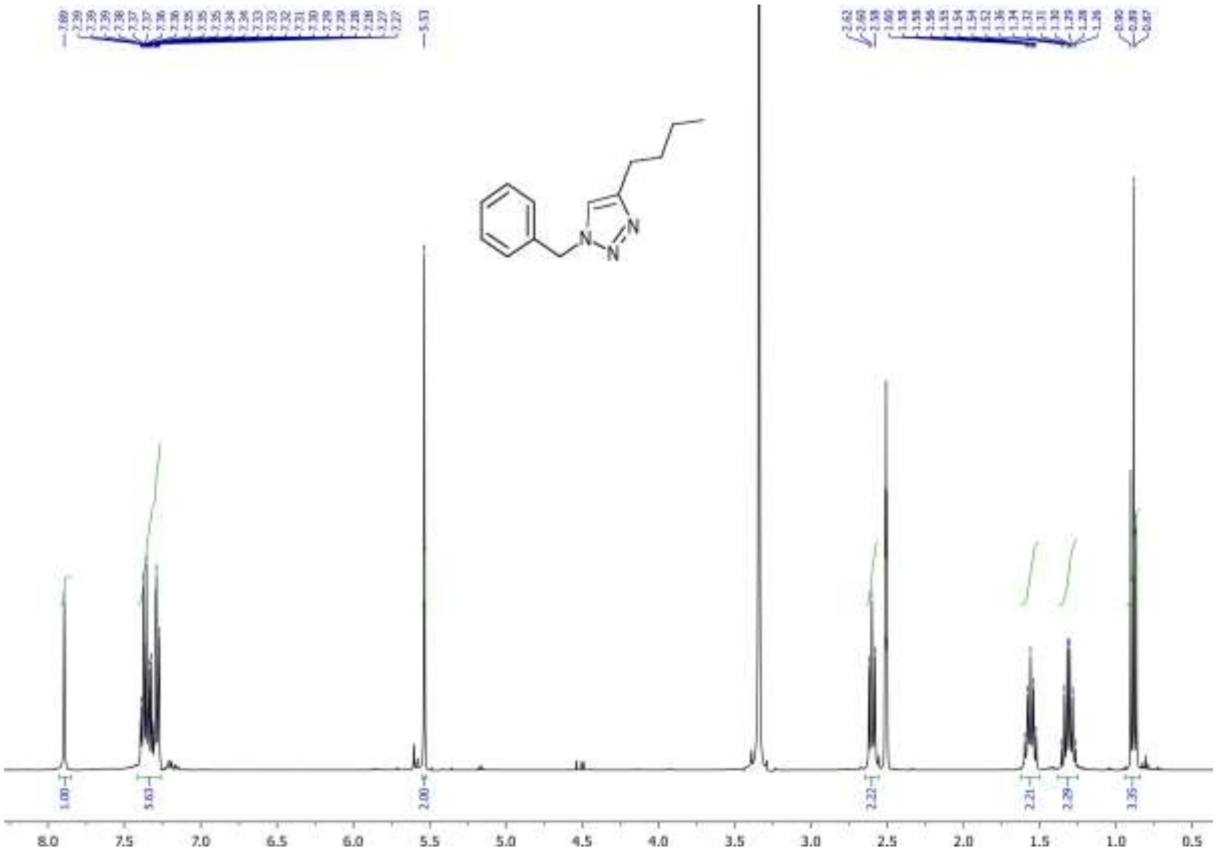

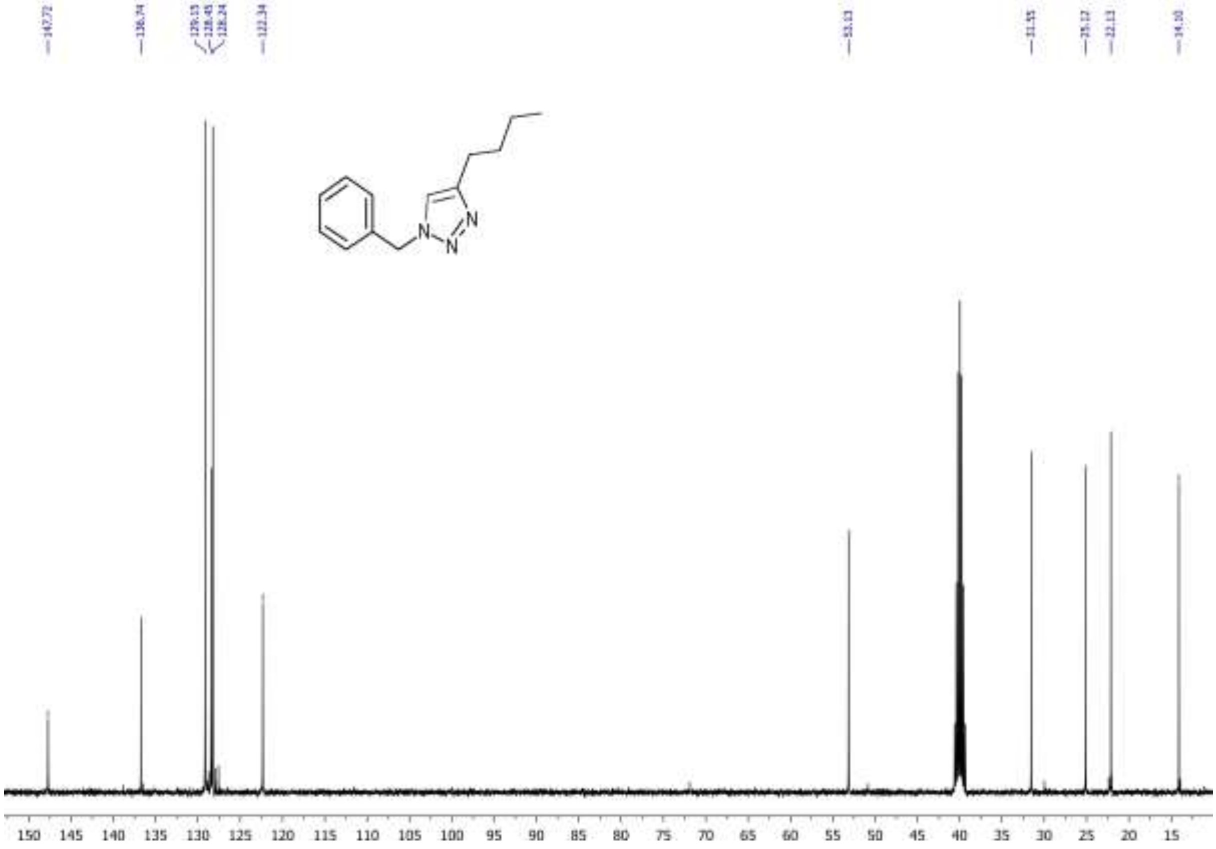

1-[(4-bromophenyl)methyl]-4-butyl-1H-1,2,3-triazole (**2b**)

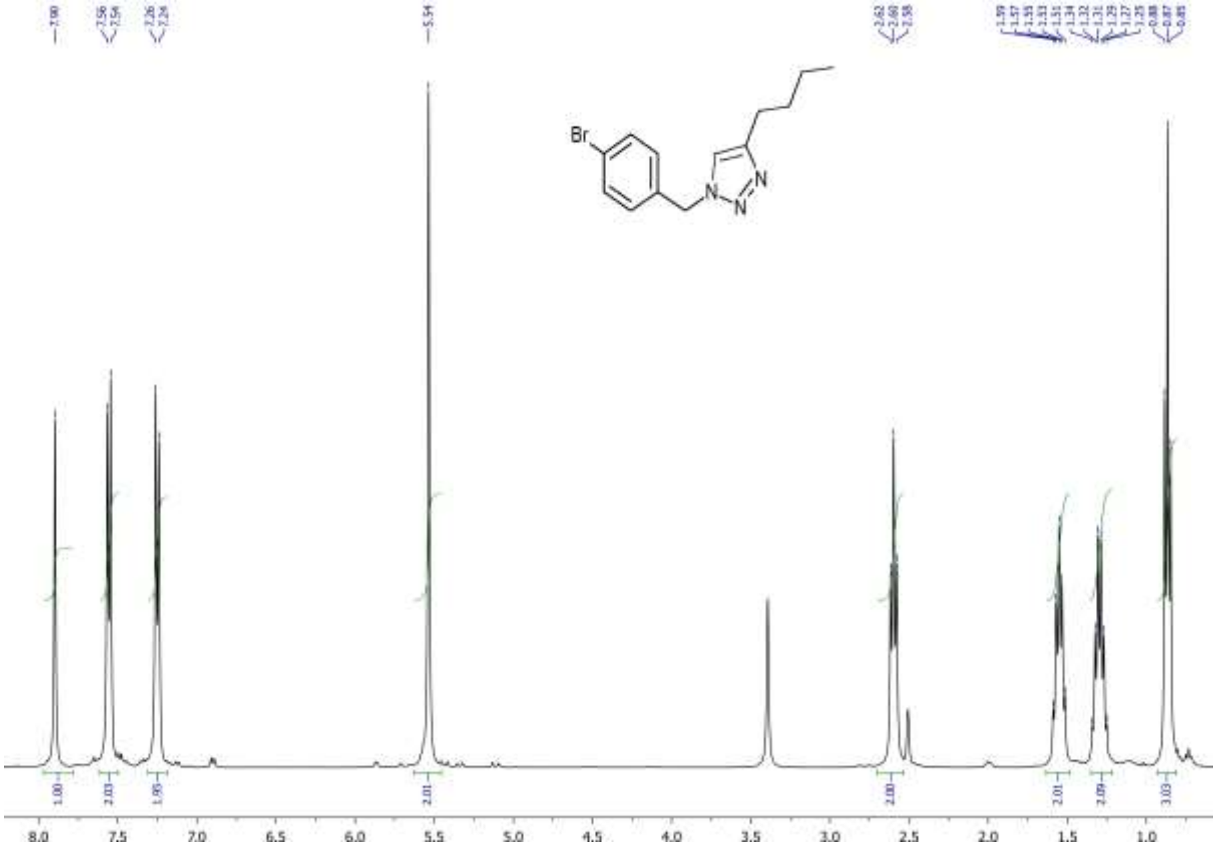

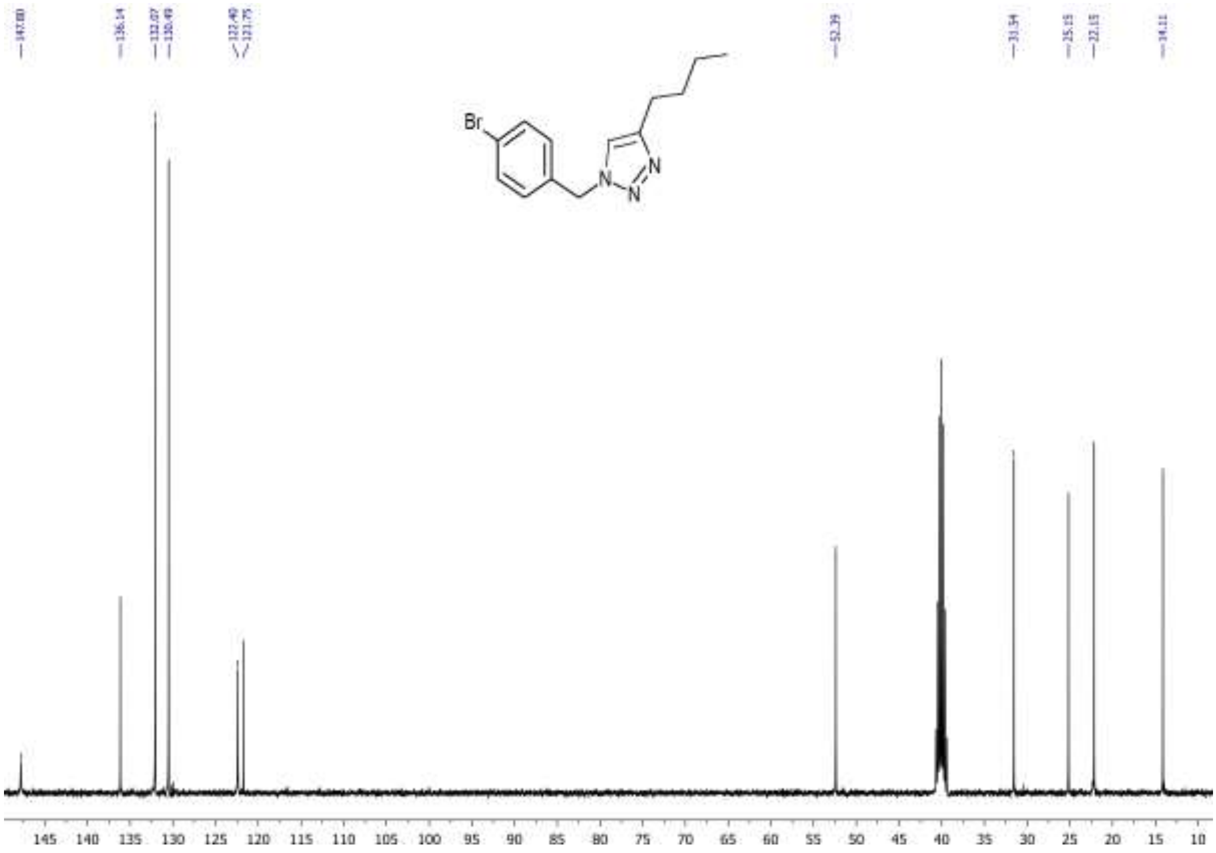

4-butyl-1-[(4-chlorophenyl)methyl]-1H-1,2,3-triazole (**3b**)

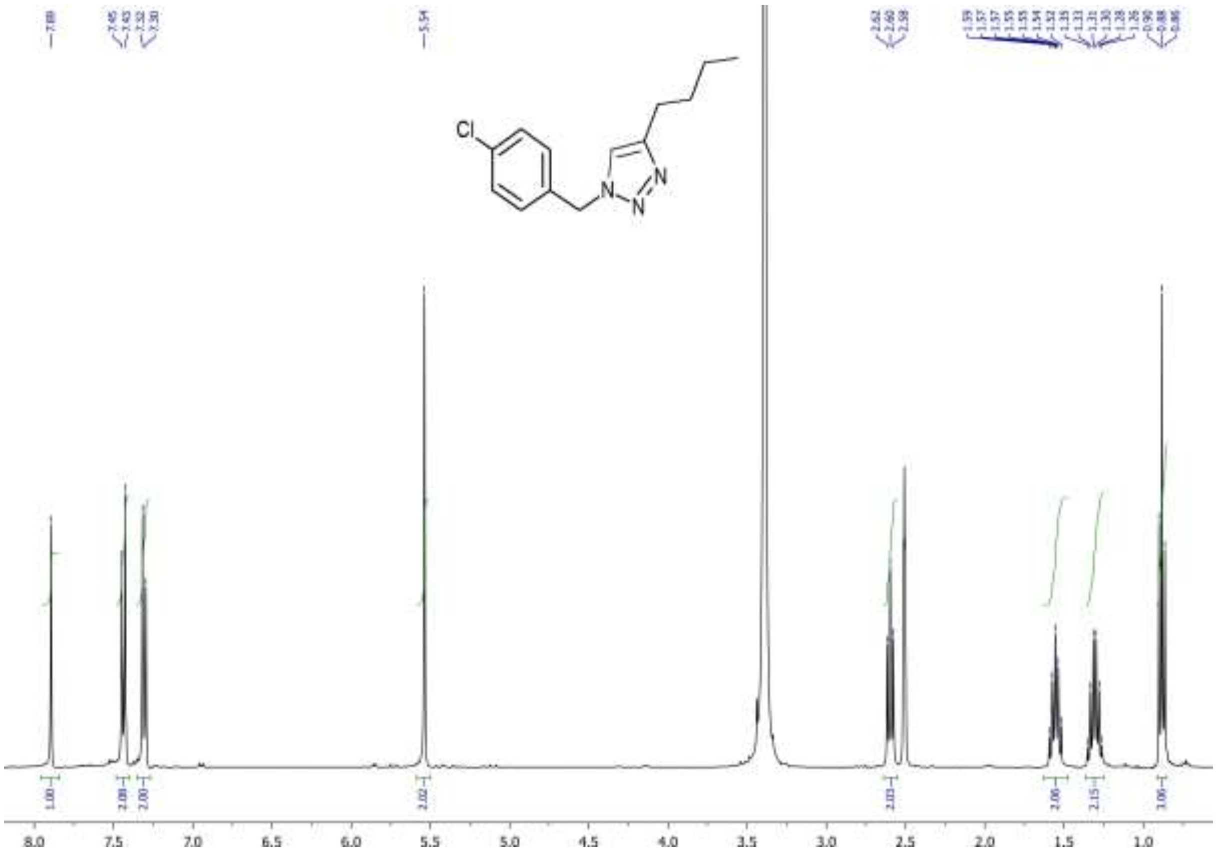

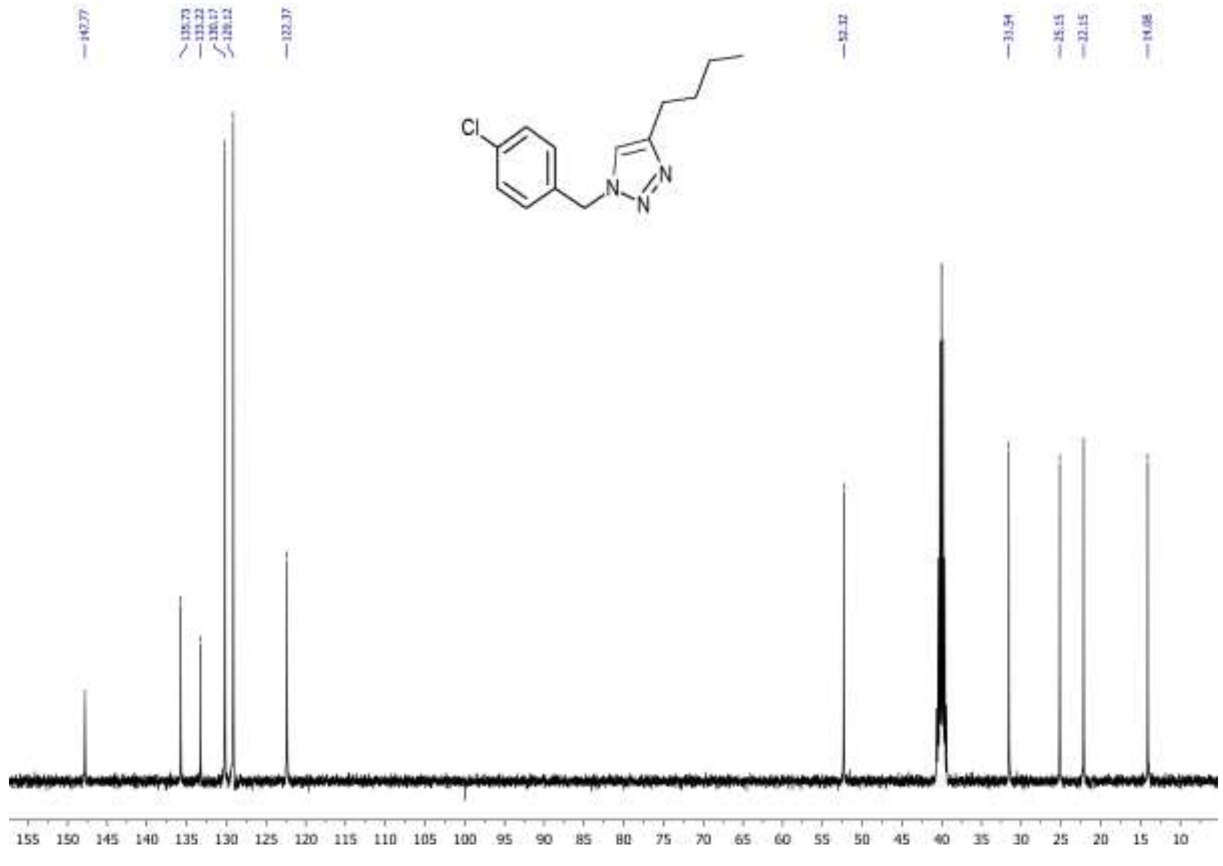

4-butyl-1-[(4-iodophenyl)methyl]-1H-1,2,3-triazole (**4b**)

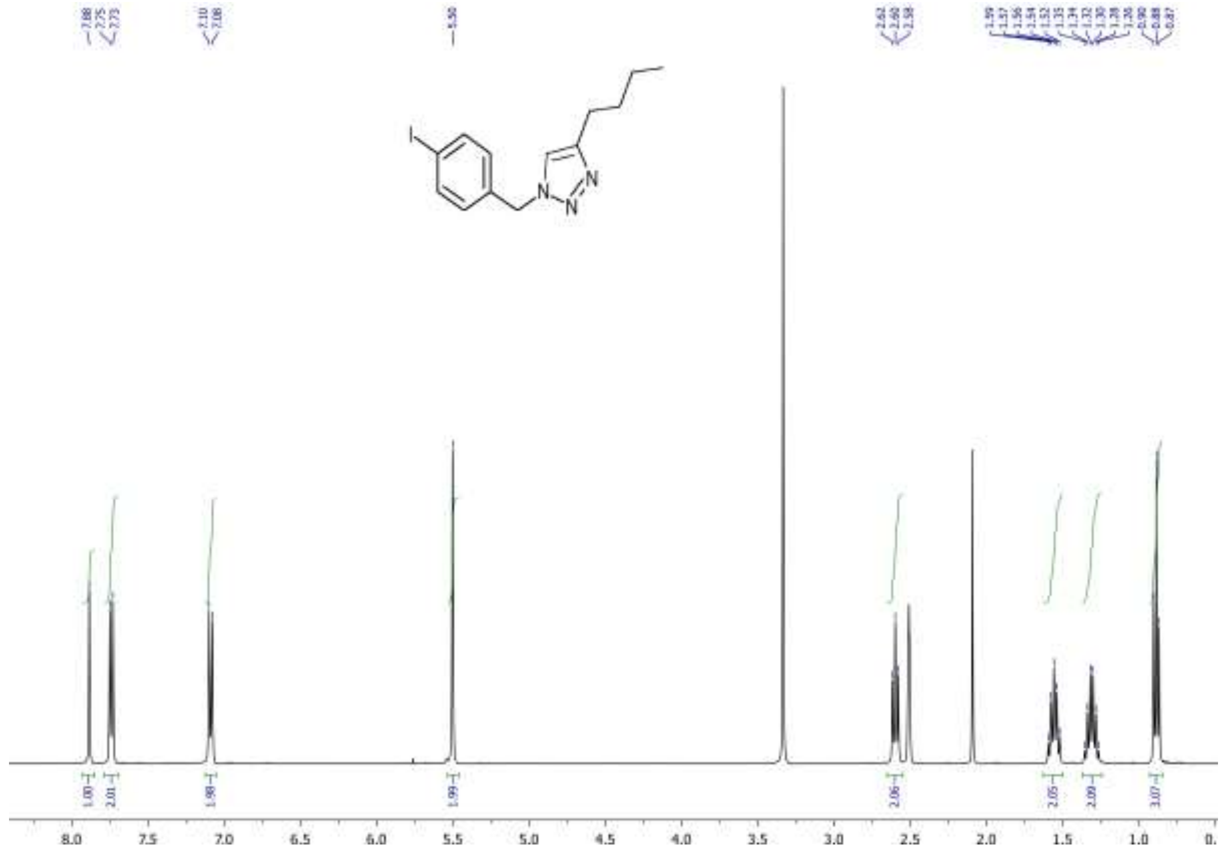

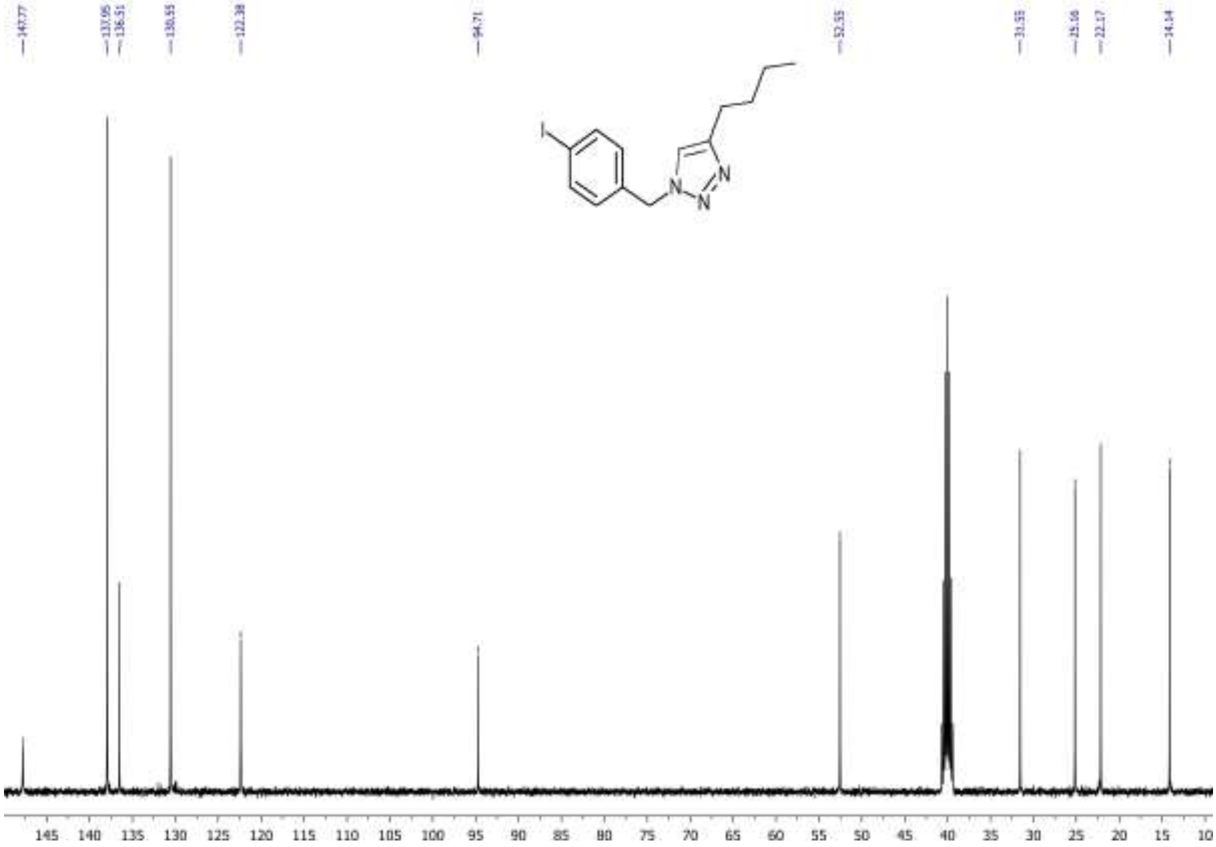
147.77
137.95
136.51
130.55
122.38
94.71
52.55
31.55
25.16
22.17
14.14
145 140 135 130 125 120 115 110 105 100 95 90 85 80 75 70 65 60 55 50 45 40 35 30 25 20 15 10

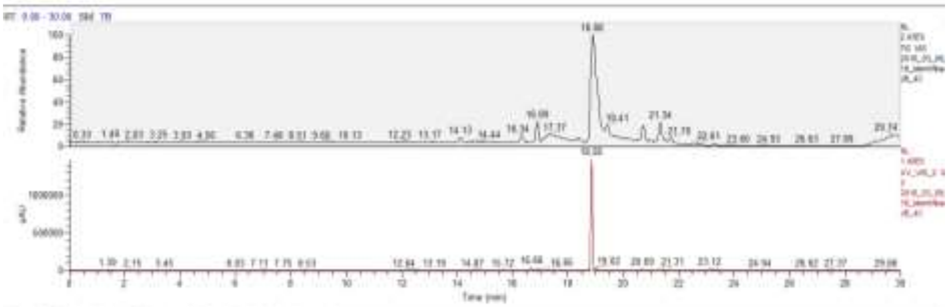

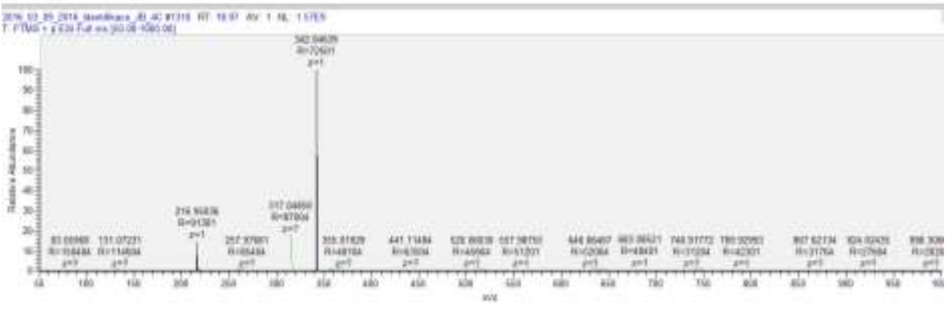

4-butyl-1-[(2,3-dichlorophenyl)methyl]-1H-1,2,3-triazole (**5b**)

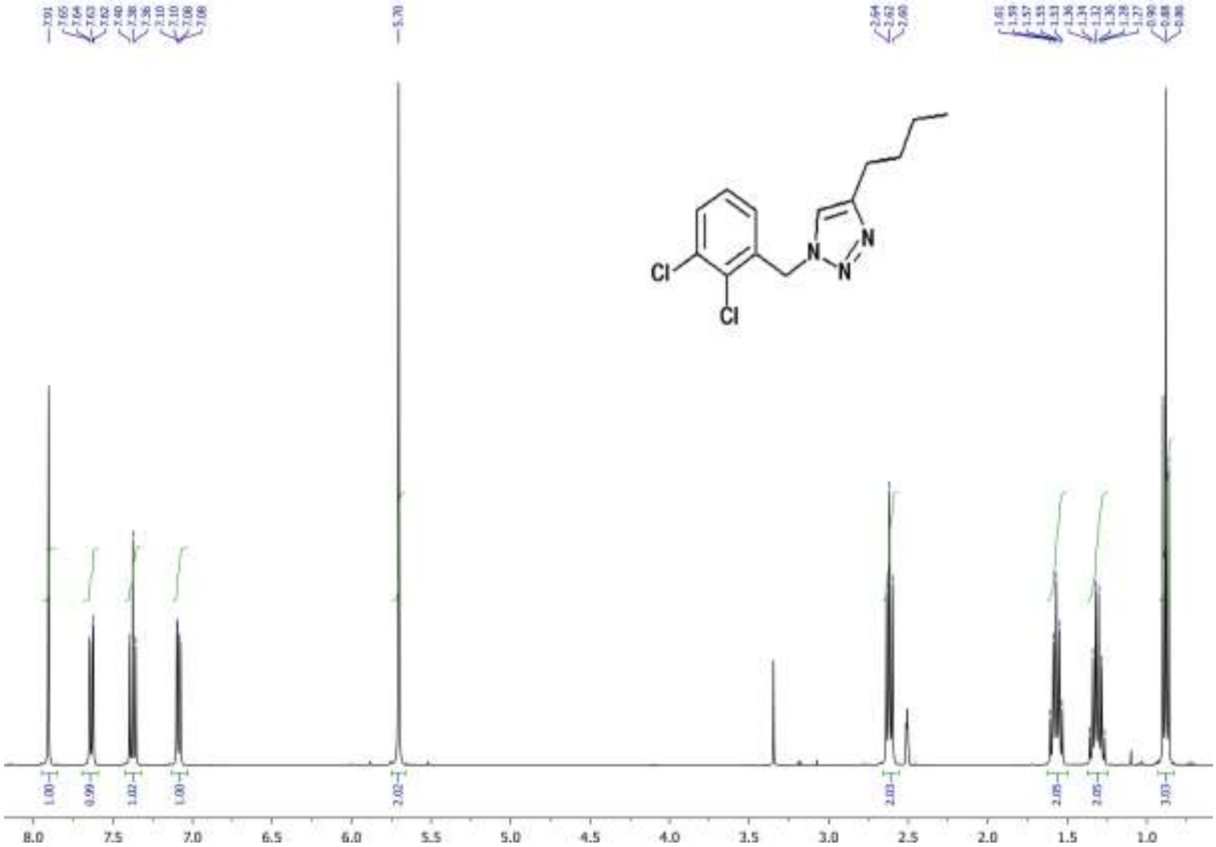

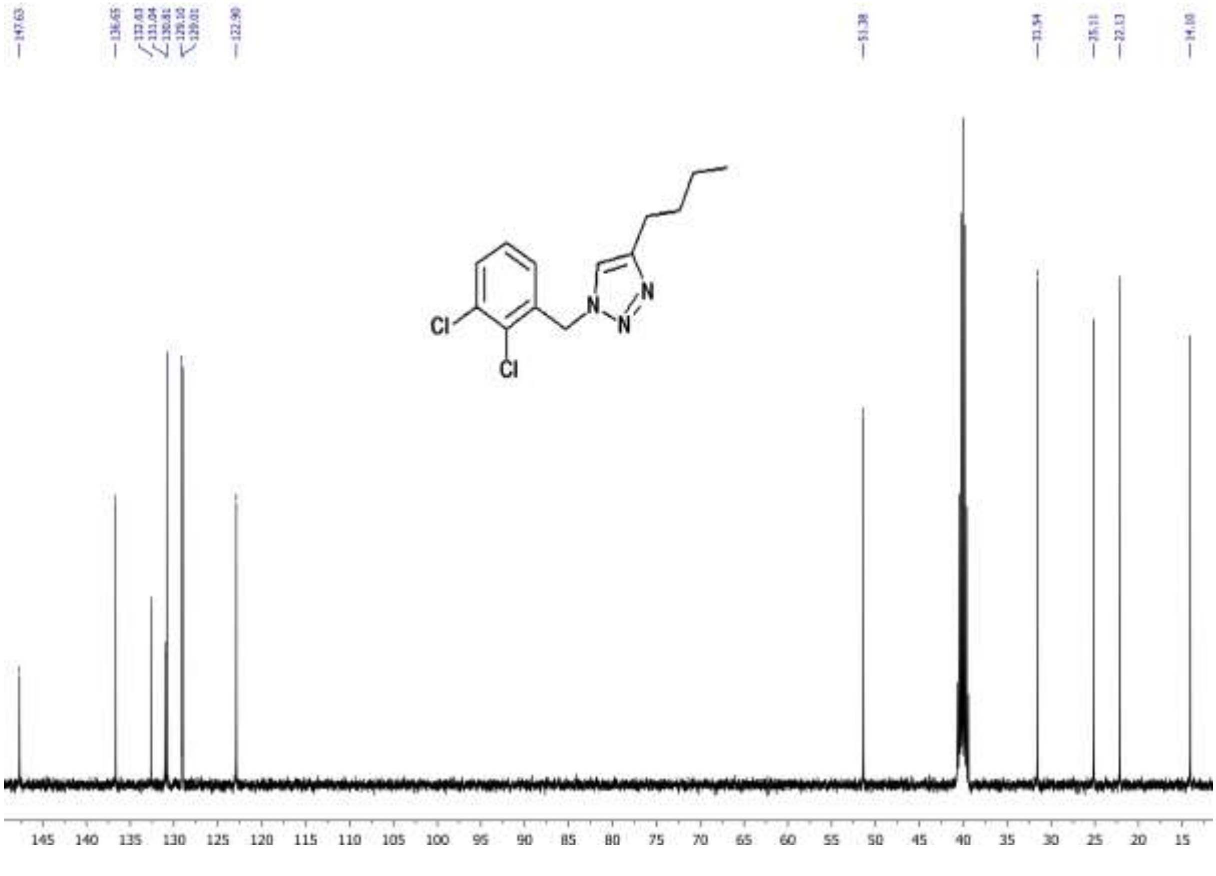

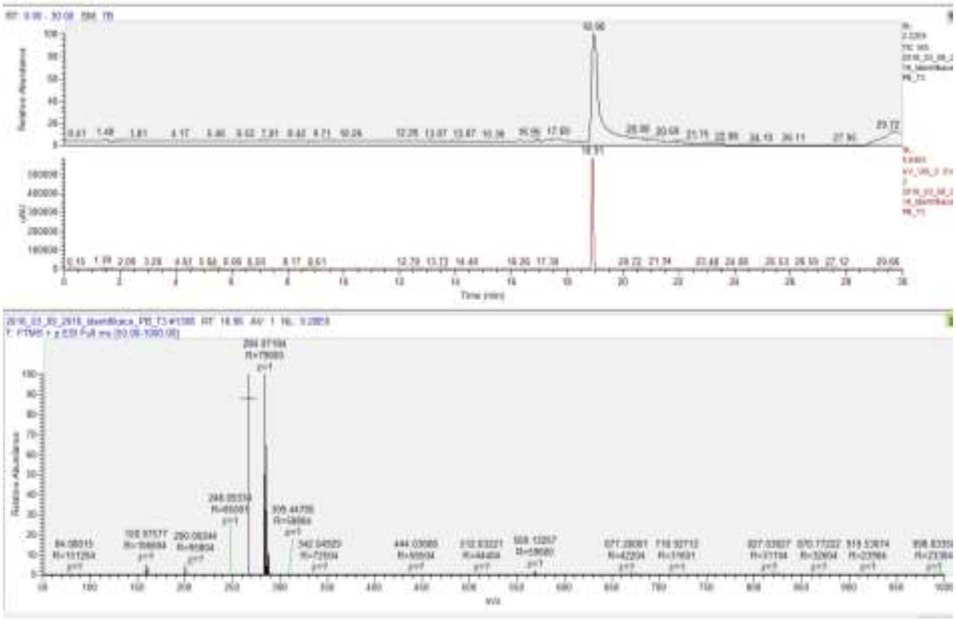

(1-benzyl-1H-1,2,3-triazol-4-yl)methanol (**1c**)

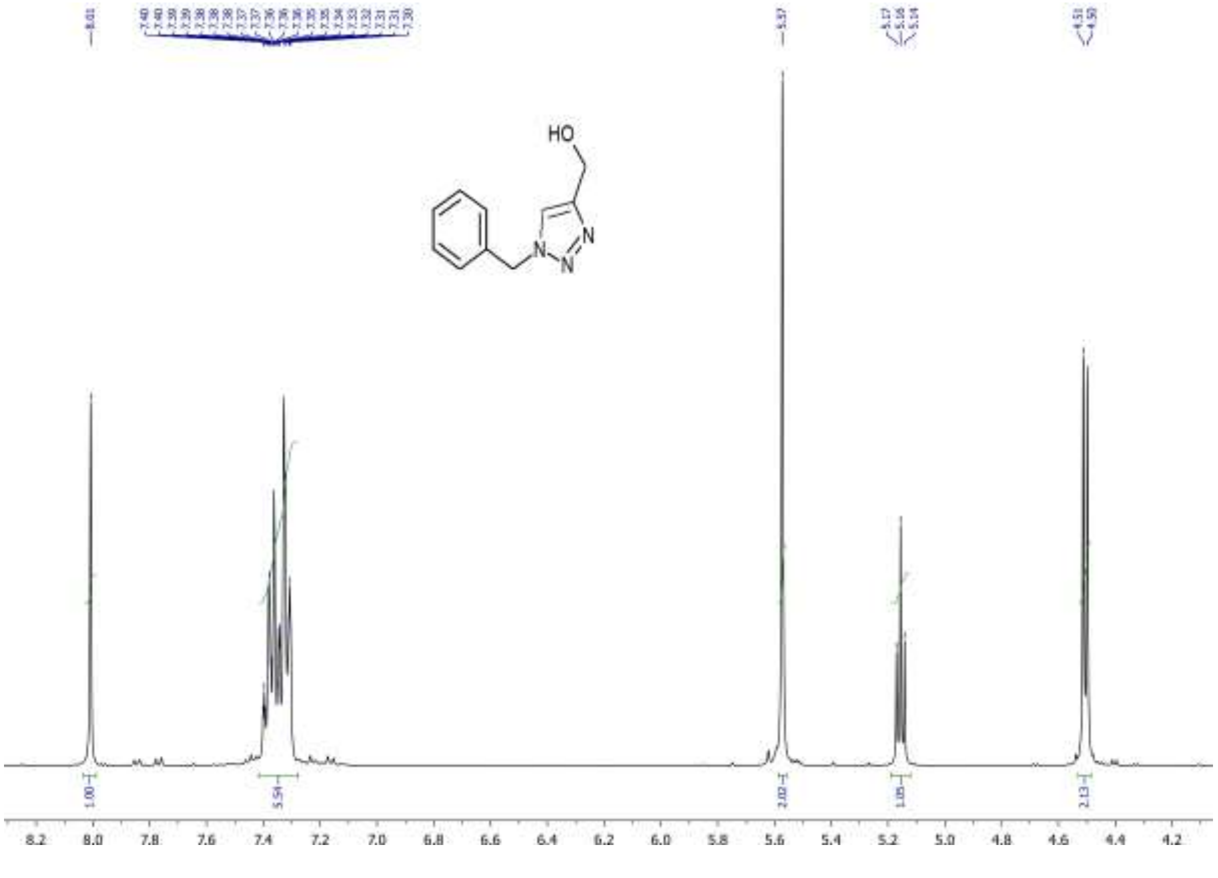

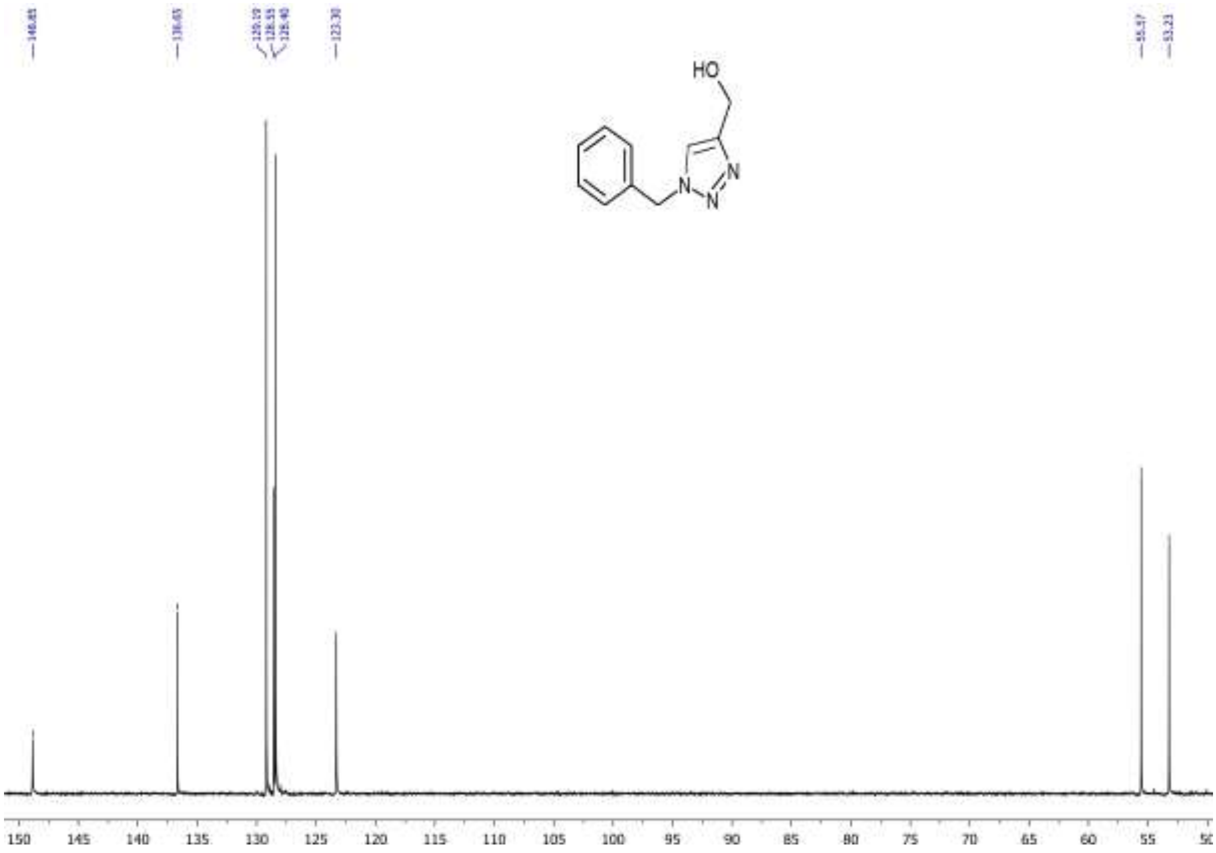

{1-[(4-bromophenyl)methyl]-1H-1,2,3-triazol-4-yl}methanol (**2c**)

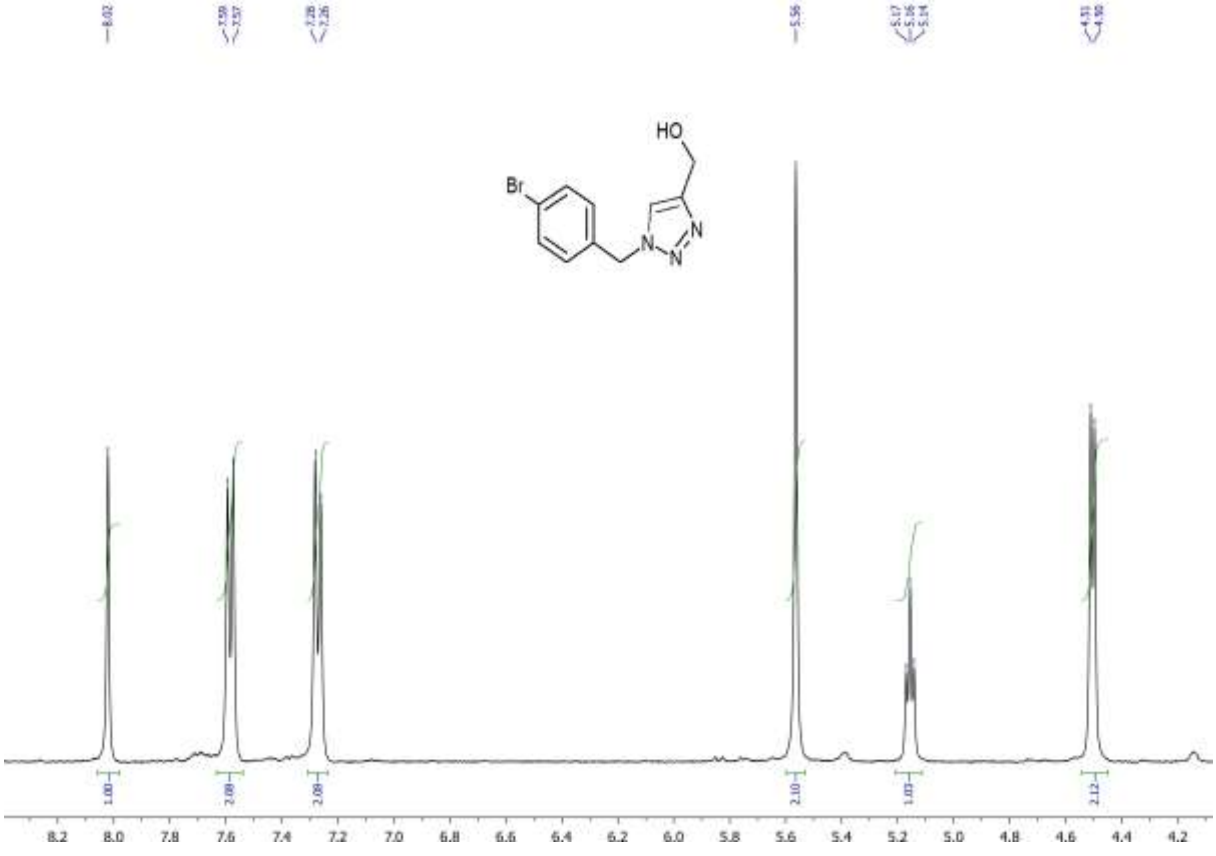

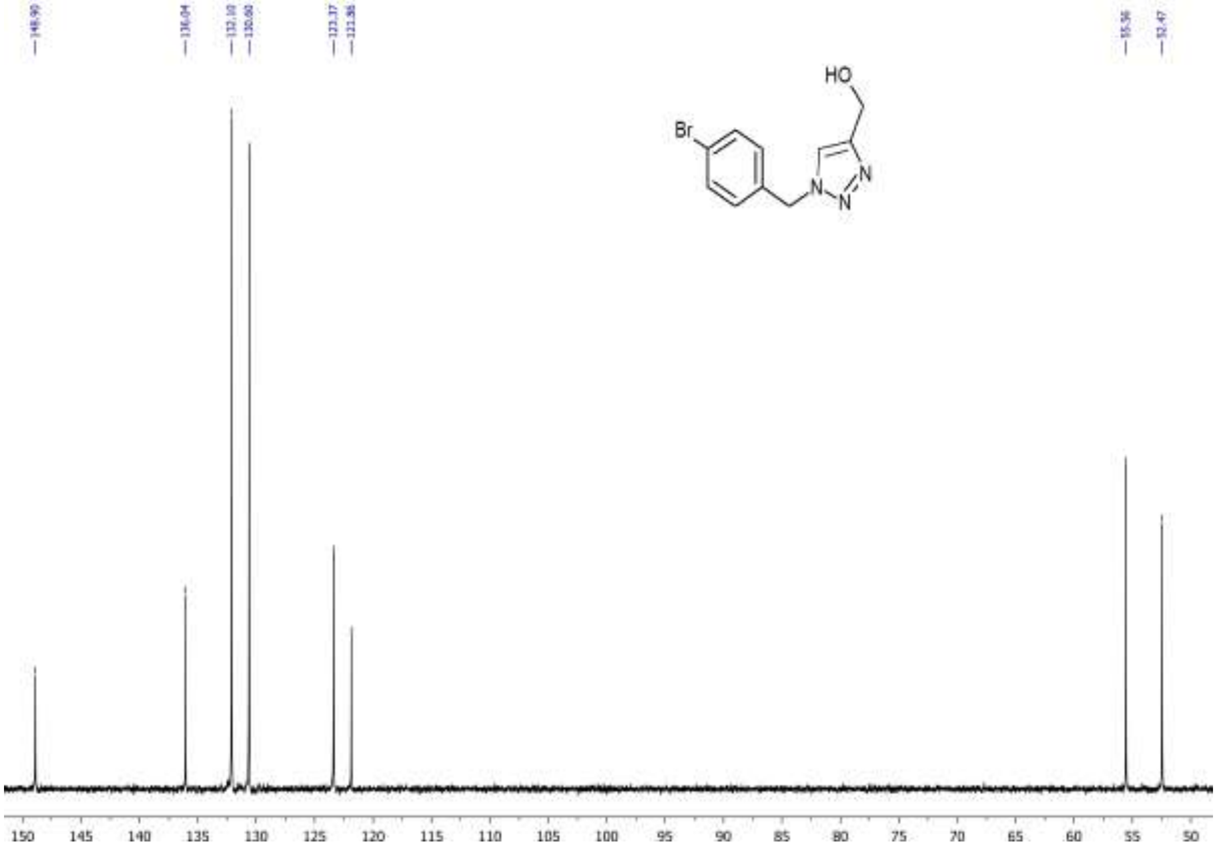

{1-[(4-chlorophenyl)methyl]-1H-1,2,3-triazol-4-yl}methanol (**3c**)

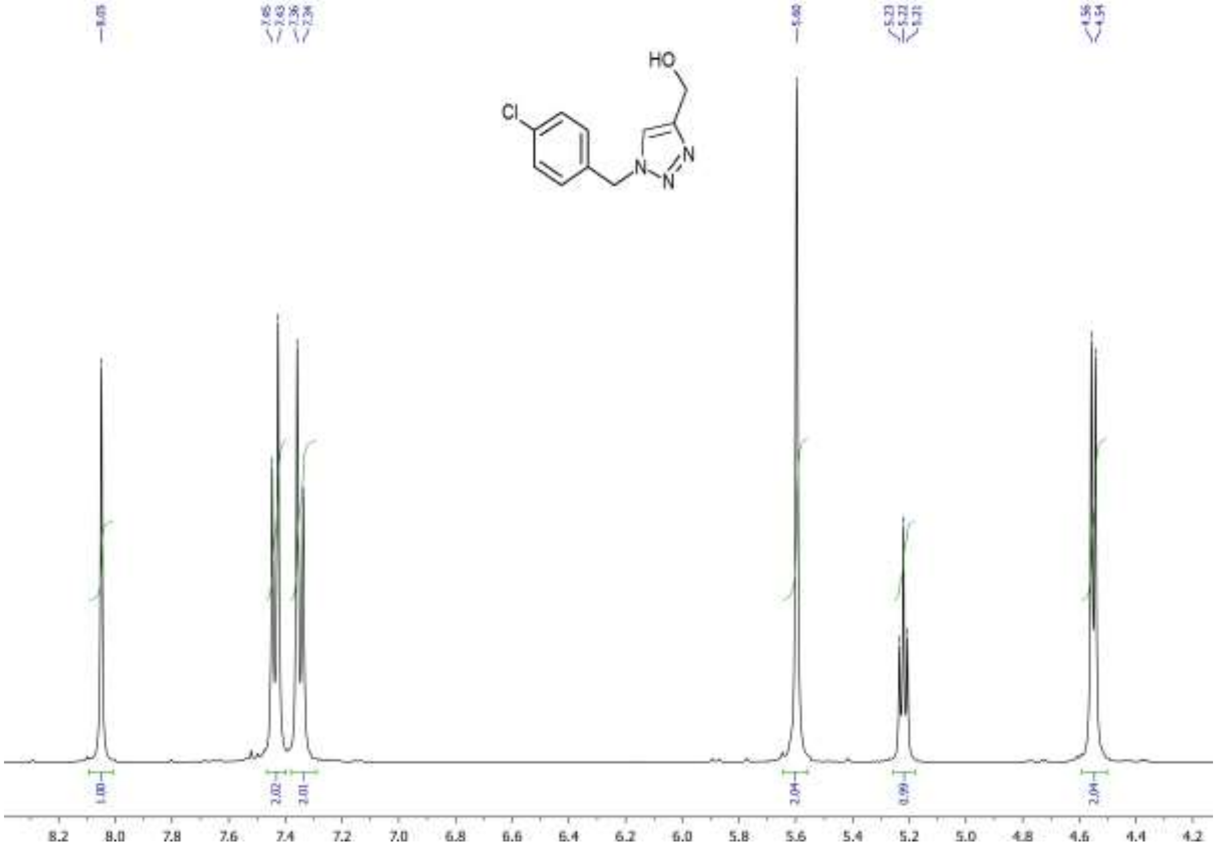

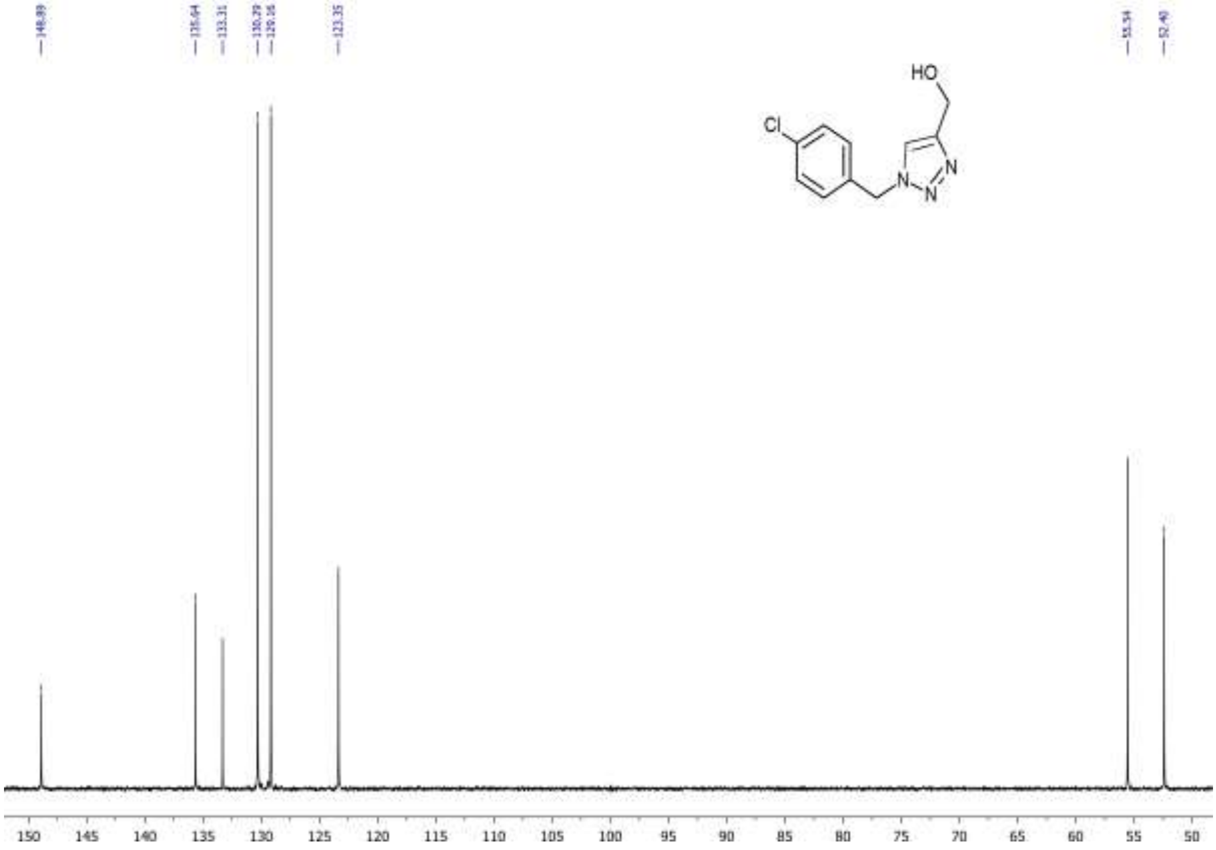
148.89
135.64
133.31
130.29
129.16
123.35
55.54
52.40
HO
Cl
N
150 145 140 135 130 125 120 115 110 105 100 95 90 85 80 75 70 65 60 55 50

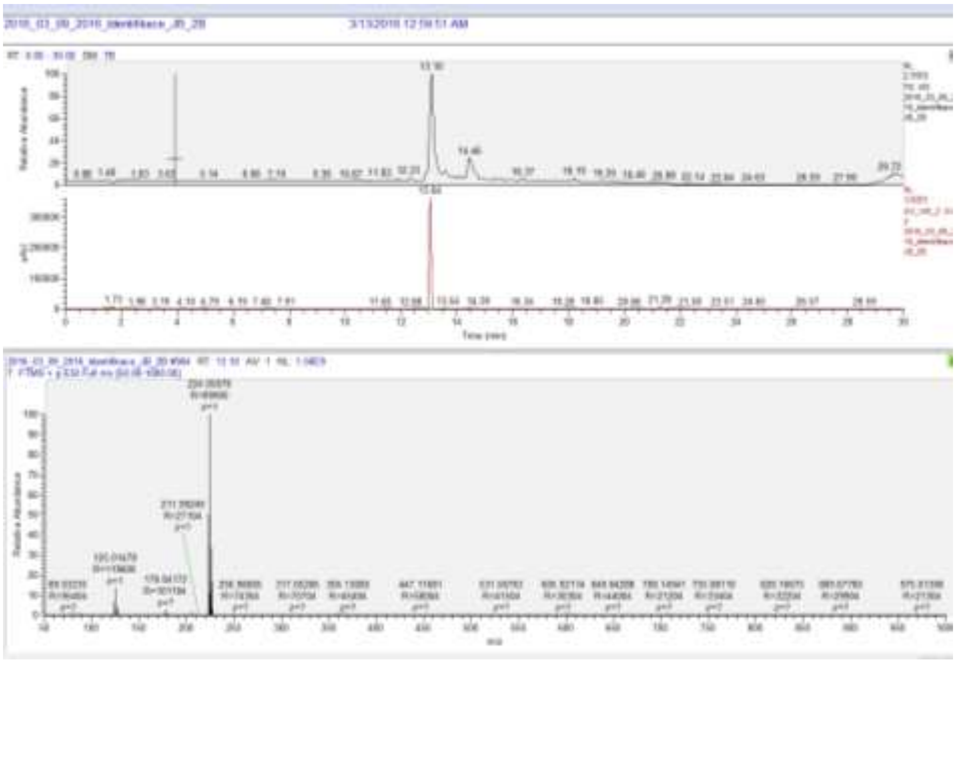

{1-[(4-iodophenyl)methyl]-1H-1,2,3-triazol-4-yl}methanol (**4c**)

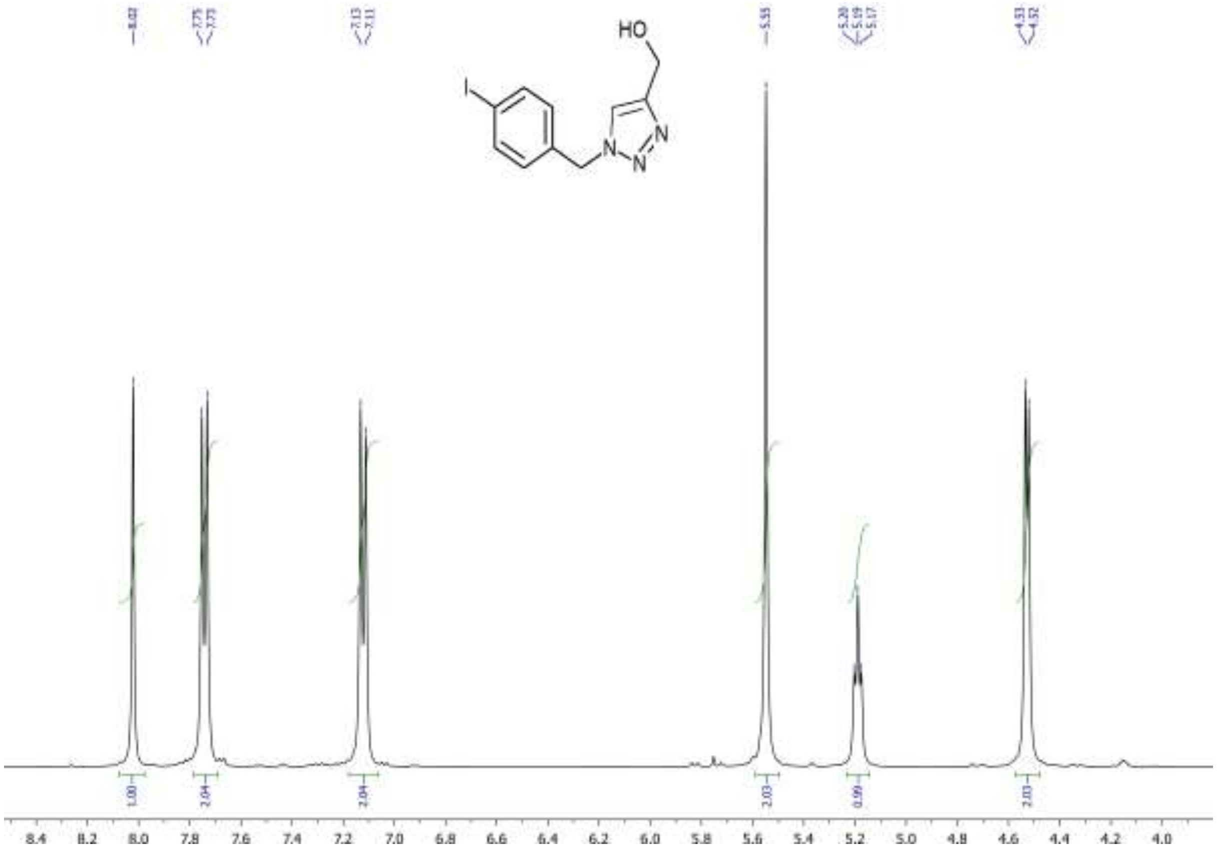

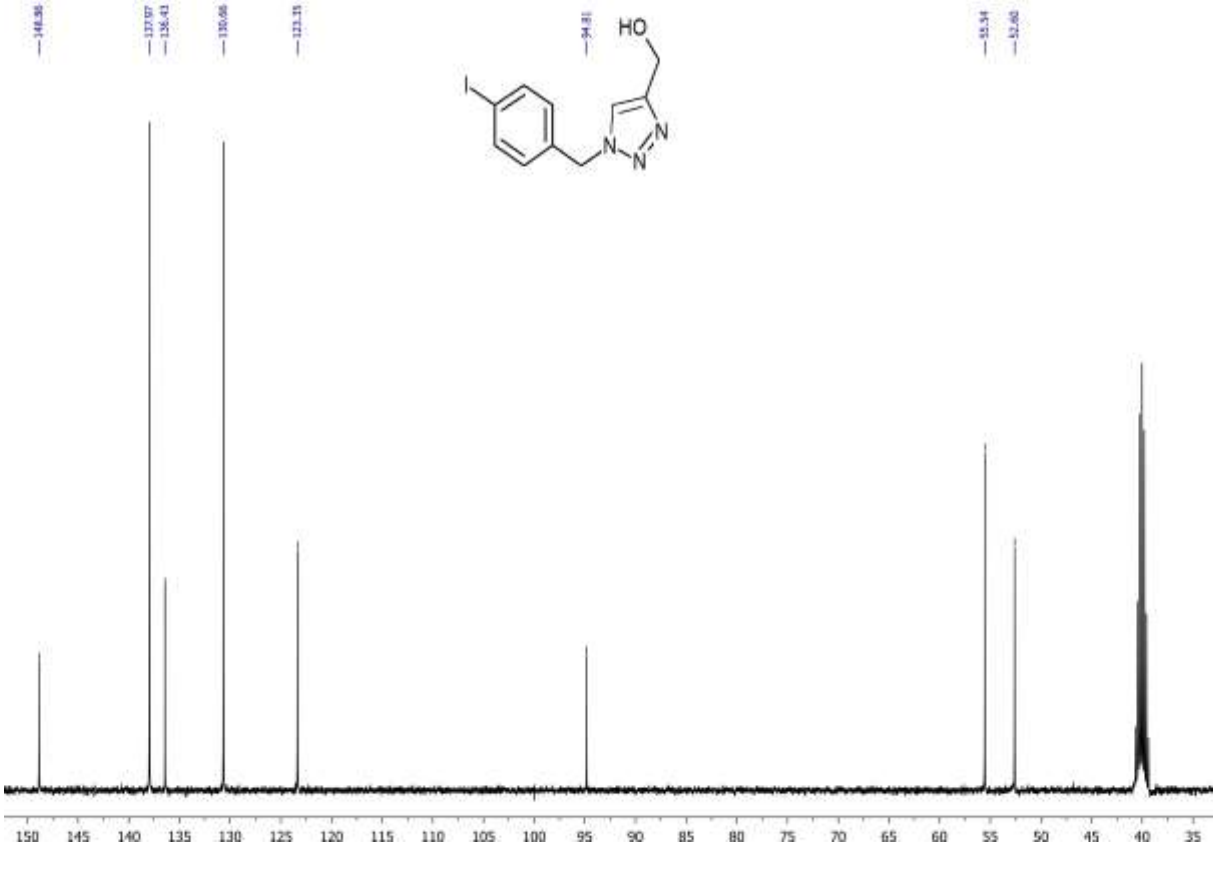

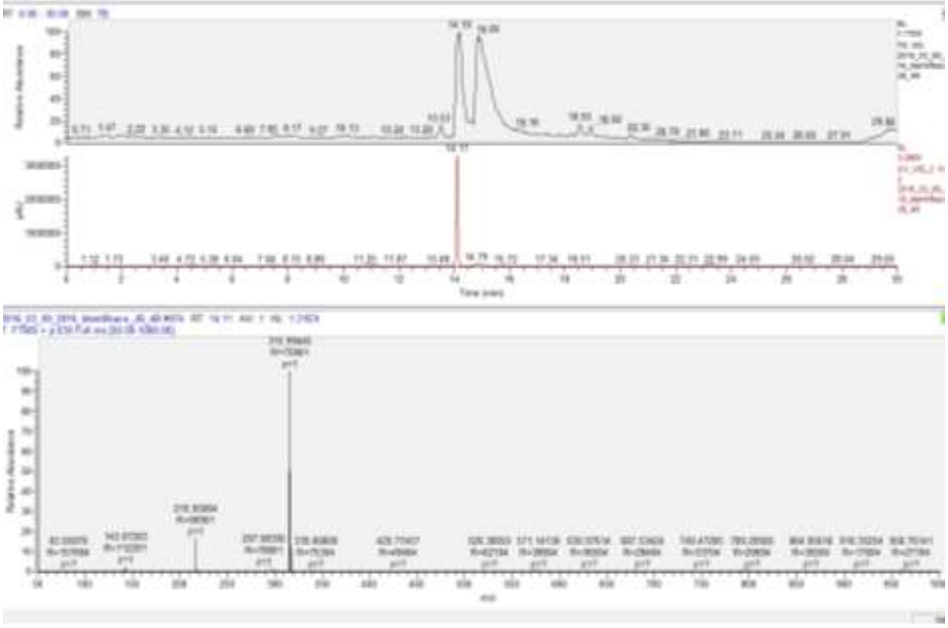

{1-[(2,3-dichlorophenyl)methyl]-1H-1,2,3-triazol-4-yl}methanol (**5c**)

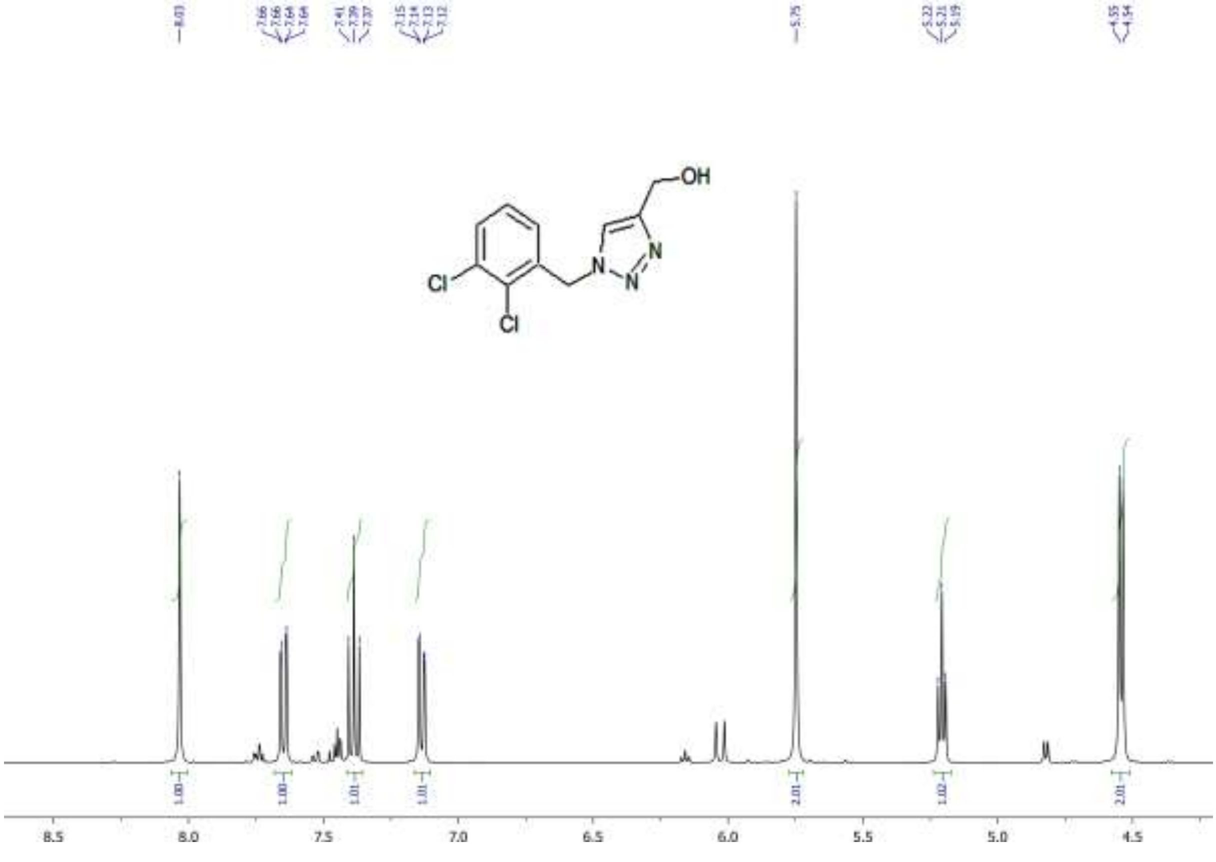

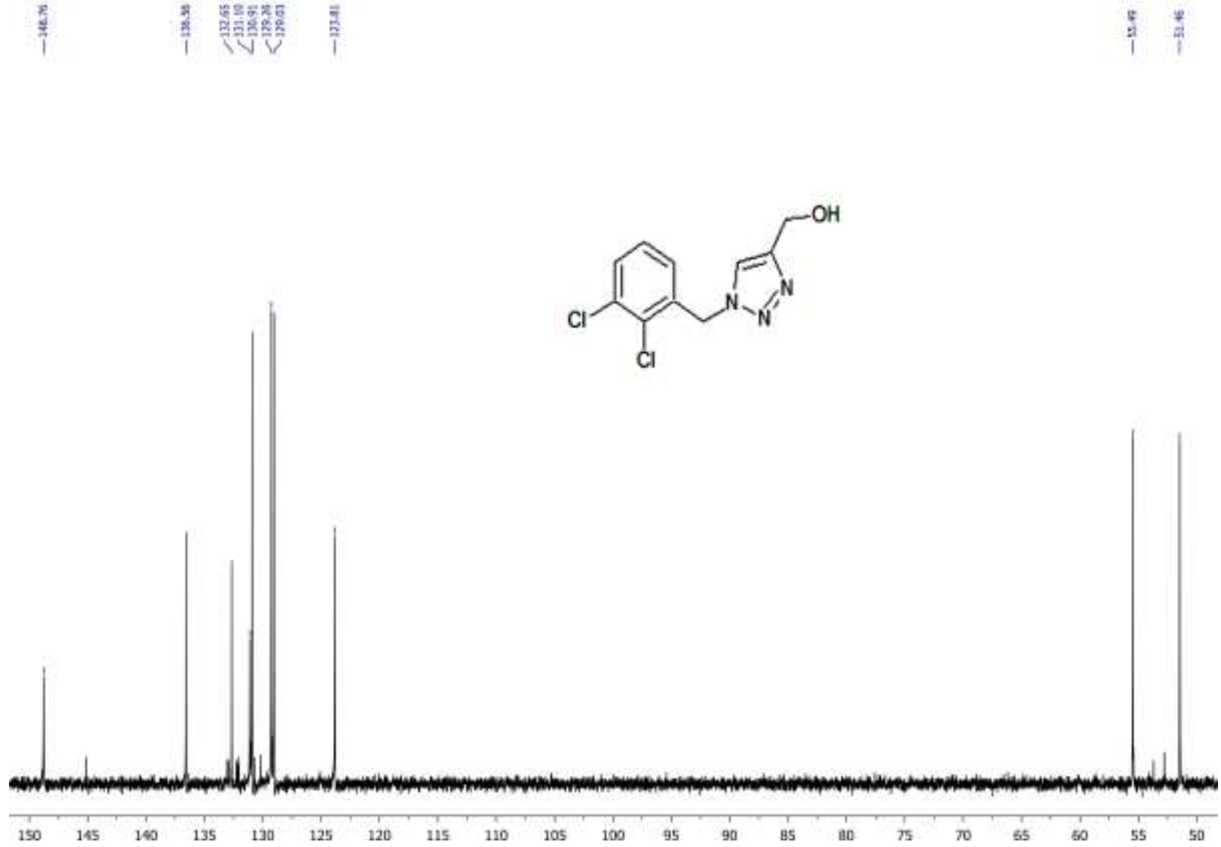
148.76
136.58
132.65
131.10
130.91
129.26
129.03
123.81
55.49
51.46
OH
N
Cl
150 145 140 135 130 125 120 115 110 105 100 95 90 85 80 75 70 65 60 55 50

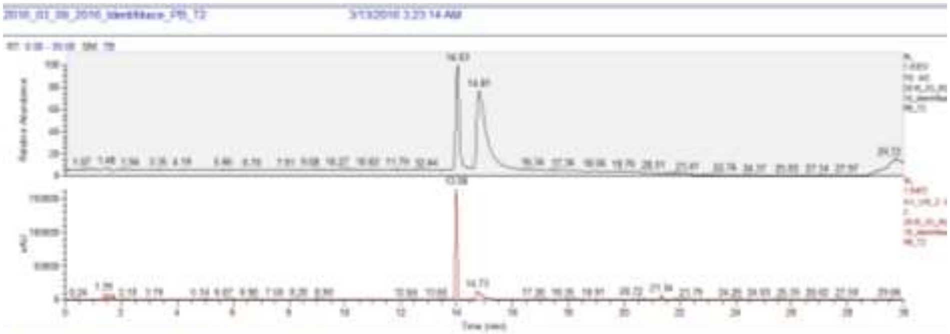

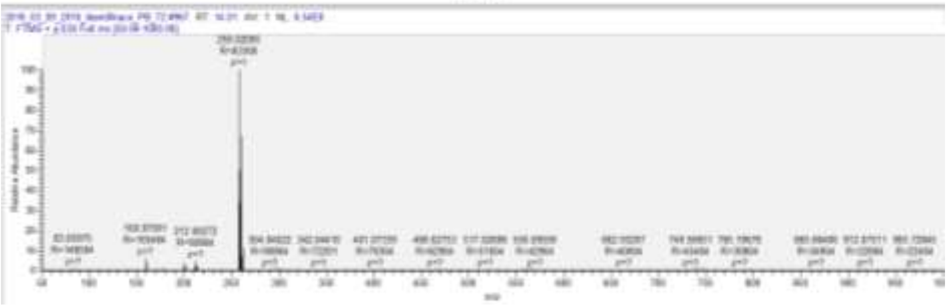

2-[(4-phenyl-1H-1,2,3-triazol-1-yl)methyl]quinoline (**6a**)

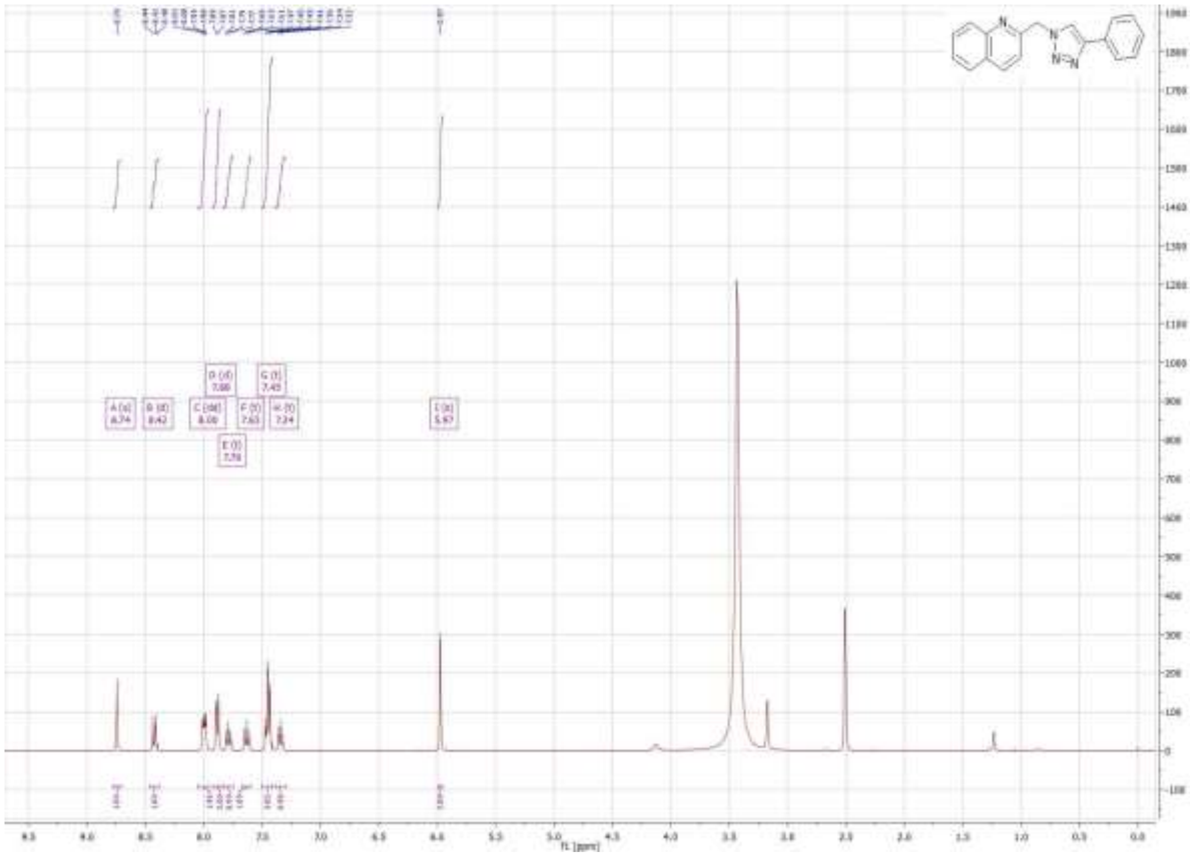

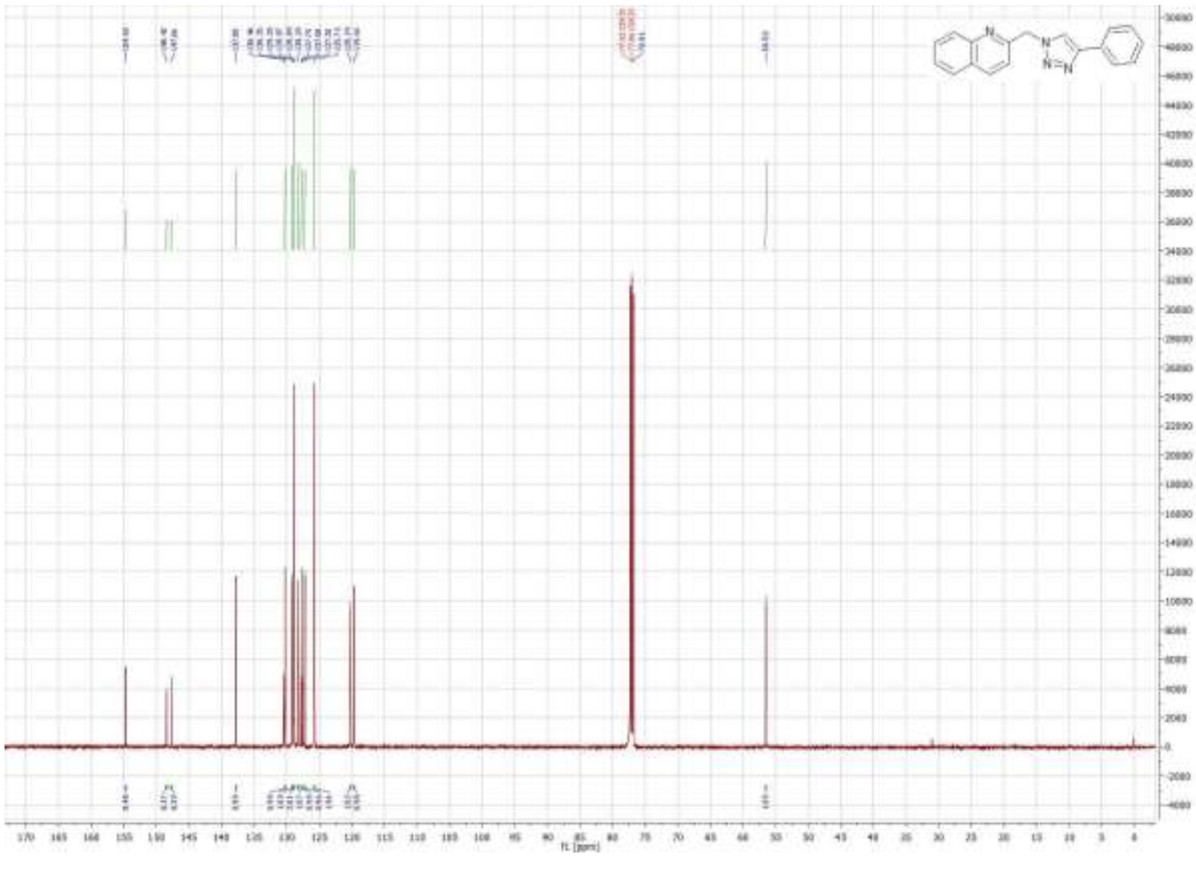

2-[(4-butyl-1H-1,2,3-triazol-1-yl)methyl]quinoline (**6b**)

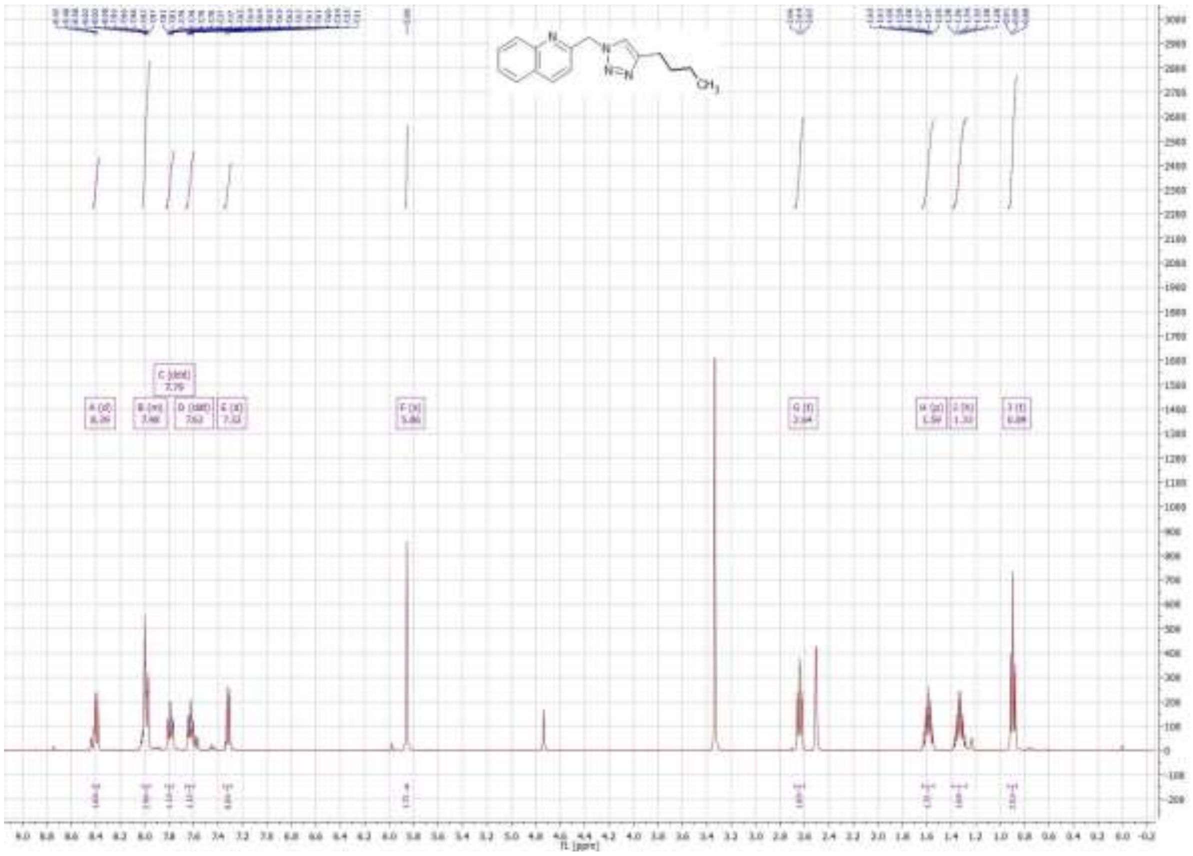

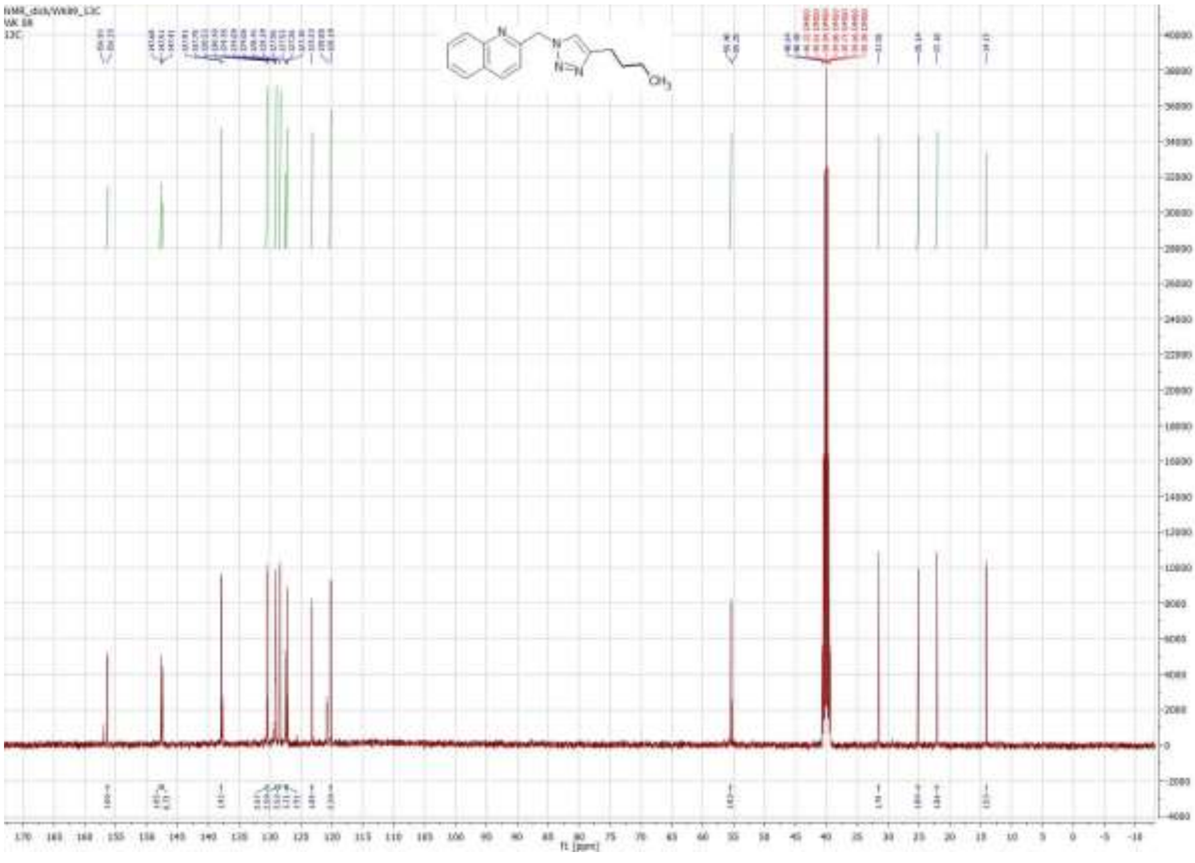

{1-[(quinolin-2-yl)methyl]-1H-1,2,3-triazol-4-yl}methanol (**6c**)

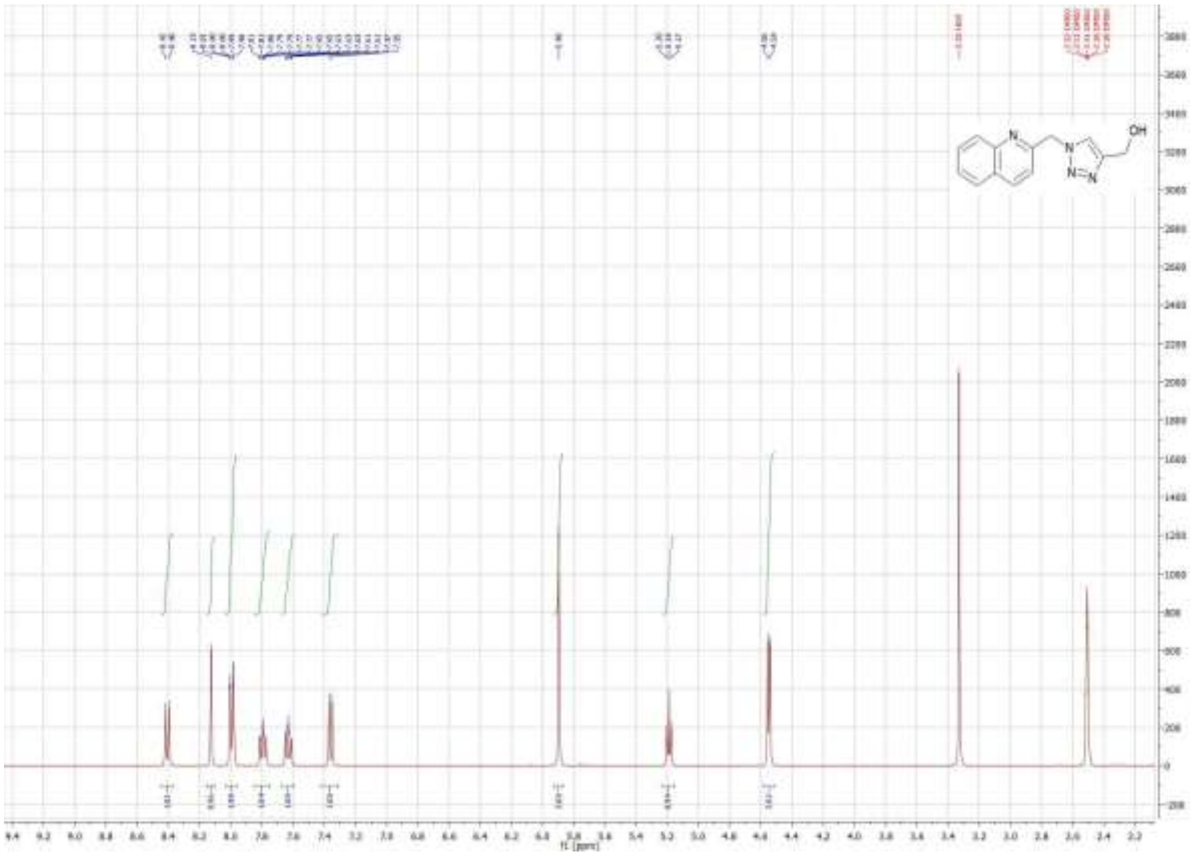

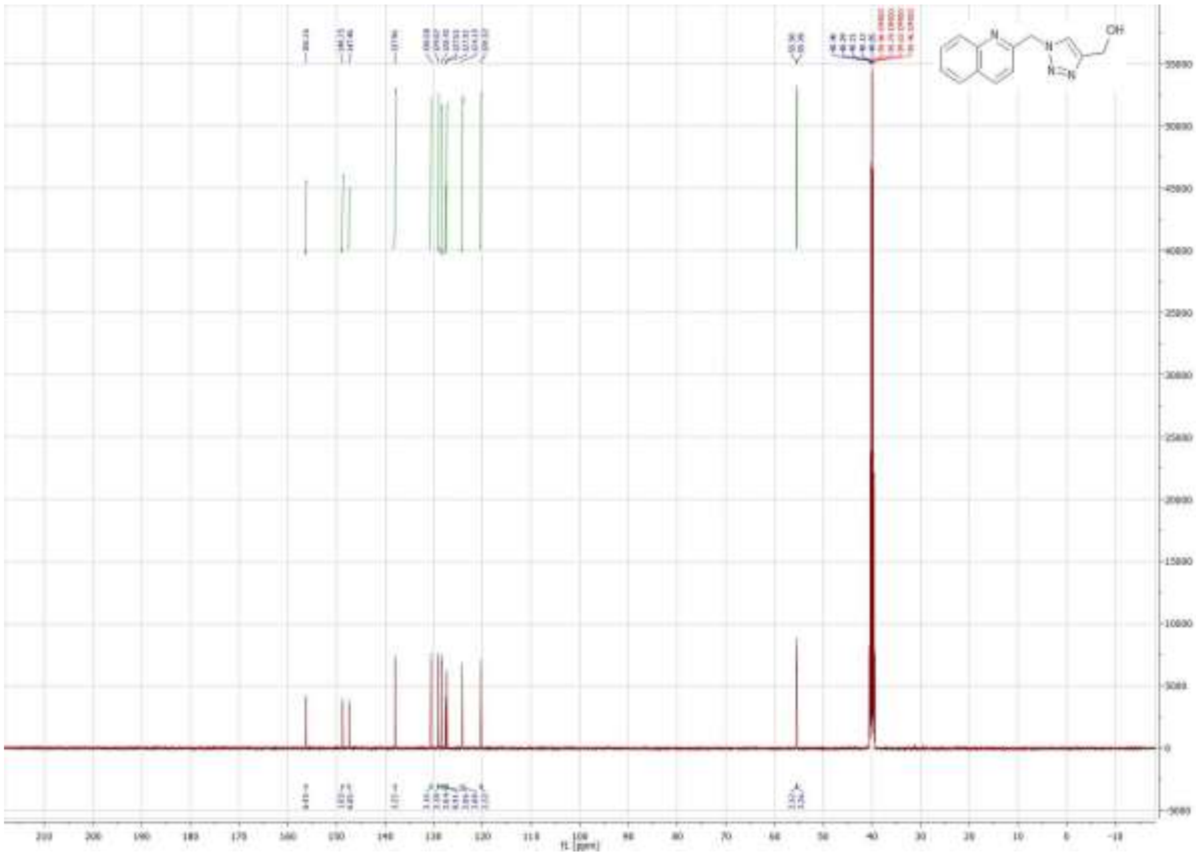

8-chloro-2-[(4-phenyl-1H-1,2,3-triazol-1-yl)methyl]quinoline (**7a**)

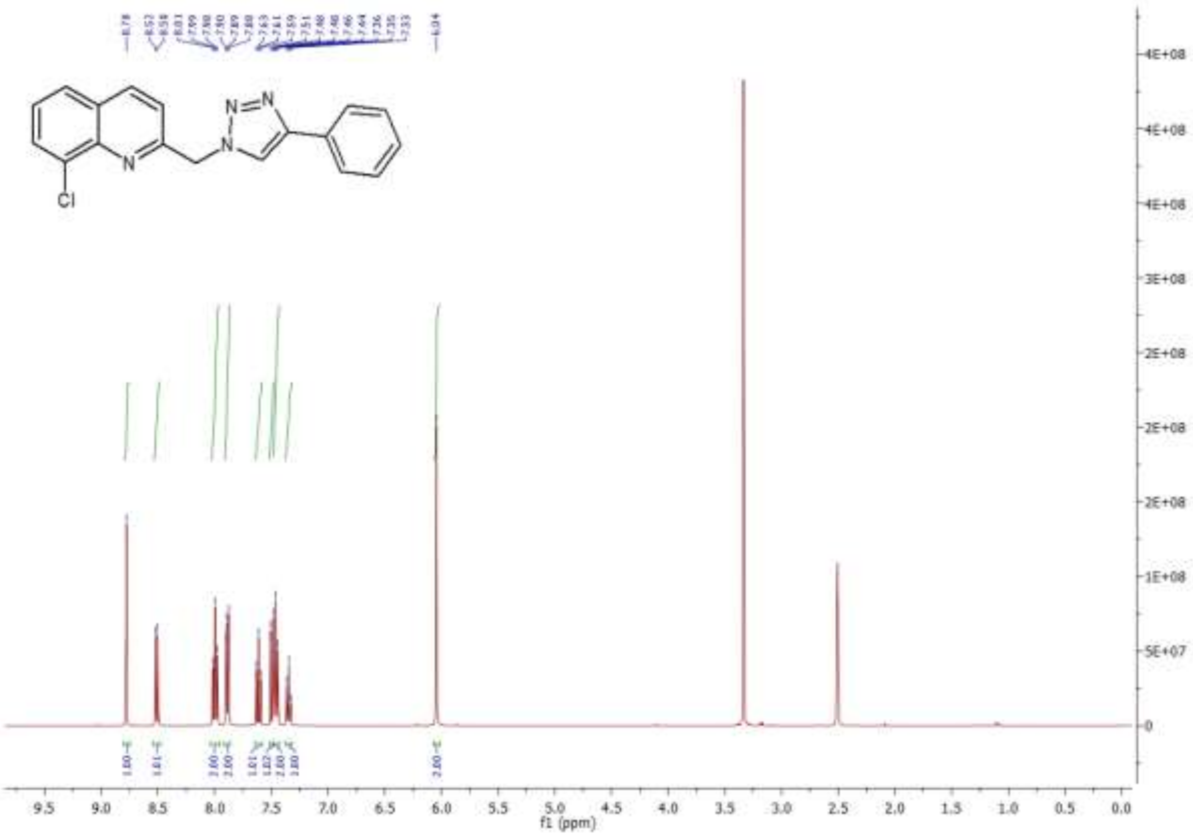

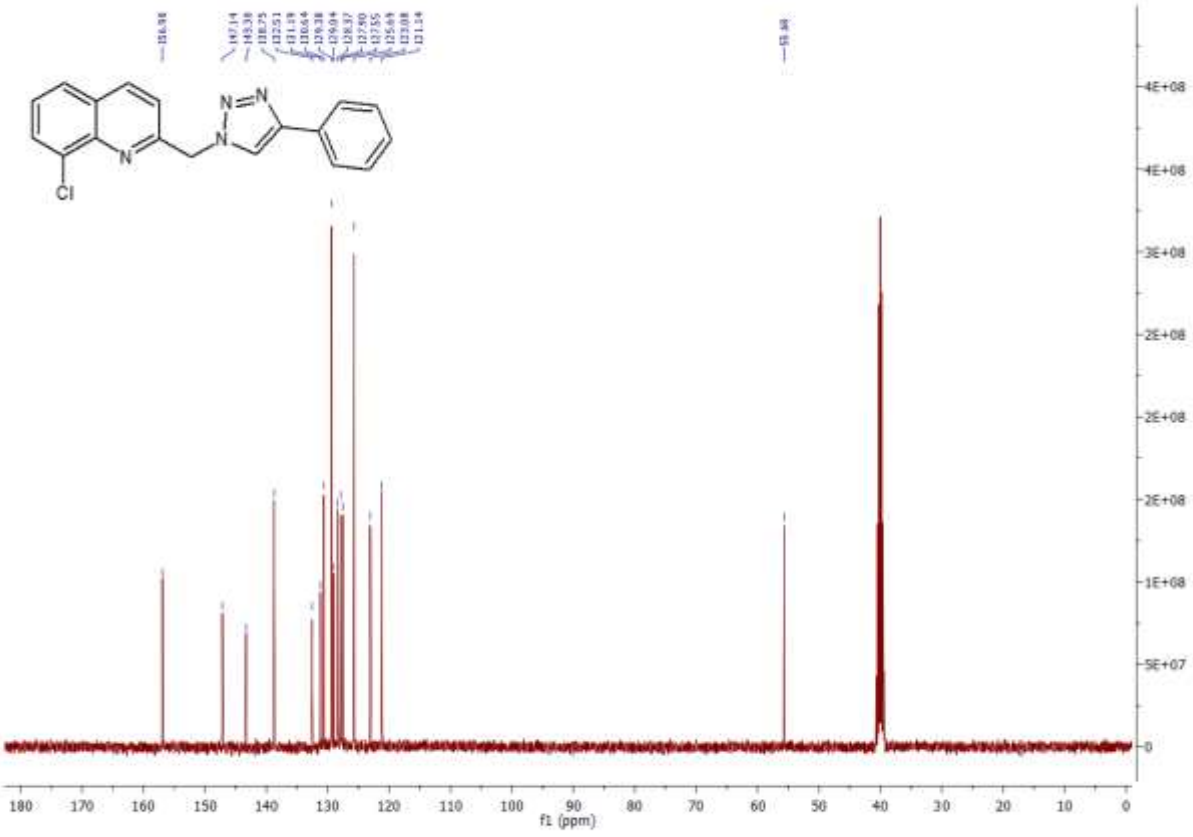

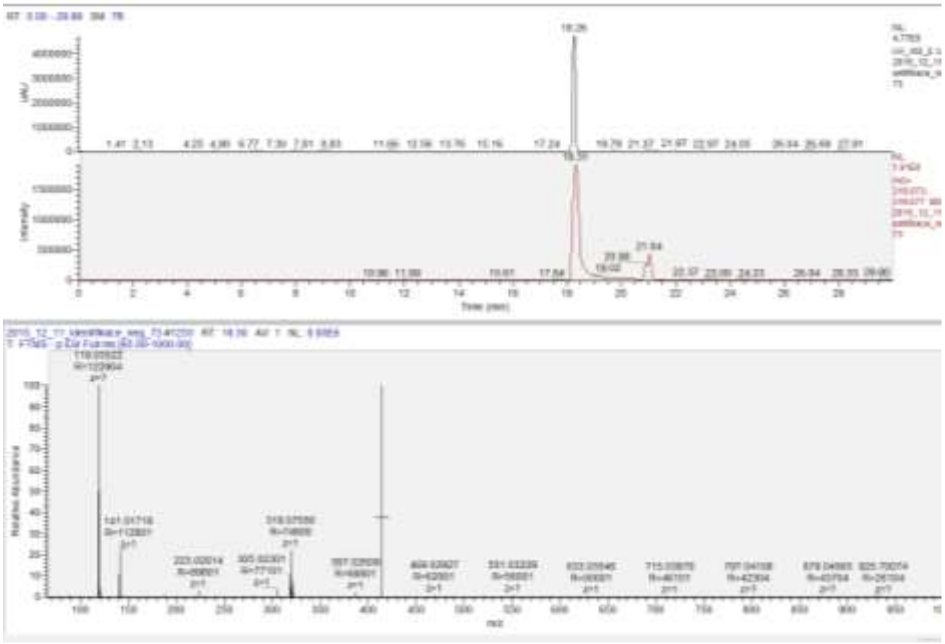

2-[(4-butyl-1H-1,2,3-triazol-1-yl)methyl]-8-chloroquinoline (**7b**)

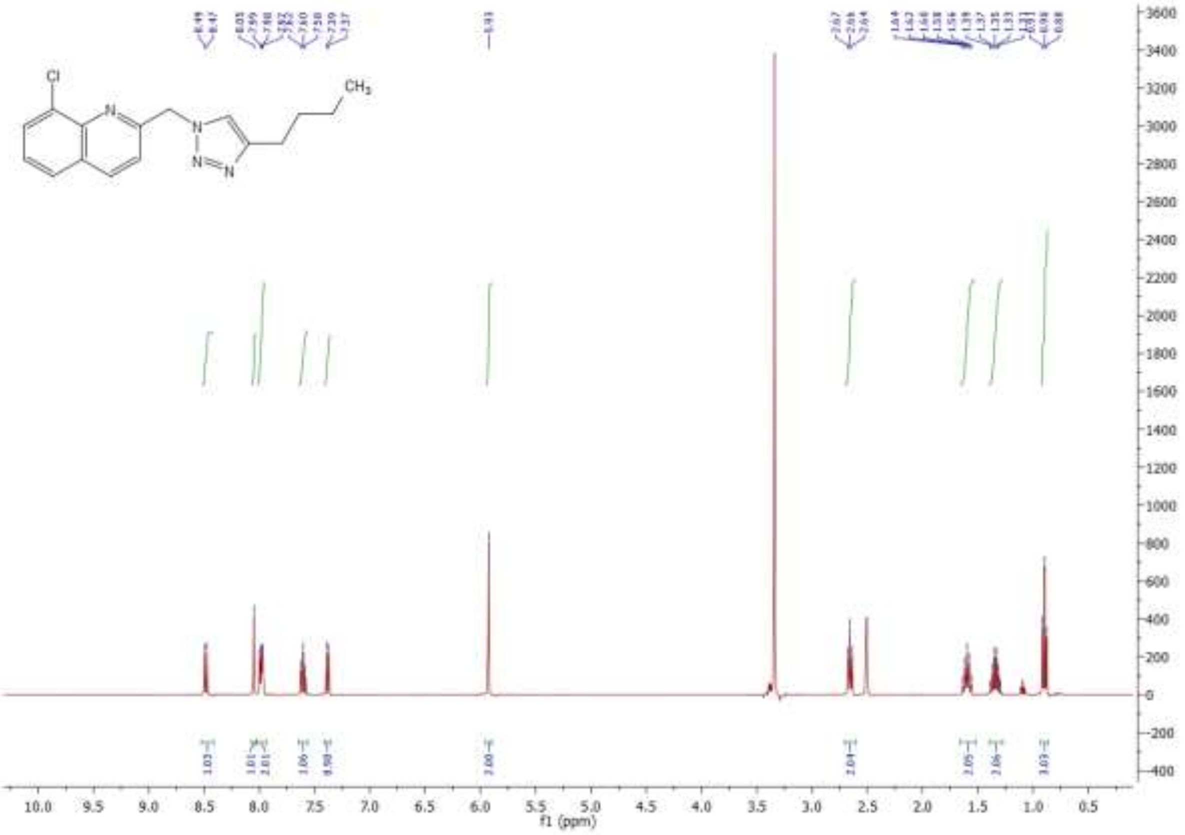

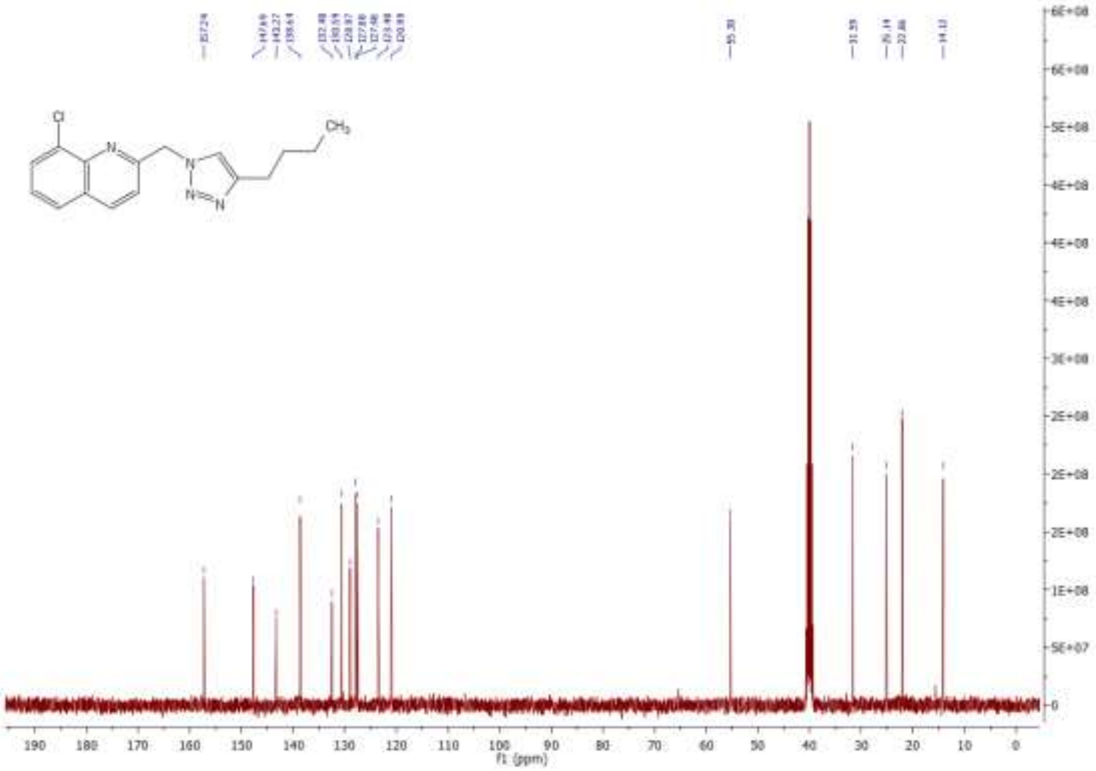

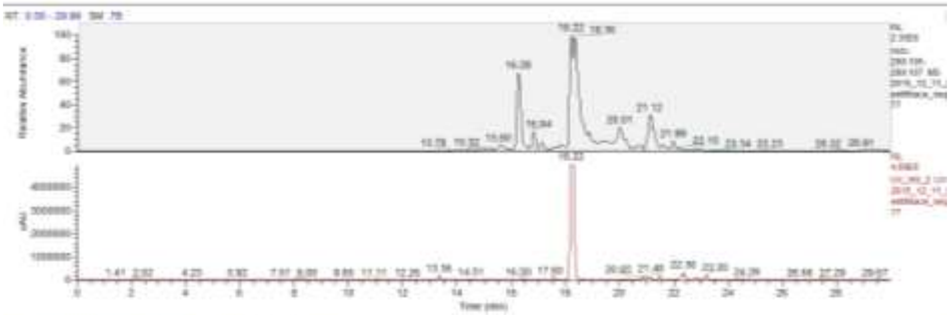

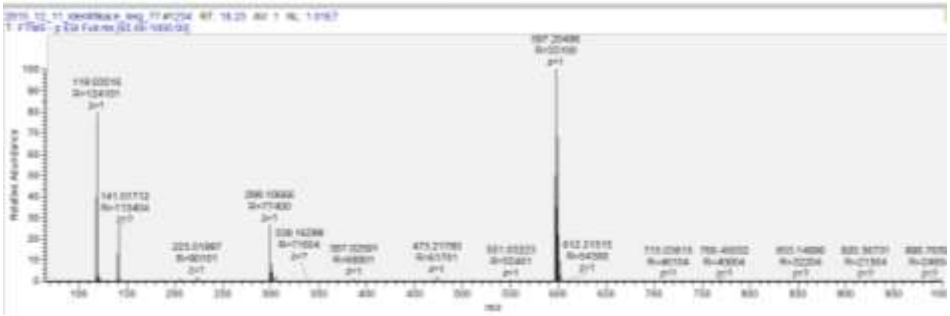

2-[(4-phenyl-1H-1,2,3-triazol-1-yl)methyl]quinazolin-4(3H)-one (**8a**)

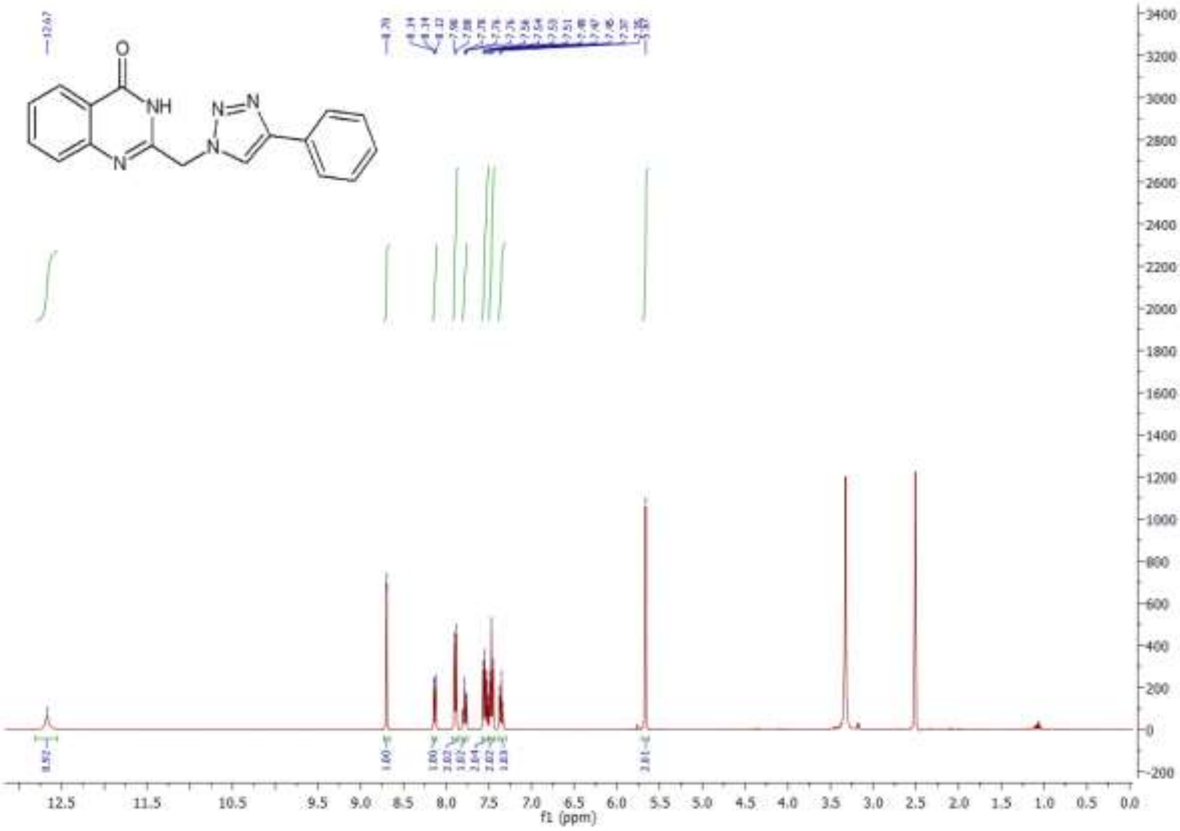
O
NH
N
N=N
N
12.5
11.5
10.5
9.5
9.0
8.5
8.0
7.5
7.0
6.5
6.0
5.5
5.0
4.5
4.0
3.5
3.0
2.5
2.0
1.5
1.0
0.5
0.0
f1 (ppm)

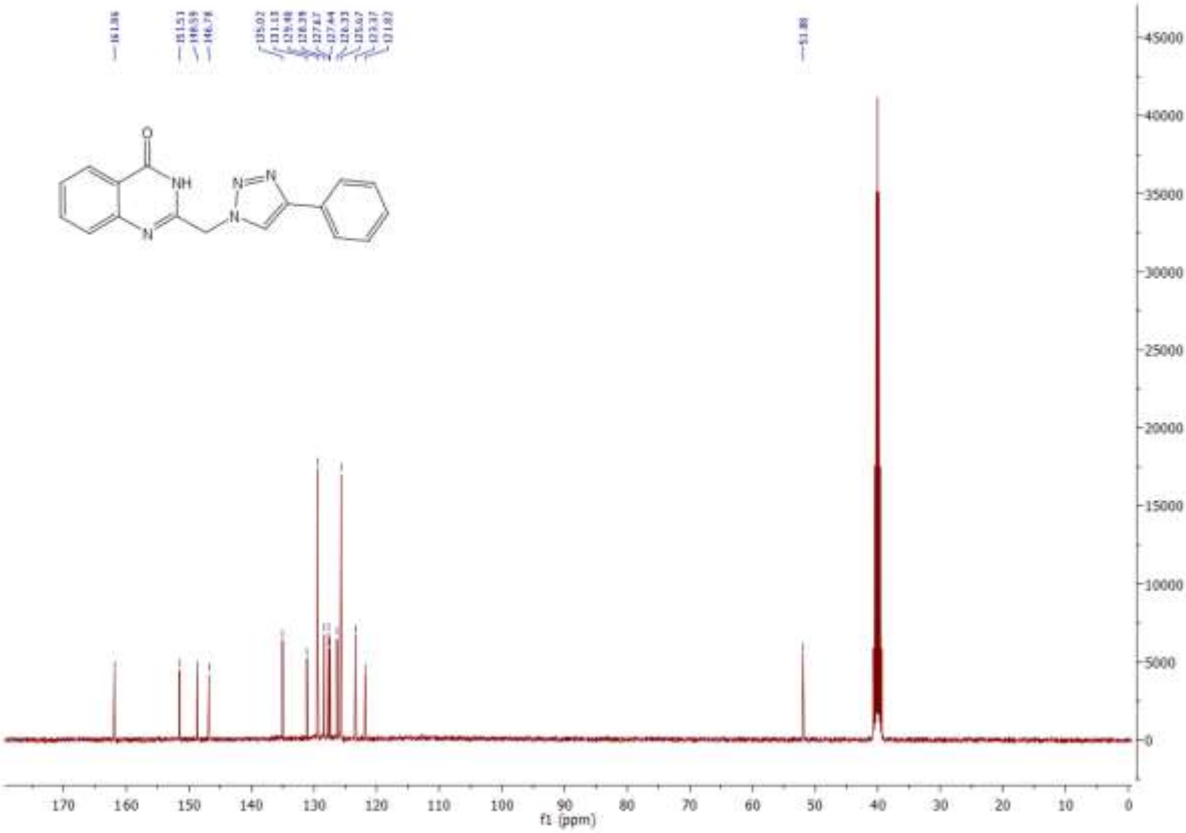
161.86
151.51
148.59
146.78
135.02
131.13
129.48
128.39
127.47
127.44
126.33
125.67
123.37
121.92
51.80
O
NH
N
N=N
N
f1 (ppm)

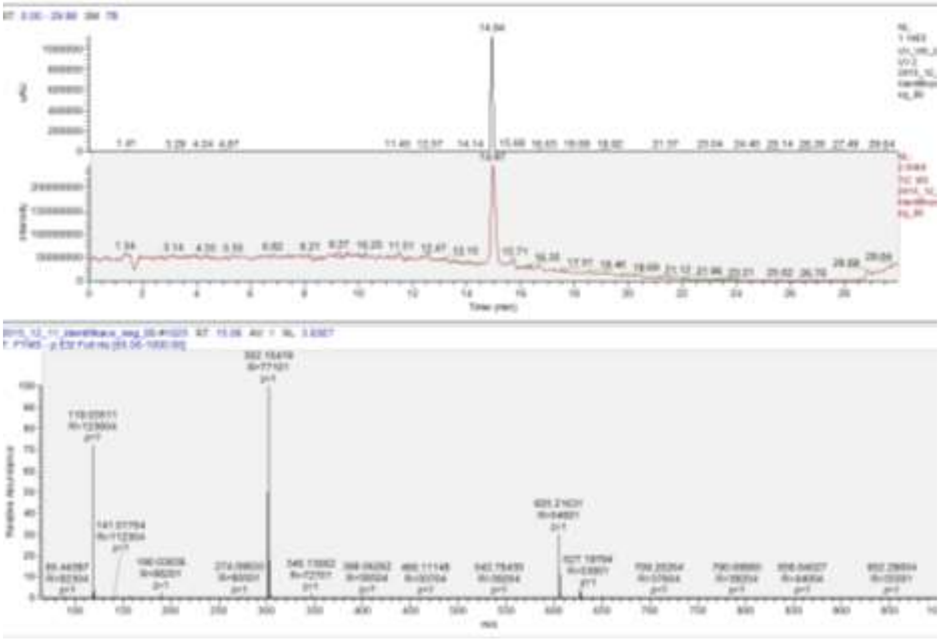

{1-[(8-chloroquinolin-2-yl)methyl]-1H-1,2,3-triazol-4-yl}methanol (**7c**)

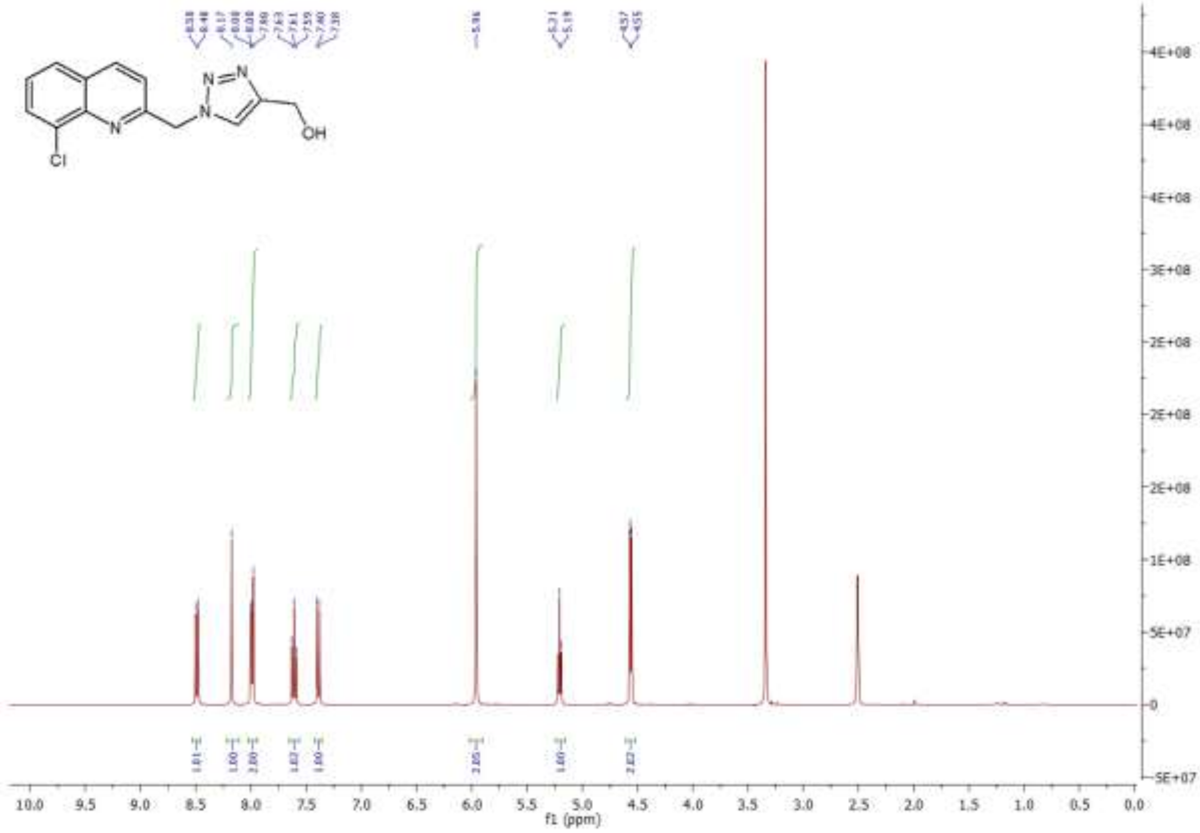

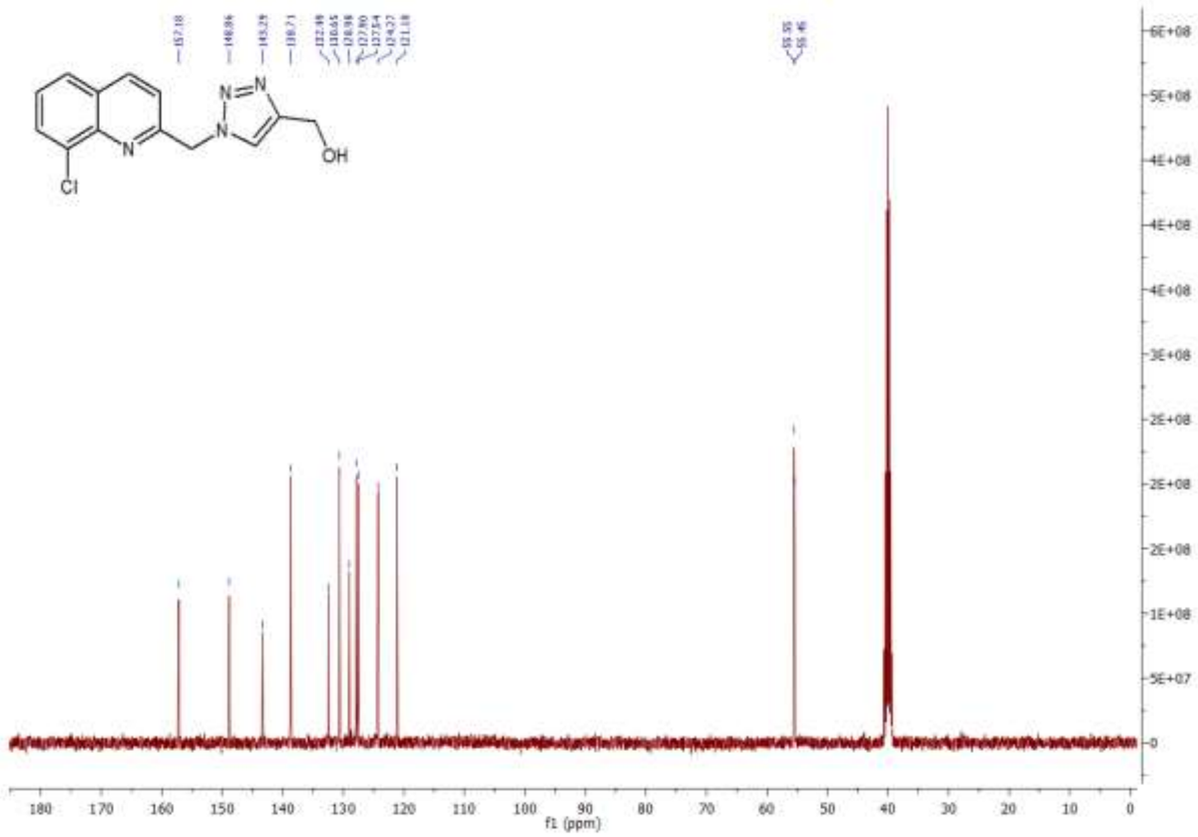
157.18
148.86
143.29
138.71
132.49
130.65
128.98
127.80
127.54
124.27
121.18
55.55
55.46
f1 (ppm)

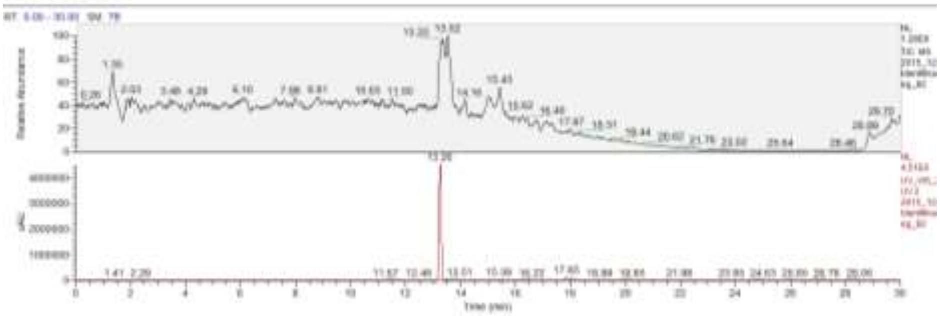

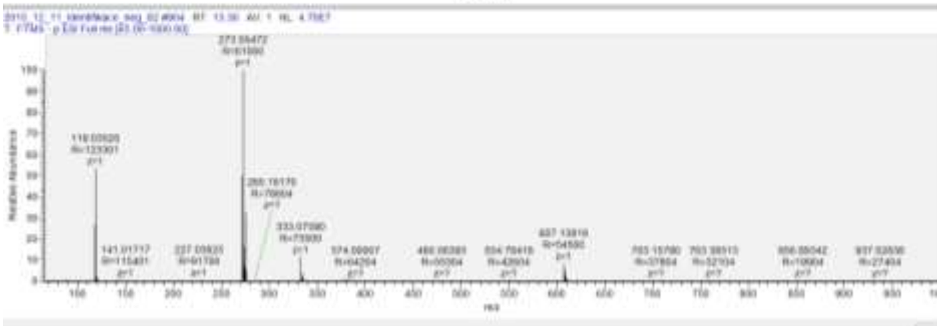

1-{1-[(8-chloroquinolin-2-yl)methyl]-1H-1,2,3-triazol-4-yl}ethan-1-ol (**7d**)

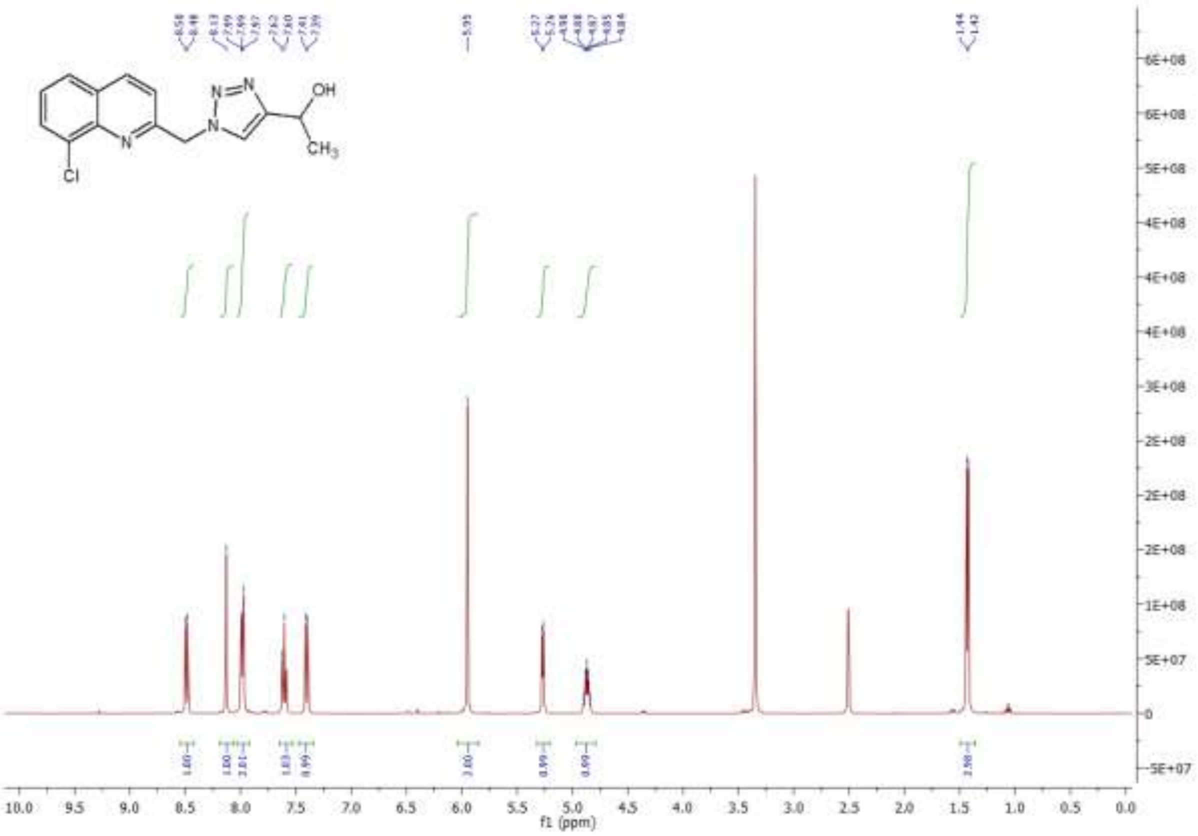

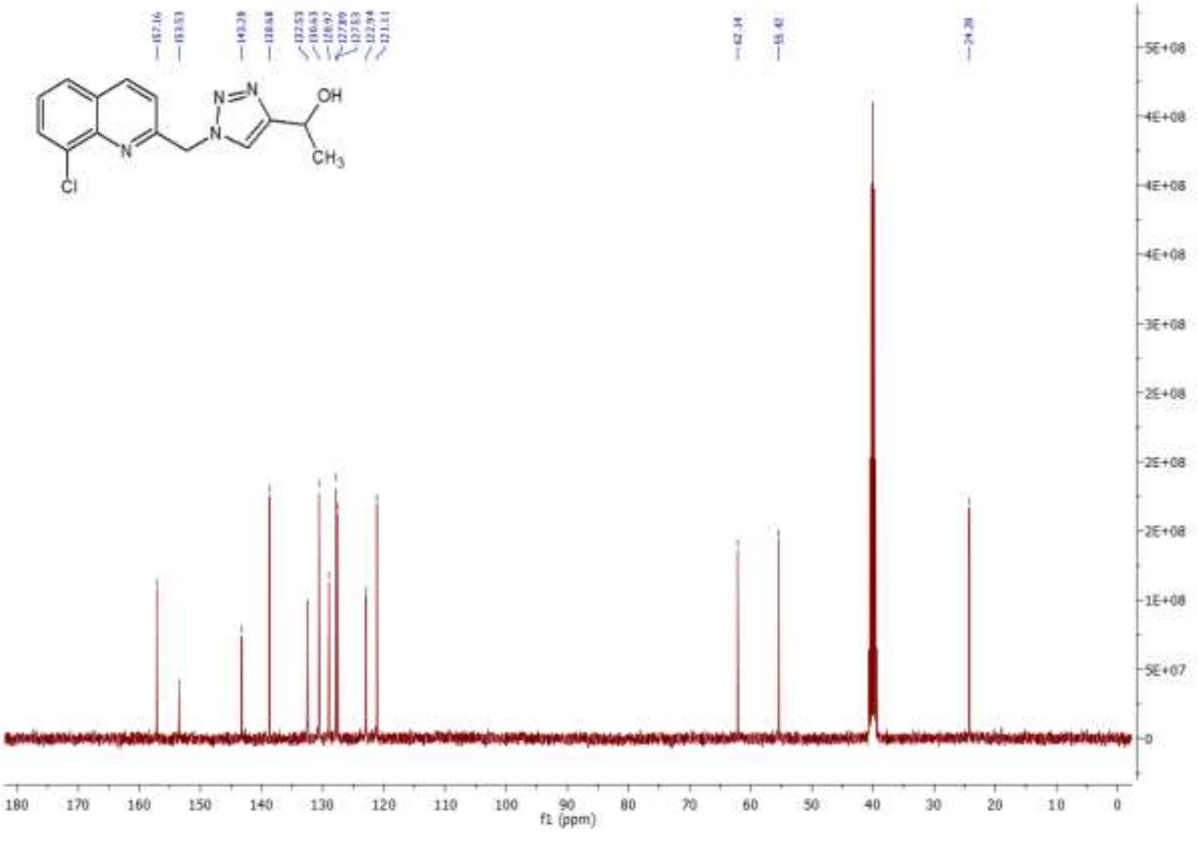

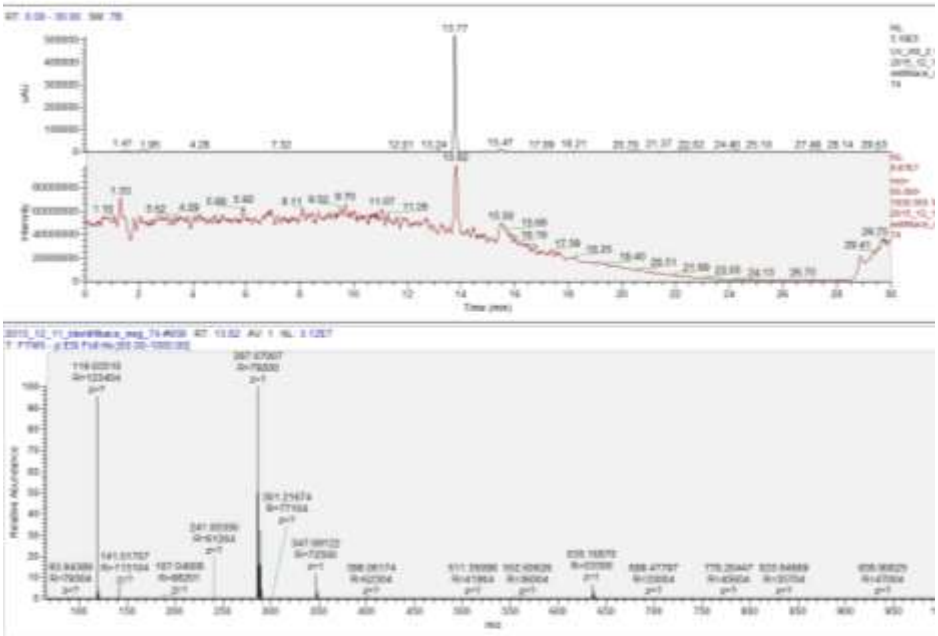

2-({4-[(benzyloxy)methyl]-1H-1,2,3-triazol-1-yl}methyl)-8-chloroquinoline (**7e**)

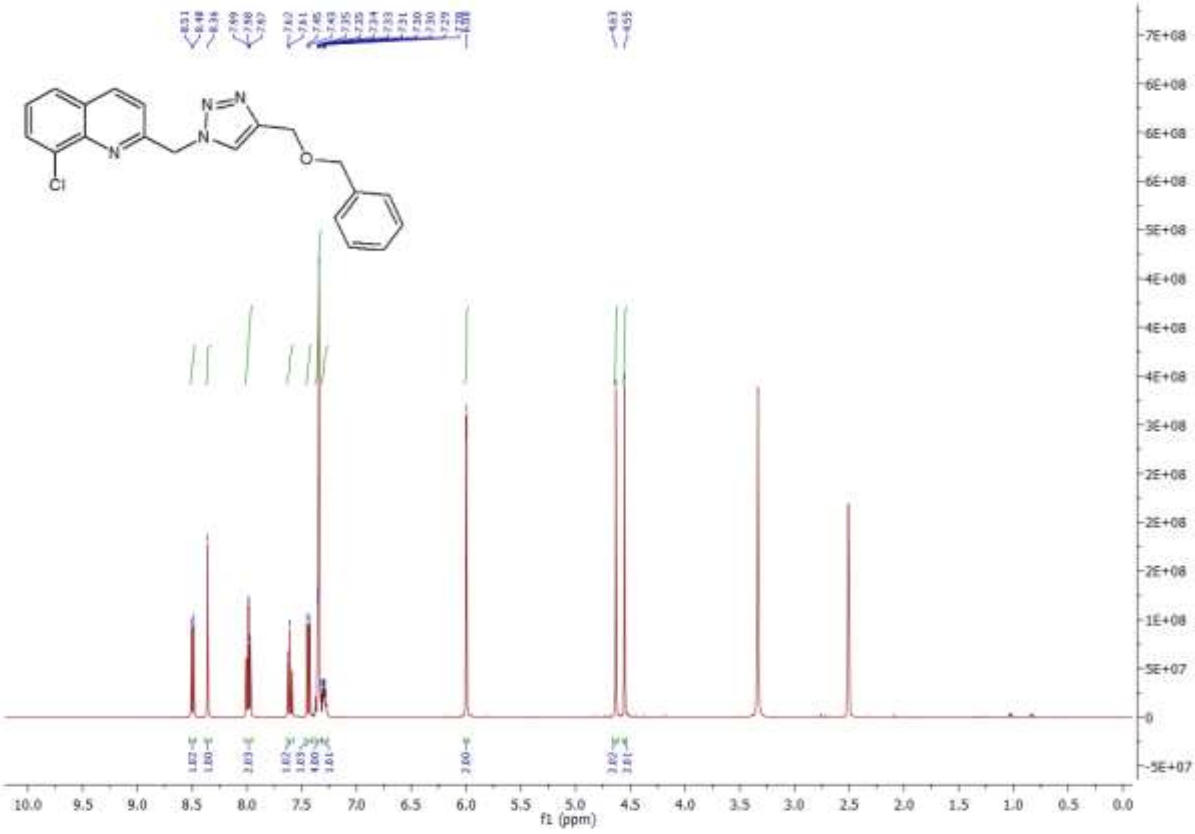

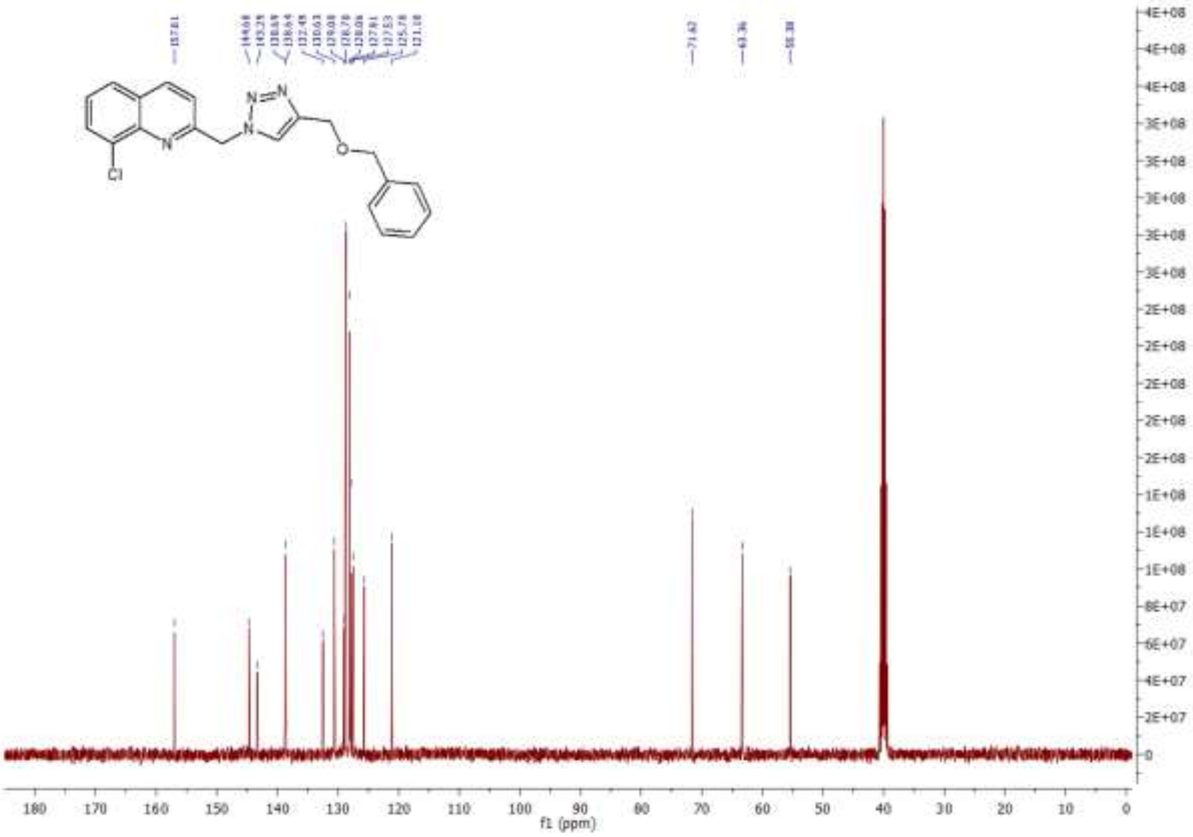
Cl
f1 (ppm)

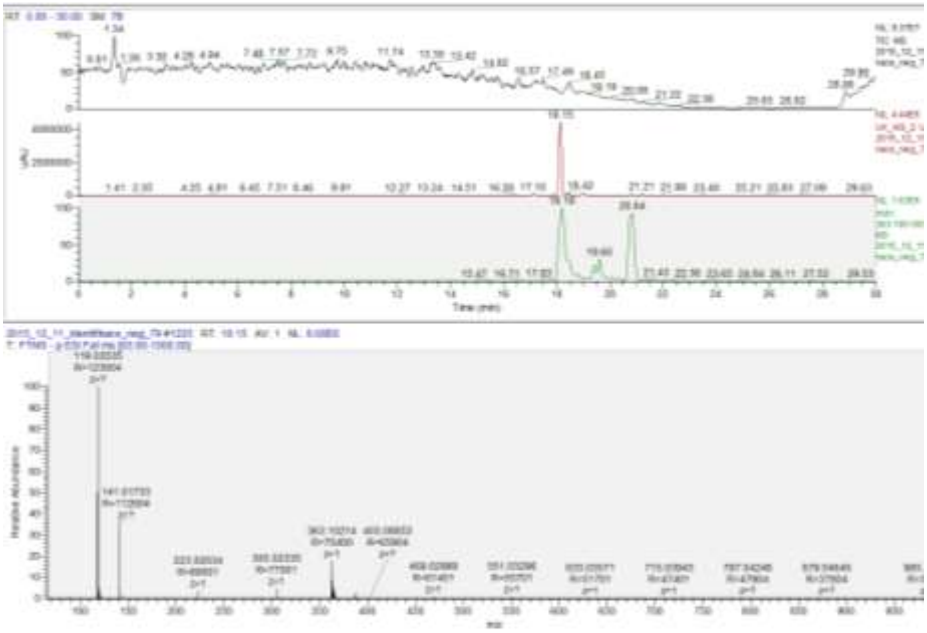

4-{1-[(8-chloroquinolin-2-yl)methyl]-1*H*-1,2,3-triazol-4-yl}-*N*,*N*-dimethylaniline (**7f**)

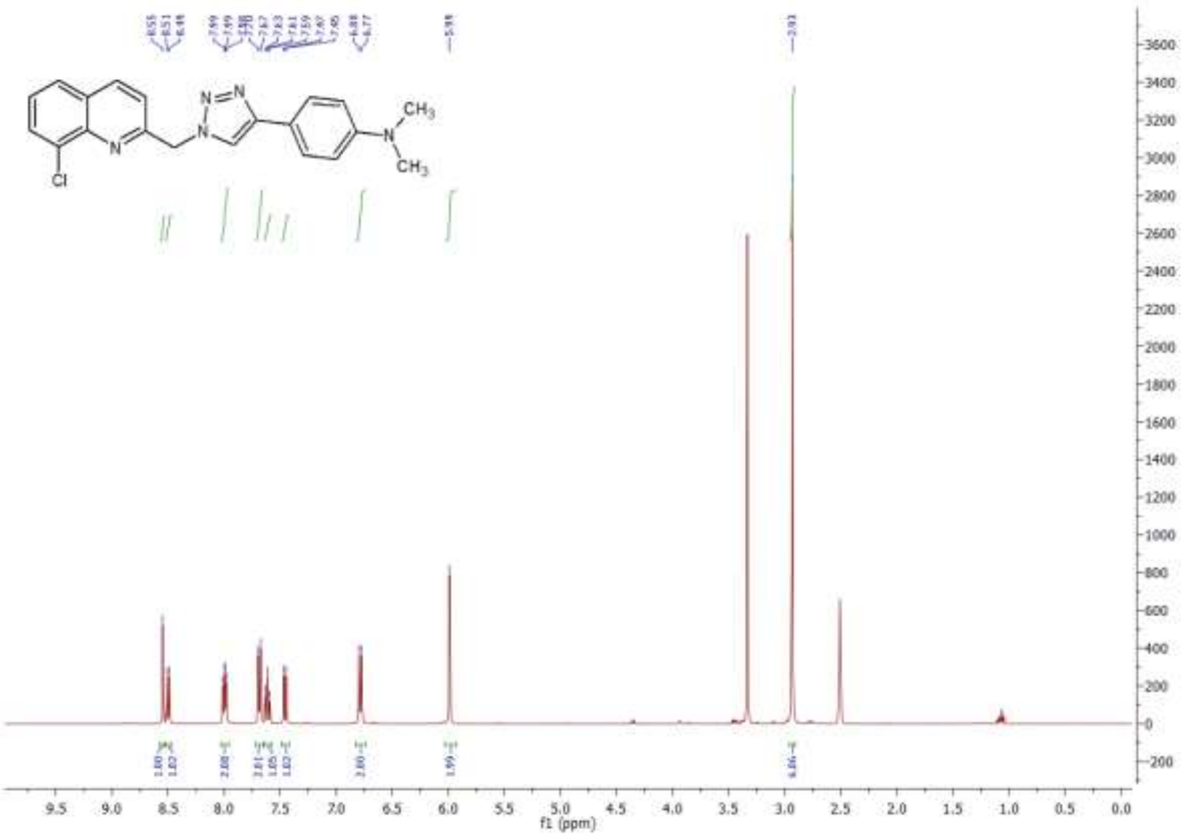

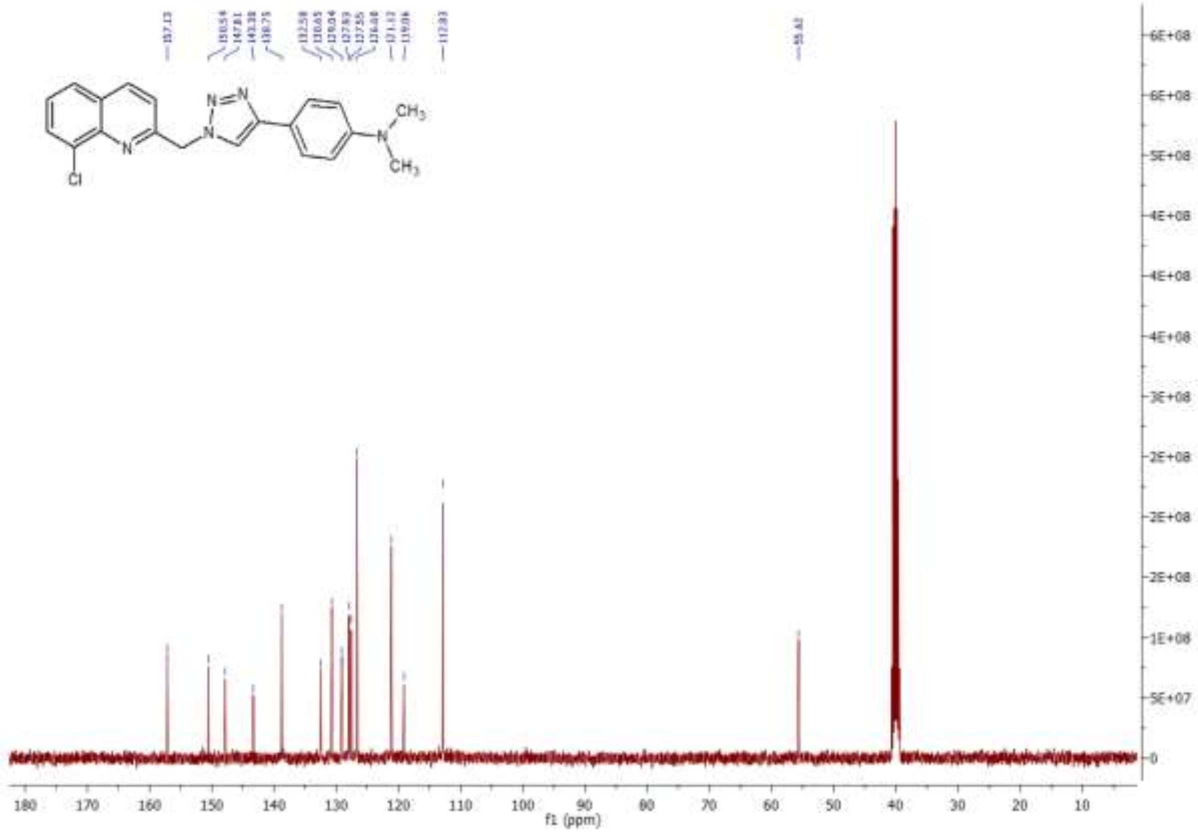

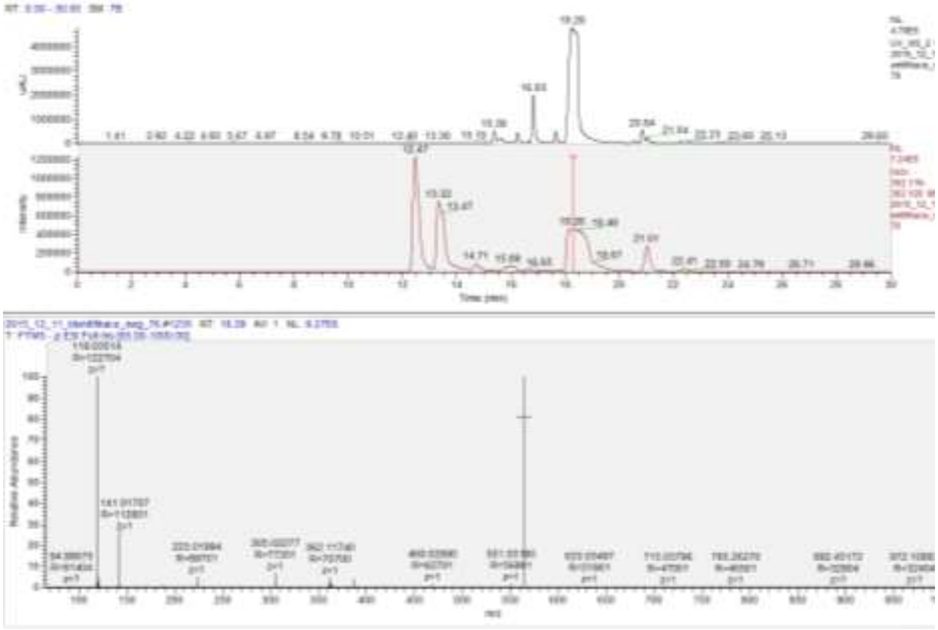

2-[(4-phenyl-1H-1,2,3-triazol-1-yl)methyl]quinazolin-4(3H)-one (**8a**)

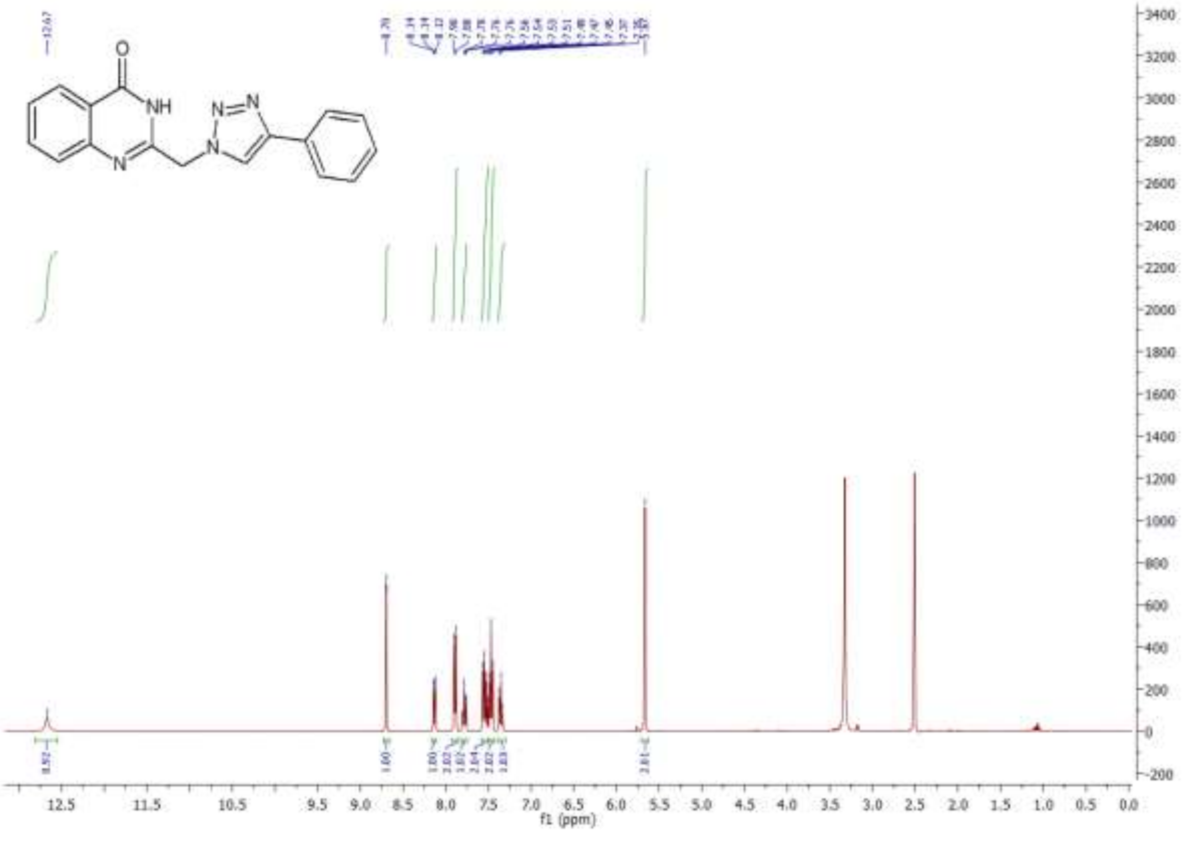

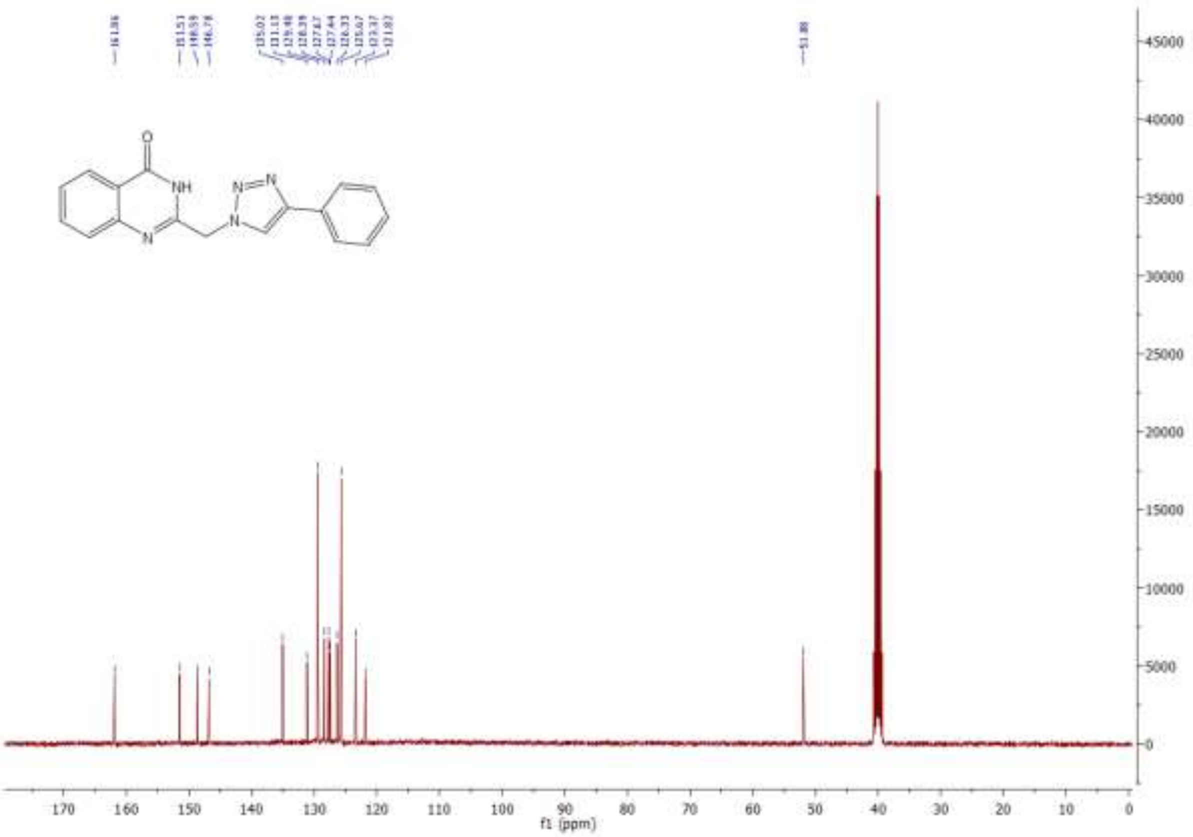
161.86
151.51
148.59
146.78
135.02
131.13
129.48
128.39
127.67
127.44
126.33
125.67
123.37
121.83
51.88
O
NH
N
N=N
N
45000
40000
35000
30000
25000
20000
15000
10000
5000
0
170 160 150 140 130 120 110 100 90 80 70 60 50 40 30 20 10 0
f1 (ppm)

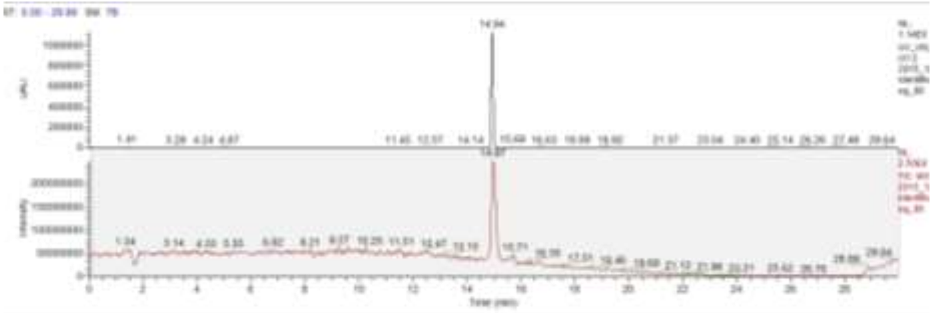

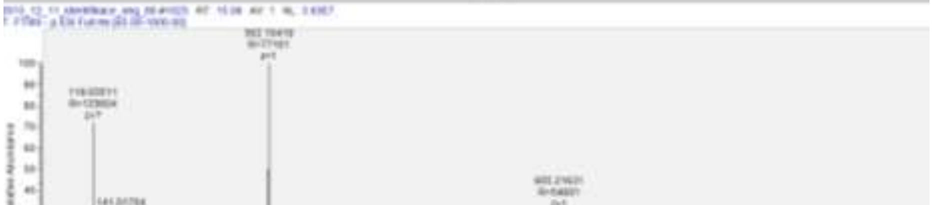

2-[(4-butyl-1H-1,2,3-triazol-1-yl)methyl]quinazolin-4(3H)-one (**8b**)

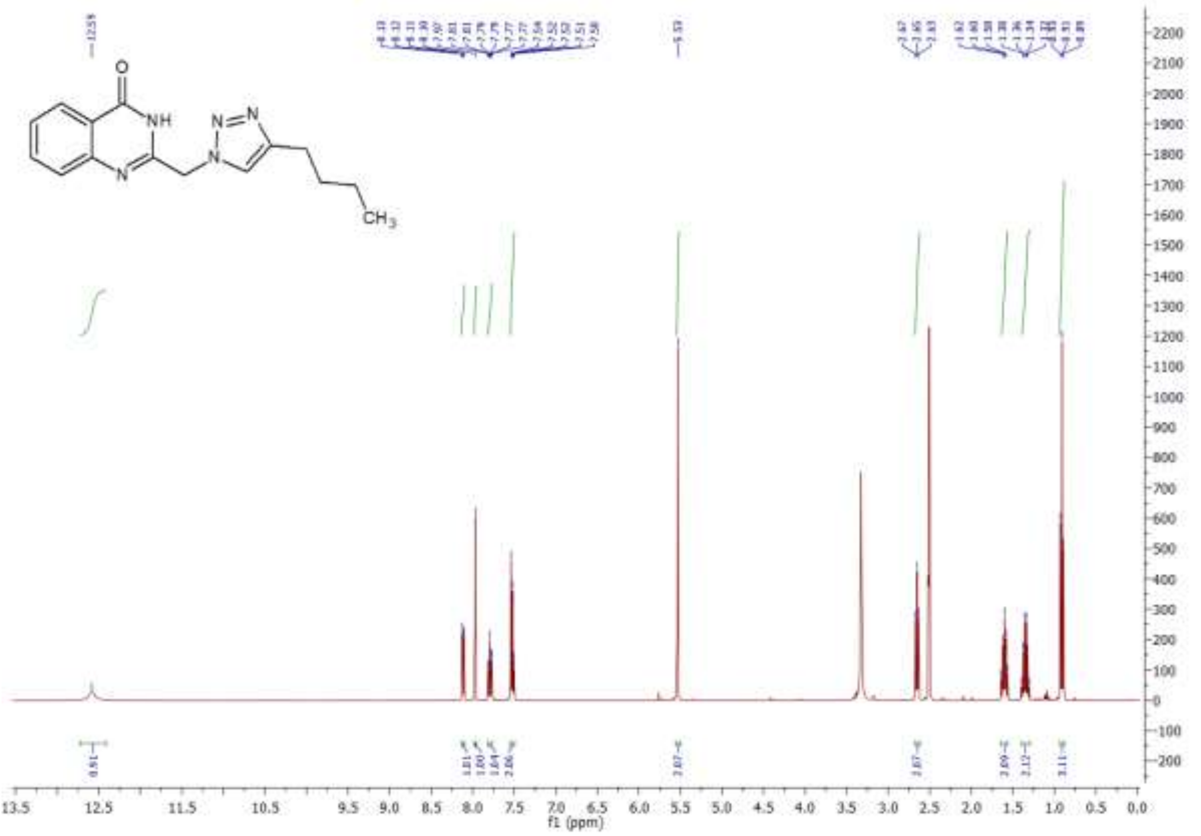

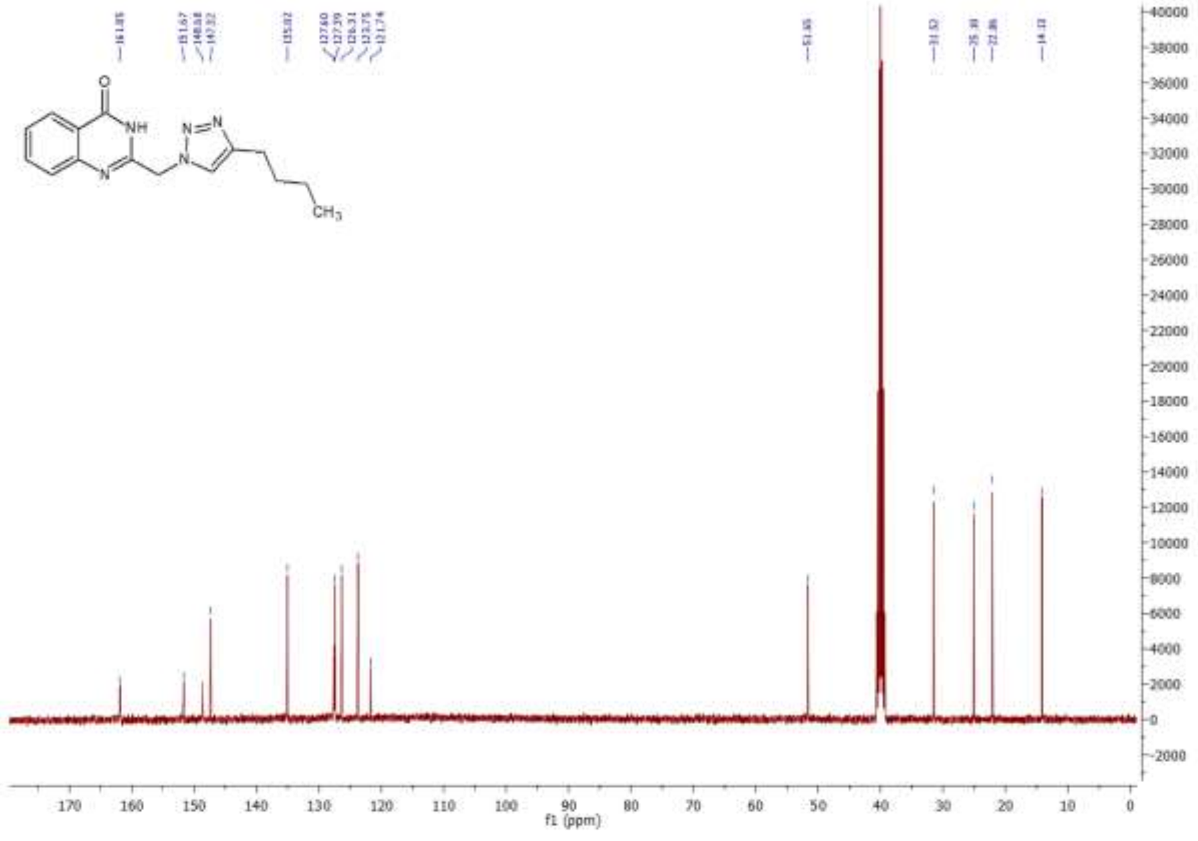

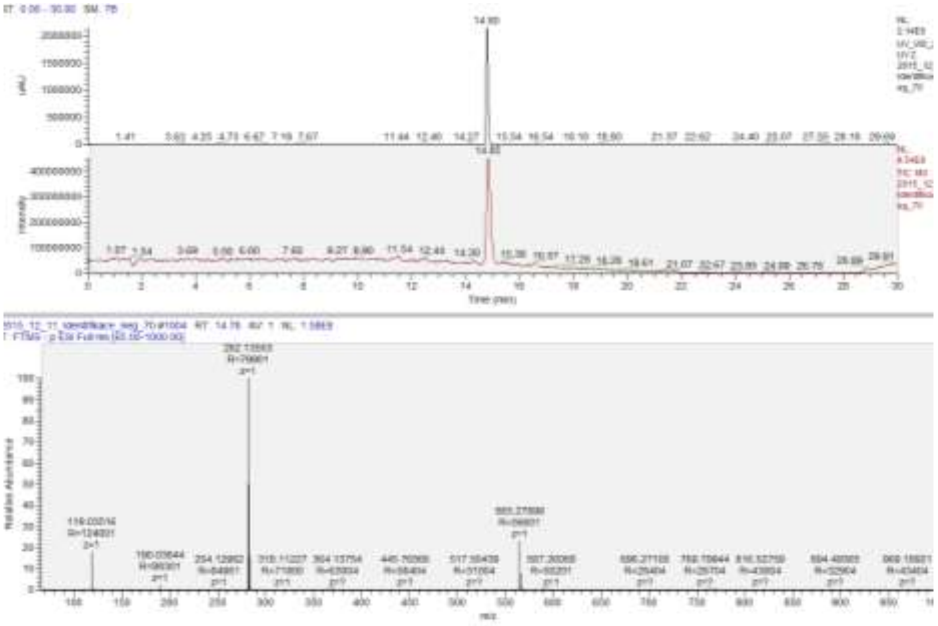

2-{[4-(hydroxymethyl)-1H-1,2,3-triazol-1-yl]methyl}quinazolin-4(3H)-one (**8c**)

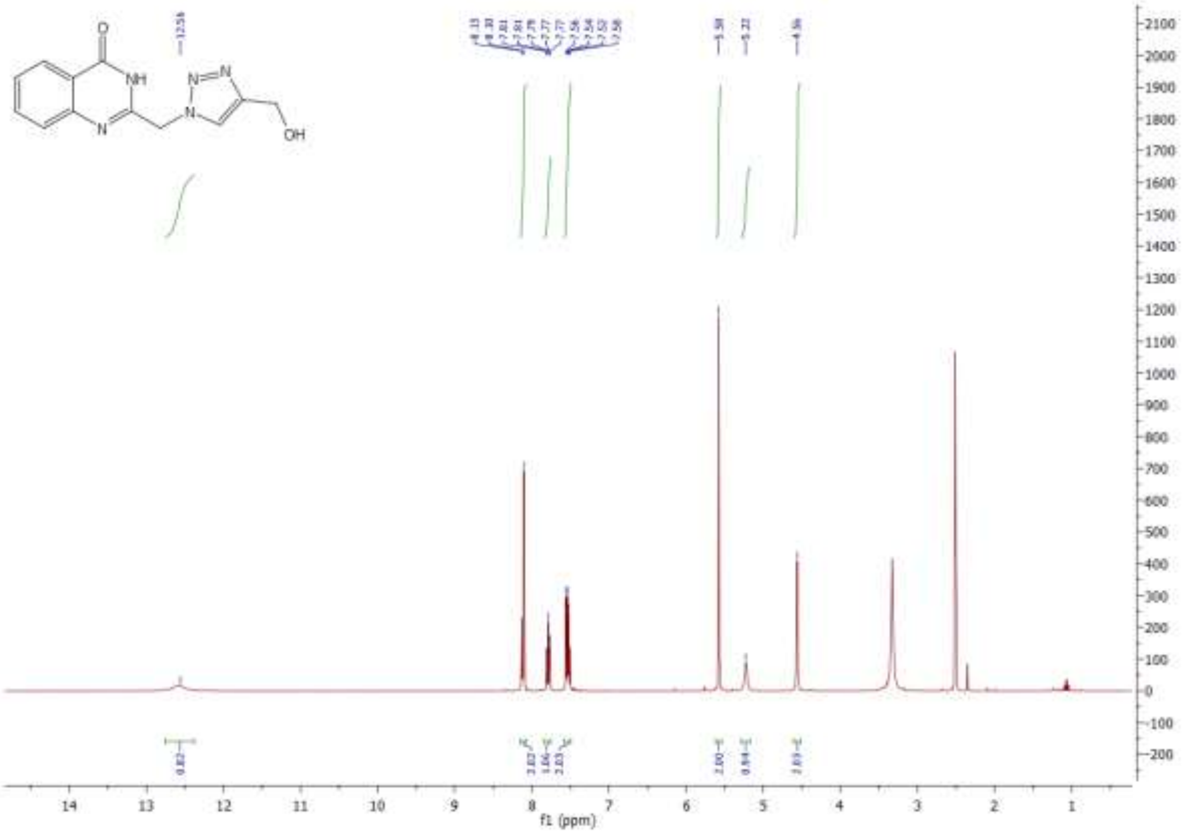

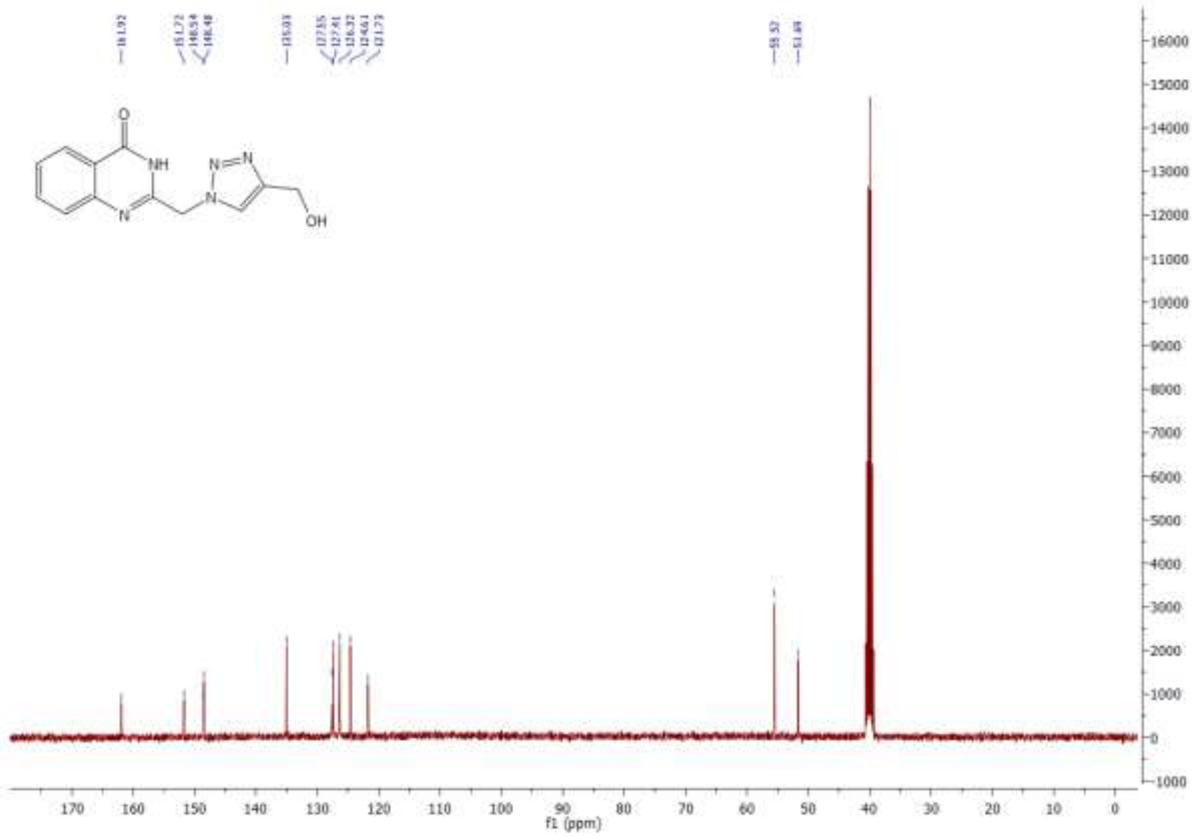
f1 (ppm)

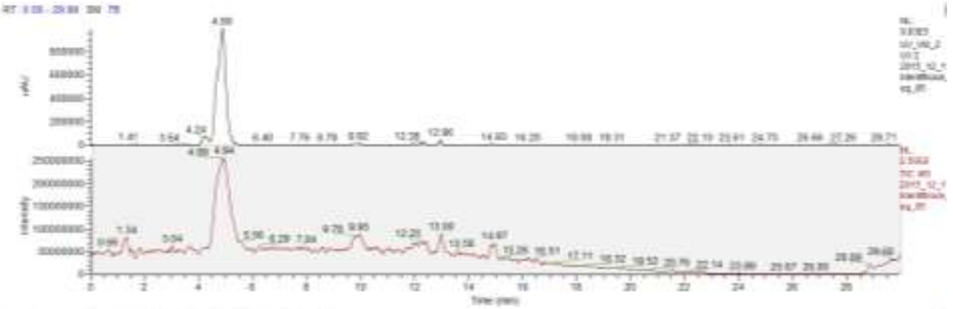

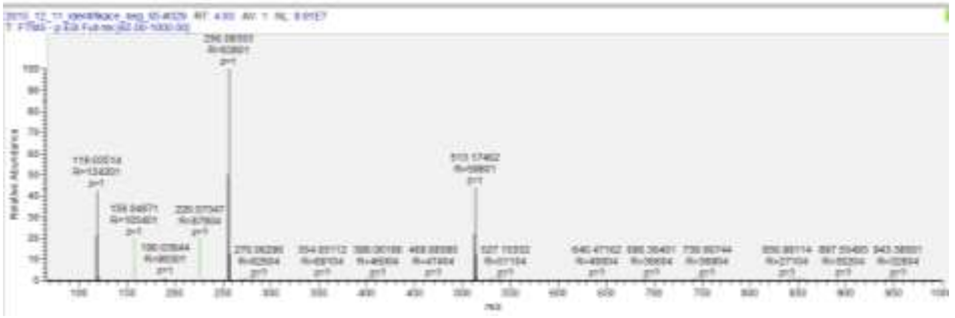

2-{[4-(1-hydroxyethyl)-1H-1,2,3-triazol-1-yl]methyl}quinazolin-4(3H)-one (**8d**)

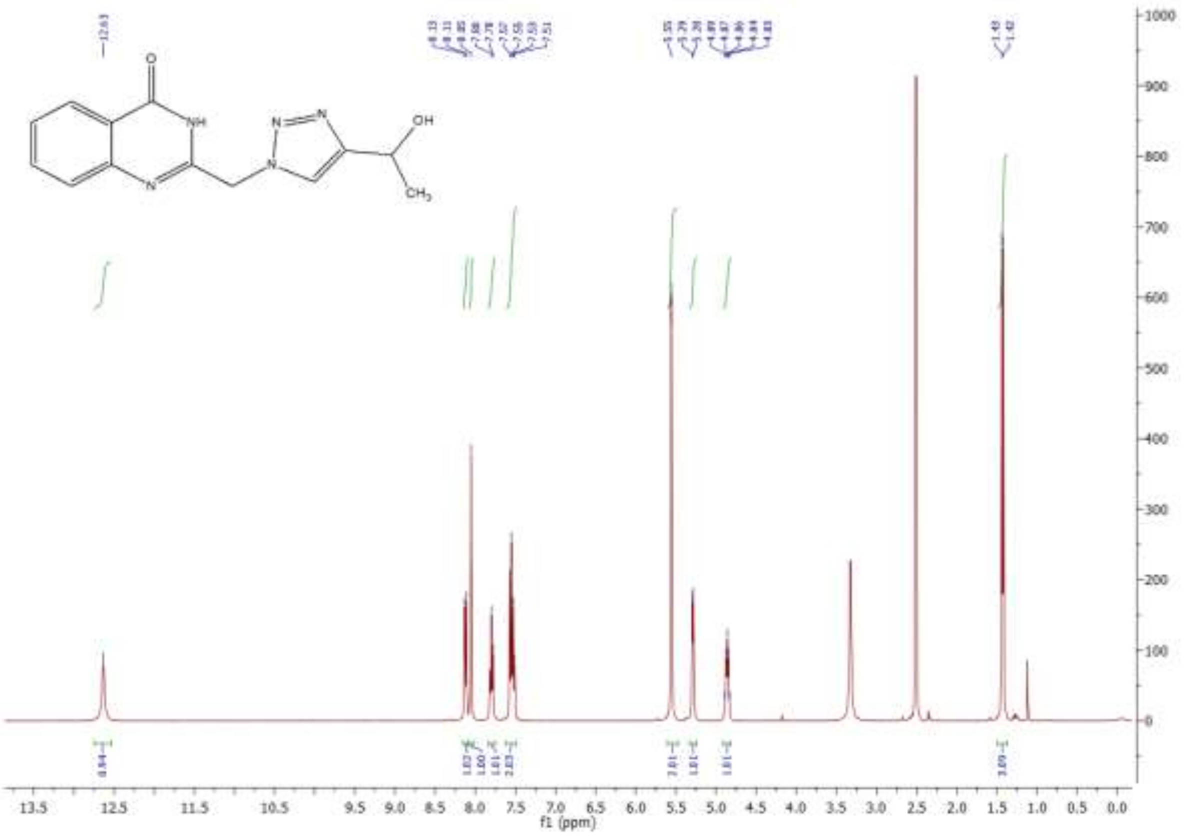

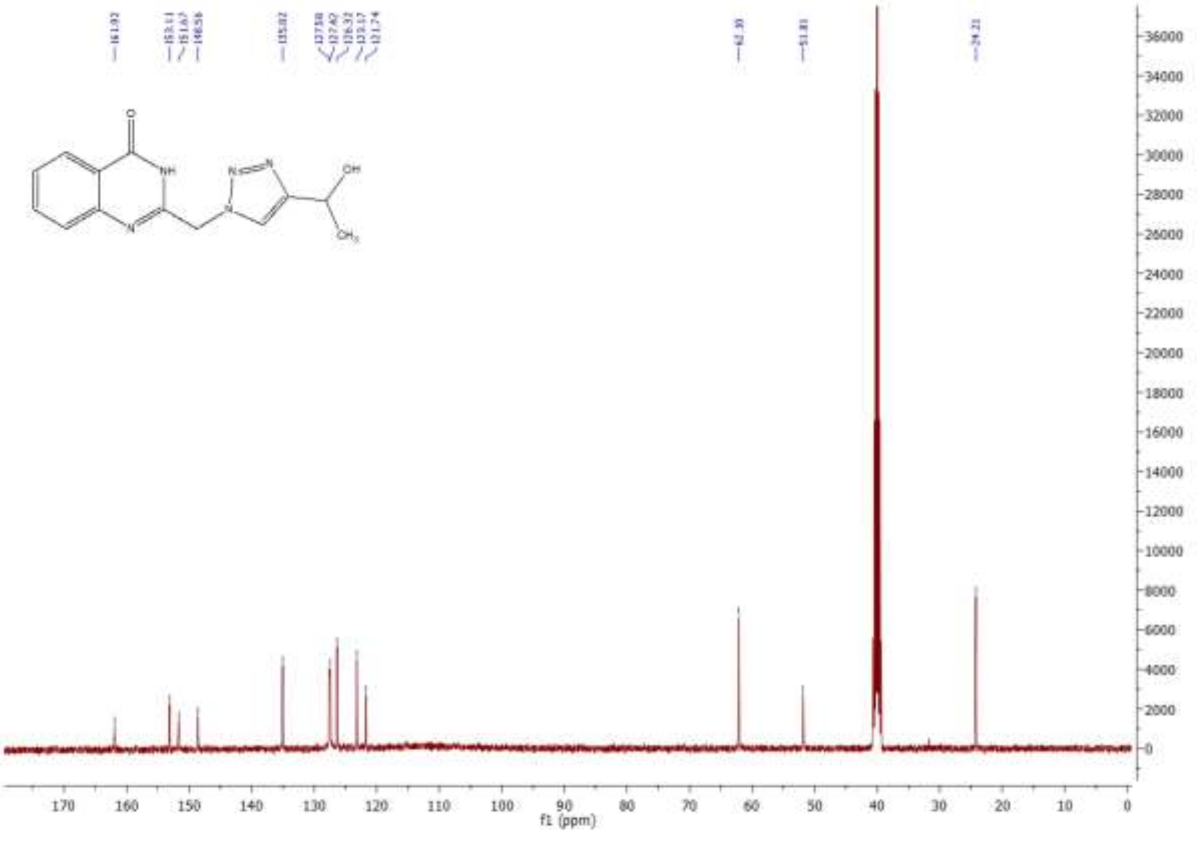

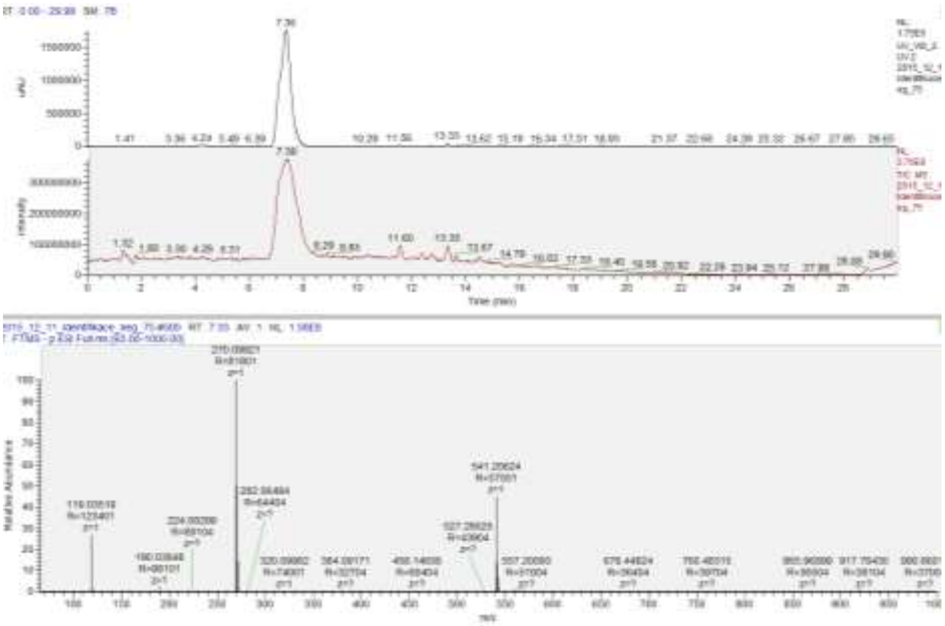

2-({4-[(benzyloxy)methyl]-1H-1,2,3-triazol-1-yl}methyl)quinazolin-4(3H)-one (**8e**)

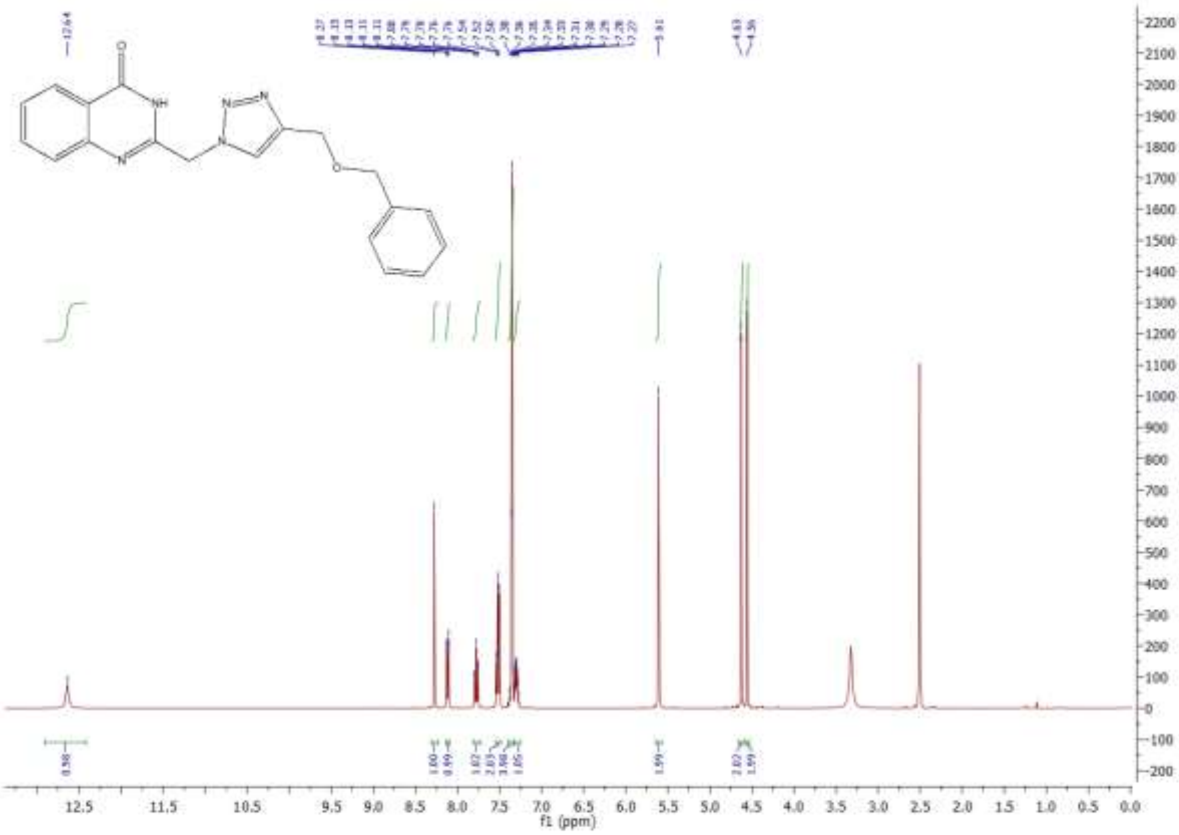

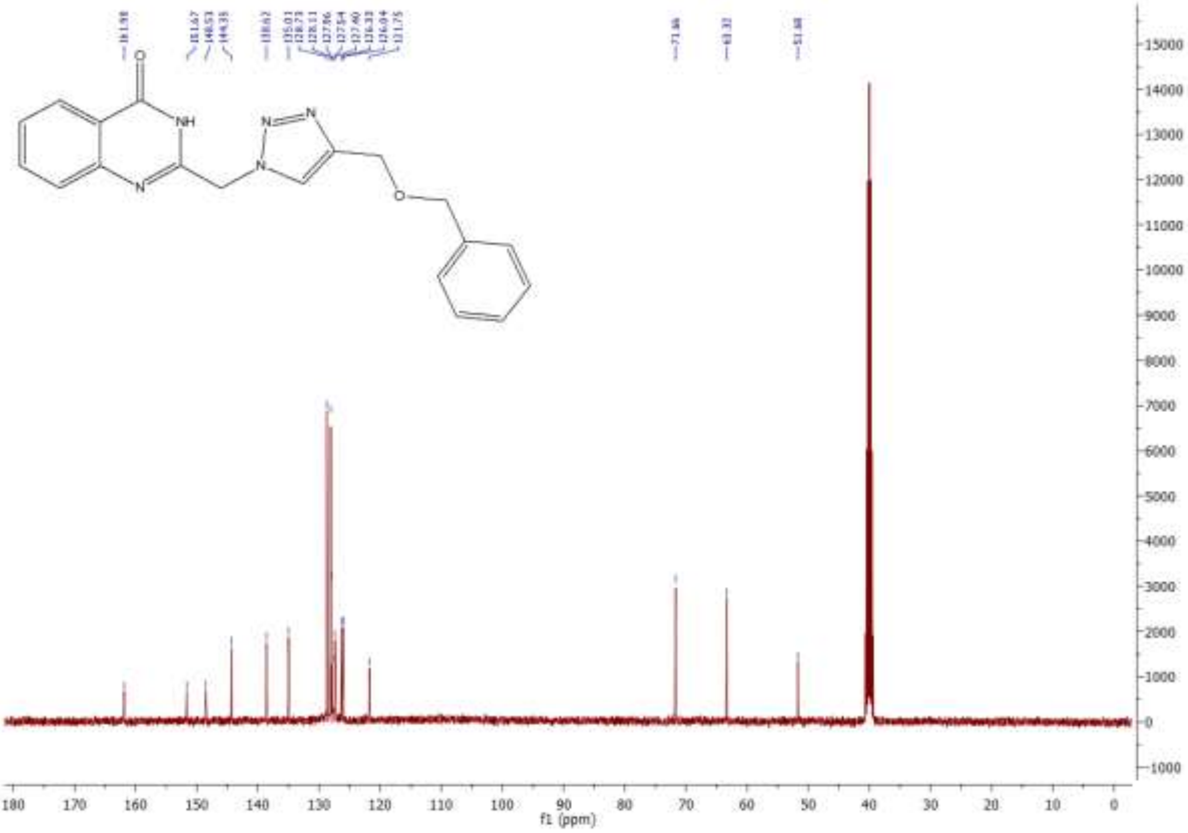
O
NH
N
N=N
N
O
f1 (ppm)

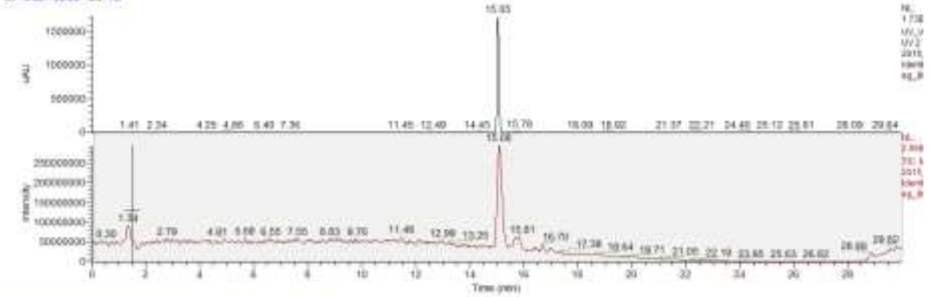
Time (min)

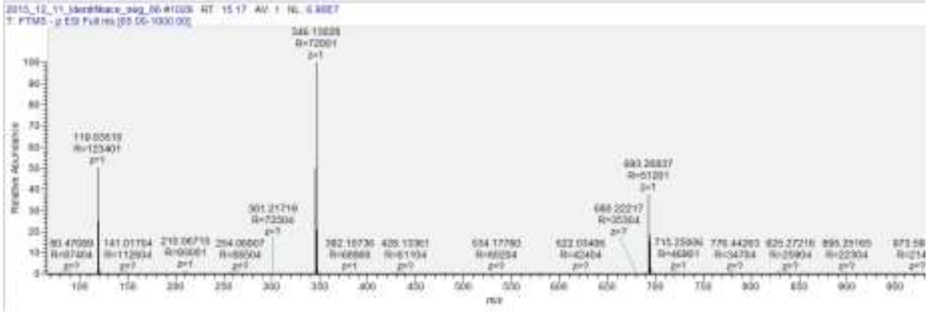
346.15035
Relative Abundance
m/z

2-({4-[4-(dimethylamino)phenyl]-1*H*-1,2,3-triazol-1-yl}methyl)quinazolin-4(3*H*)-one (**8f**)

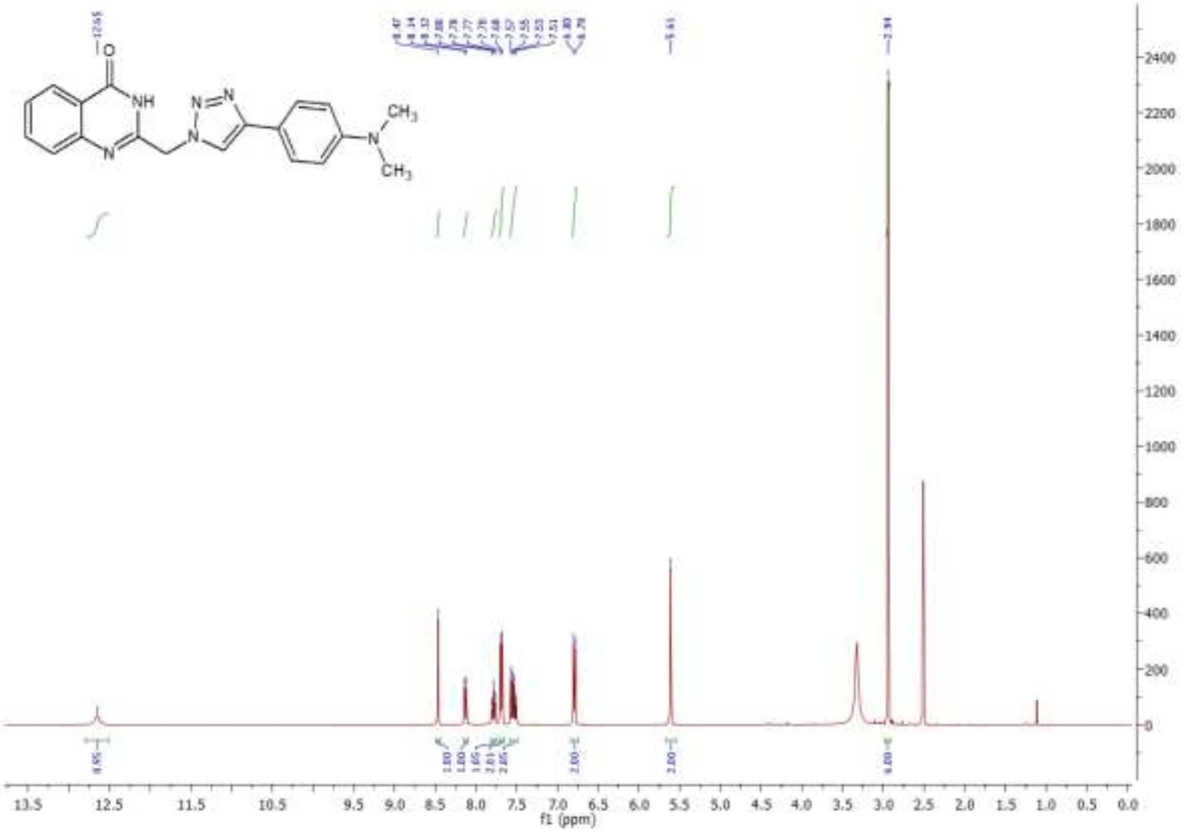

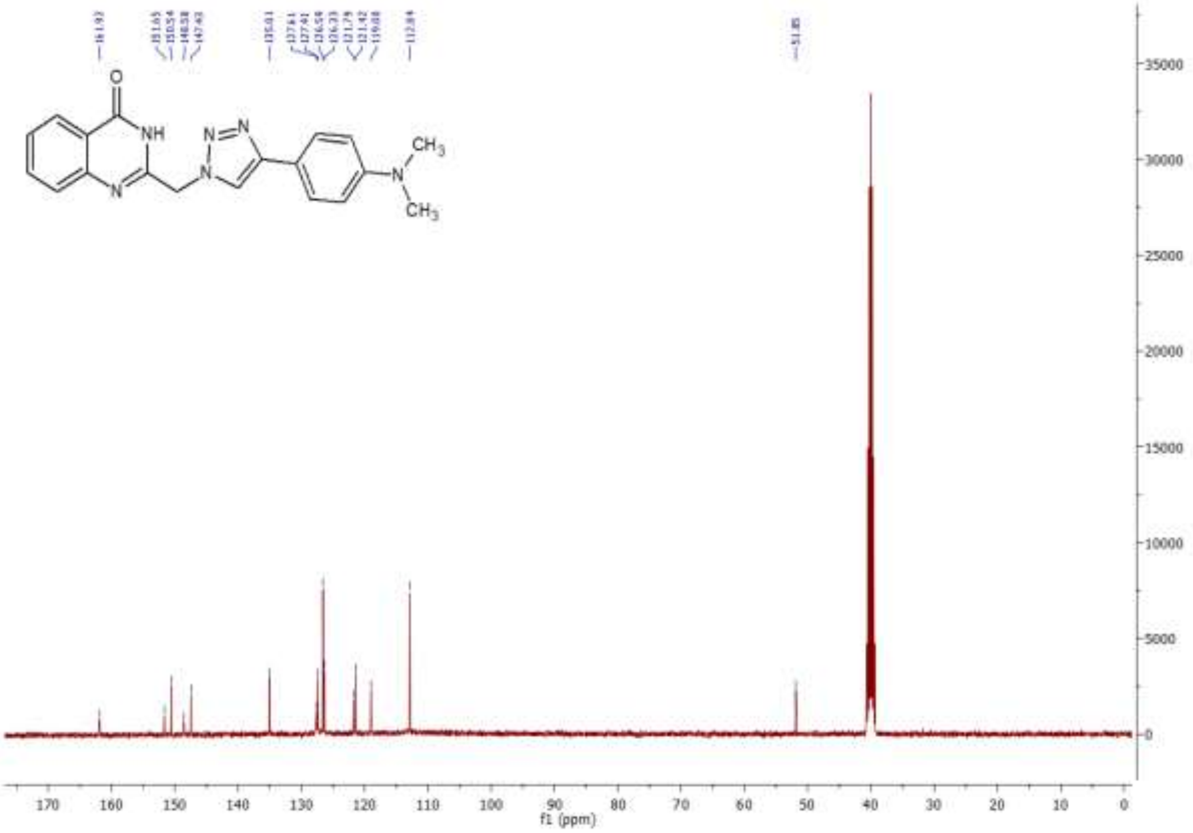

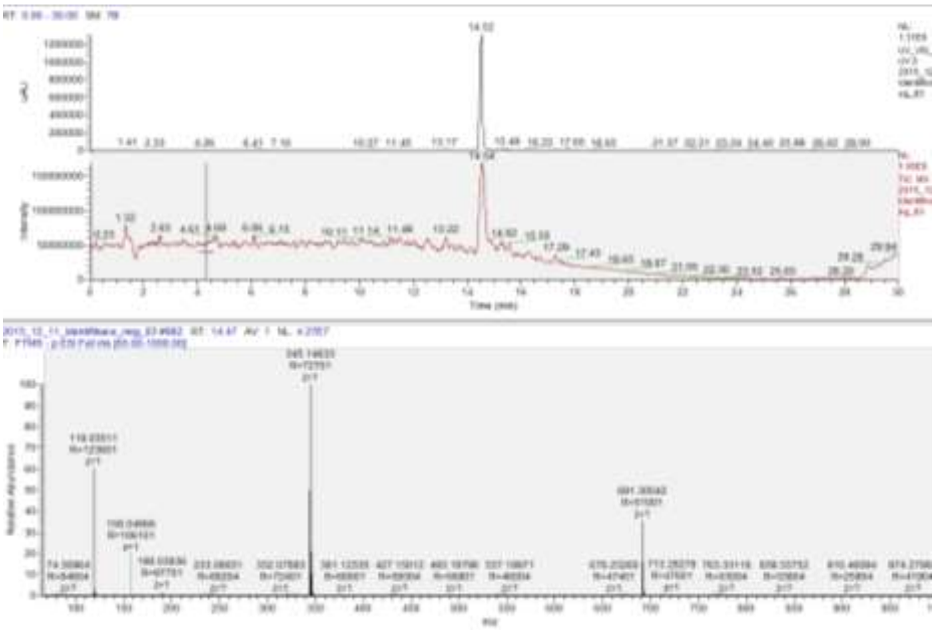

2-(iodomethyl)-3H-quinazolin-4-one (**9**)

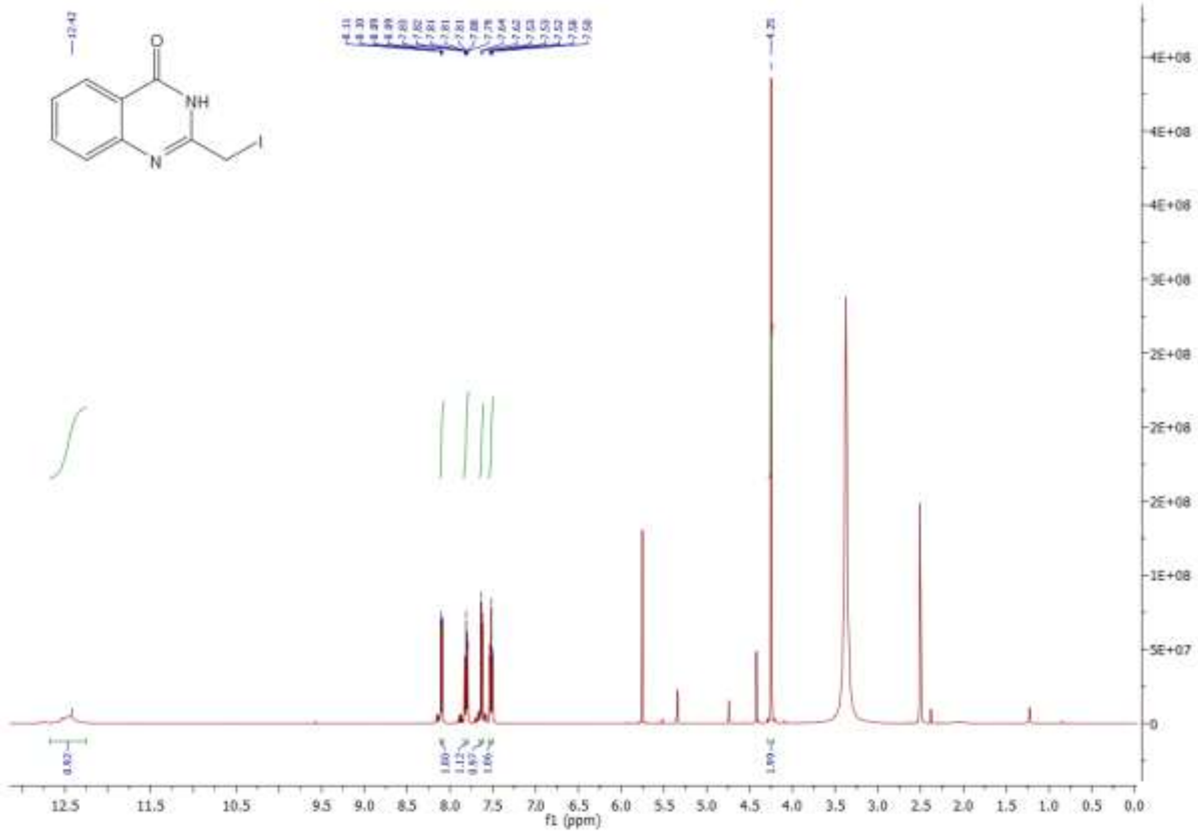

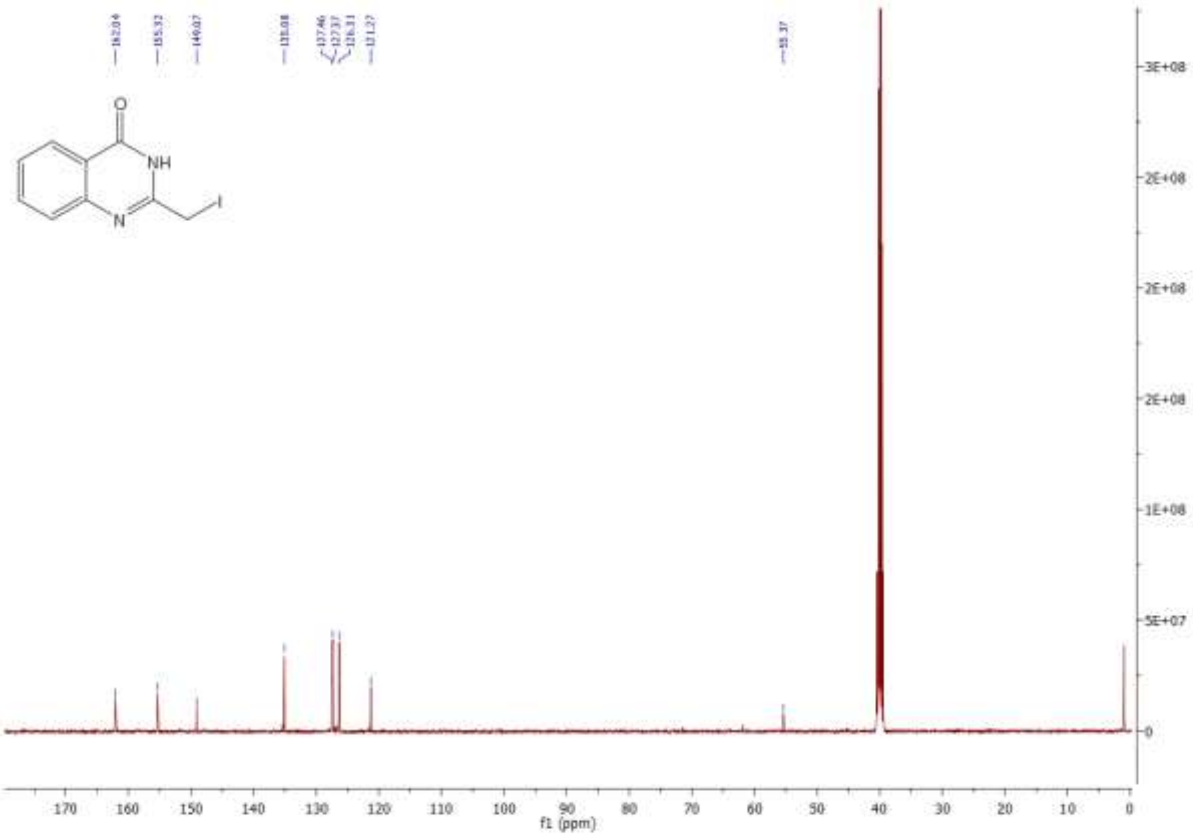
162.04
155.32
149.07
135.08
127.46
127.37
126.31
121.27
55.37
O
NH
N
I
3E+08
2E+08
2E+08
2E+08
1E+08
5E+07
0
170 160 150 140 130 120 110 100 90 80 70 60 50 40 30 20 10 0
f1 (ppm)

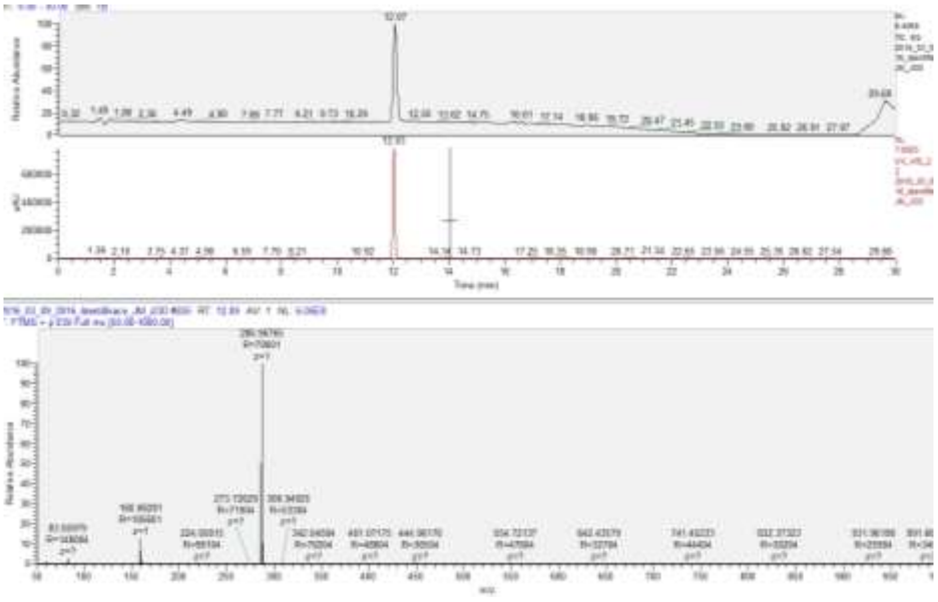

Supplement: Supplementary file 1 — Supplementary Information [file 41598_2018_22703_MOESM1_ESM.pdf]
